# Normative data on measures of cardiovascular autonomic neuropathy and the effect of pretest conditions in a large Danish non-diabetic CVD-free population from the Lolland-Falster Health Study

**DOI:** 10.1007/s10286-024-01069-6

**Published:** 2024-10-17

**Authors:** Christian S. Hansen, Marie Mathilde Bjerg Christensen, Dorte Vistisen, Randi Jepsen, Christina Ellervik, Marit Eika Jørgensen, Jesper Fleischer

**Affiliations:** 1 Translational Type 2 Diabetes Research, Steno Diabetes Center Copenhagen, Borgmester Ib Juuls Vej 83, 2730 Herlev, Denmark; 2 Clinical Epidemiology Research, Steno Diabetes Center Copenhagen, Herlev, Denmark; 3 Department of Public Health, Aarhus University, Aarhus, Denmark; 4 Novo Nordisk A/S, Søborg, Denmark; 5 Lolland-Falster Health Study, Nykøbing F. Hospital, Region Zealand, Nykøbing Falster, Denmark; 6 Department of Clinical Medicine, University of Copenhagen, Copenhagen, Denmark; 7 Department of Clinical Biochemistry, Zealand University Hospital, Køge, Denmark; 8 Department of Laboratory Medicine, Boston Children’s Hospital–Harvard Medical School, Boston, MA USA; 9 Steno Diabetes Center Greenland, Nuuk, Greenland; 10 National Institute of Public Health, University of Southern Denmark, Odense, Denmark; 11 Steno Diabetes Center Aarhus, Aarhus University Hospital, Aarhus, Denmark; 12 Steno Diabetes Center Zealand, Holbæk, Denmark

**Keywords:** Cardiovascular autonomic neuropathy, Cardiovascular disease, Neuropathy, Nomative values, Heart rate variability, Cardiovascular autonomic reflex tests

## Abstract

**Purpose:**

Cardiovascular autonomic neuropathy (CAN) is a common diabetic complication associated with excess morbidity and mortality. CAN is also seen in conditions such as Parkinson’s disease. Normative reference data for cardiovascular autonomic function are used to stratify individuals into those with and without CAN. However, reference thresholds for both cardiovascular autonomic reflex tests (CARTs) and heart rate variability (HRV) are scarce and based on small sample sizes. The aim of the study was to establish contemporary normative reference thresholds based on a large non-diabetic population free of cardiovascular disease (CVD).

**Methods:**

Cardiovascular autonomic function, CARTs and 5-min HRV indices were assessed in individuals without diabetes and CVD from the Lolland-Falster Health Study (2018–2020) by applying the point-of-care device Vagus™. Age-specific normative reference thresholds were estimated by using log-transformed quantile regression models at the 5th and 10th percentile, with adjustments made for sex. Models assessing the association between age and HRV indices were further adjusted for heart rate.

**Results:**

We present age-specific normative reference thresholds for cardiovascular autonomic function, including CARTs and HRV, for 875 individuals (48% females) aged 15–85 years. The reference thresholds are presented for both the 5th and 10th lower percentile. Higher age was inversely associated with all outcomes. Females tended to have a higher parasympathetic drive compared to males. Pre-test conditions did not affect CARTs significantly.

**Conclusions:**

The presented age-related normative reference thresholds for both CARTs and HRV indices based on a large Danish cohort may facilitate improved quality of research and treatment.

**Supplementary Information:**

The online version contains supplementary material available at 10.1007/s10286-024-01069-6.

## Introduction

Cardiovascular autonomic neuropathy (CAN) is a condition caused by damage to the autonomic nerve fibres that innervate the heart and blood vessels, resulting in abnormalities in heart rate control and vascular dynamics [1]. It is a common diabetes complication, affecting 20–60% of people with diabetes [2]. In diabetes, CAN is an independent risk factor for cardiovascular mortality and morbidity [3–5], as exemplified by a threefold higher mortality in people with diabetes and CAN compared to those without CAN [6], as well as a twofold higher risk of silent myocardial ischaemia [7]. CAN is also a complication of Parkinson’s disease (PD), with 30–40% of patients with PD showing signs of CAN [8]. In addition, measures of CAN have been used as markers of psychological stress and depression [9] and as a disease severity marker in conditions such as infectious disease [10].

Reliable reference values for CAN measures are paramount for a correct and early diagnosis. For example, optimal diabetes treatment has been shown to reverse the early stages of CAN [11] and reduce the progression of the complication [12]. Here, an early and correct diagnosis with the threshold values discussed further in this article may lead to interventions before severe stages of the condition have manifested.

The tests recommended for diagnosing CAN in diabetes are based on either blood pressure changes in response to postural changes or cardiovascular autonomic reflex tests (CARTs) which assess heart rate changes in response to physical challenges. For the latter, it is recommended to assess heart rate changes in response to changing position from lying to standing, to deep breathing and to the Valsalva manoeuvre [13]. Existing normative reference thresholds for these three CARTs are scarce and based on relatively few healthy participants with limited age ranges [14–16].

Assessment of cardiovascular autonomic dysfunction by use of short-term heart rate variability (HRV) indices derived from resting electrocardiogram (ECG) traces has been suggested as a means of assessing the early stages of CAN. HRV indices can provide insights into which parts of the autonomic nervous system may be affected. These indices have not been amended into the battery of tests used for diagnosing CAN, possibly, in part, due to the lack of normative data based on large cohorts of healthy people. Few efforts have been made to define normal ranges of HRV indices, and those studies which have been conducted have been performed either on relatively small cohorts [14, 17] using very short-term measures (< 5 min) [18], on long-term Holter monitor data [19] or in cohorts with relatively narrow age intervals [20]. These approaches cannot be translated into applicable standards for short-term HRV assessment. In addition, the relatively small cohorts in previous studies may hamper the generalisability to other populations.

With the study reported here, we hope to improve the diagnostic accuracy of CARTs and facilitate the use of HRV indices by presenting much-needed new normative data on the CARTs recommended for diagnosing CAN and on the most commonly used HRV indices in both the time and frequency domains.

## Methods

### Study population

This cross-sectional normative reference study is a sub-study of the Lolland-Falster Health Study (LOFUS). LOFUS was an epidemiological prospective cohort study (data were collected between 2016 and 2020) with the overall aim to assess health in the rural-provincial south-eastern part of Denmark. People residing within this geographical region were randomly selected from the Danish Civil Registration System and invited to participate in a survey. The survey included questionnaire data on self-assessed physical and mental health and lifestyle factors, a physical examination and a collection of biological samples. Invitational emails were issued via the electronic personal mailbox (Digital Post system of Danish public authorities); this e-box is mandatory for people in Denmark aged ≥ 15 years. Non-responders were re-approached by re-invitations and thereafter by phone. LOFUS is described in detail elsewhere [21]. Participants enrolled in LOFUS (between 2018 and 2020 at the study site in the town Nykøbing Falster) were randomly selected to undergo an examination of cardiovascular autonomic function (described below). The selection was, however, stratified to aspire to an equal representation of both sexes and all 10-year age groups from 15–85 years. Participants were excluded if they reported being pregnant or having diabetes, ongoing cancer, pacemaker or known heart disease, including atrial fibrillation, heart failure or coronary artery disease. Moreover, participants were excluded if they were identified with thyroid dysregulation (thyroid stimulating hormone [TSH] < 0.3 IU/L and TSH > 4.0 IU/L). As beta-blockers may reduce cardiac sympathetic activity and thereby increase HRV, participants treated with beta-blockers were excluded [16]. Data on the effect of other antihypertensive medications on cardiovascular autonomic function are sparse, so participants treated with these medications were not excluded. Participants who reported using antidepressants or psychotropic drugs that are known to potentially reduce HRV were also excluded. No restrictions were imposed on the consumption of caffeine and food or physical activity before the examination. Overall, 131 participants reported having hypertension, with 114 of them reported being treated with antihypertensive medication (beta-blockers not included in this group).

Approval of the study was obtained from Region Zealand’s Ethical Committee on Health Research (SJ-421) and The Danish Data Protection Agency (P-2020-614). Written informed consent was obtained from all participants. LOFUS is registered at Clinicaltrials.gov (NCT02482896).

### Measures of cardiovascular autonomic function

Participants visited the study site once, in a non-fasting state, at a scheduled time between 7:30 a.m. and 6:30 p.m. from Monday to Thursday. Certified nurses and biomedical laboratory technicians carried out the procedures.

It was recorded if participants had a larger meal and/or had caffeine intake within 3 h before the examination. In addition, it was registered if participants had performed strenuous physical activity within 24 h before the examination. There were no restrictions imposed on these factors. Examinations were performed in a quiet room at room temperature.

The three CARTs and short-term HRV indices were obtained once by applying the handheld device Vagus™ (Medicus Engineering, Aarhus, Denmark), which is a one-lead ECG recording device that via a display guides the user through the various tests. The CARTs and HRV measures were performed by instructions given by the study staff and by dynamic real-time instructions on the display of the device. For the latter, the device displays, for example, when the pressure for the Valsalva manoeuvre is reached and at what pace the deep breathing should be carried out during the deep-breathing test. The device has been described in detail previously [22–25]. Staff had received training in performing the measurements, and colleagues and managers monitored adherence to the procedure on an ongoing basis.

Following 5 min of rest in a supine position, data on R-R intervals were obtained by a resting 5-min supine recording. An autoregressive model approach was used in the spectral analysis of HRV. Indices were analysed in both the time domain and frequency domain. Time domain measures included the standard deviation of normal-to-normal intervals (SDNN) and the root mean square of successive differences between normal-to-normal heartbeats (RMSSD). Frequency domain metrics included low-frequency power band (LF) (0.04–0.15 Hz) and high-frequency power band (HF) (0.15–0.4 Hz) and total frequency power [26, 27].

Subsequently, the CARTs were performed: lying-to-standing, deep breathing and Valsalva manoeuvre. The results of the CARTs are expressed as ratios of the minimum to the maximum R-R interval during the specific tests. In addition, we also estimated the LF and HF power during the three CARTs as performed previously to provide HRV indices that may be more reliable [28]; for example, during deep breathing, which may induce respiratory sinus arrhythmia, HF power is expected to increase due to enhanced parasympathetic activity. This controlled environment may make the level of LF and HF power more representable and less influenced by various external factors.

#### Lying-to-standing (30:15 ratio)

Following the 5-min rest and 5-min measurement of HRV in a supine position, the participants stood up quickly after which the ECG recording was initiated. The result of the test is expressed as the ratio of the minimal (around the 15th heartbeat) to the maximum (around the 30th heartbeat) R-R interval. The device is able to search for the minimal R-R interval from the fifth to 25th heartbeat and the maximum R-R interval from the 20th to 40th heartbeat.

#### Deep breathing ratio (E:I ratio)

The participants were guided to breathe deeply 6 times for 1 min while sitting in an upright, relaxed position. The ratio between the average of the longest R-R intervals during expiration and the average of the shortest R-R intervals during inspiration was calculated.

#### Valsalva manoeuvre (Valsalva ratio)

The participants blew into a mouthpiece for 15 s under continuous expiratory pressure. The device was able to measure whether an intrathoracic strain pressure of 40 mmHg was maintained. Subsequently, the strain was released, and the breathing was normalised for 45 s. The ratio between the shortest R-R interval during the expiratory straining and the longest R-R interval after pressure release was measured [16, 29].

### Post-recording data handling

All CART measurements were examined for outliers by visualising the data in a scatterplot. Extreme values were visually identified where a maximum of four to five measures were distinguishably higher than the rest. The raw R-R interval data of the specific measurement was examined using Vagus™ Cloud software (Vagus Technologies, Tiruchirappalli, Tamil Nadu, India). If the outlying value most likely resulted from a measurement error or an irregular heart rate, the specific observation was excluded.

For HRV measures, R-R interval errors were identified by assessing the level of SDNN and RMSSD. If the SDNN was relatively high (arbitrarily set to > 80 ms) and lower than the RMSSD, the specific measurement was visually inspected in Vagus™ Cloud. The specific measurement was excluded if a large time period of the R-R intervals was without a clear pattern, indicating irregularities in the heart rhythm or noise.

### Biological samples

Non-fasting blood and urine samples were collected. Blood samples were centrifuged and kept at 21 °C during transportation and until analysis at Nykøbing Falster Hospital on the same evening.

Haemoglobin A1c (HbA1c) was measured from whole blood and analysed by high-performance liquid chromatography (International Federation of Clinical Chemistry [IFCC] method) on a TOSOH-G8 HPLC Analyzer (Tosoh Cooperation, Tokyo,Japan). Plasma cholesterol and urinary albumin-to-creatinine ratio were measured using the Siemens Dimension Vista 1500A System (Siemens Healthcare Diagnostics, Newark, DE, USA). Low-density lipoprotein (LDL) cholesterol was calculated by applying the Friedewald equation [30] when the plasma triglyceride concentration was < 4.5 mmol/L. The Chronic Kidney Disease Epidemiology (CKD-EPI) equation was used to estimate the estimated glomerular filtration rate (eGFR) [31].

### Blood pressure and anthropometric measures

The systolic and diastolic blood pressure measurements were obtained after 5 min of rest by sampling three consecutive digital measurements on the upper left arm (Welch Allyn Connex proBPO 3400 Monitor; Welch Allyn, Auburn, NY, USA) with the participant in a supine position. The mean values of the second and third measurements were utilised for each participant. If one measurement was missing, only the available measurement was used.

Height without shoes was measured with the participant in the standing position using the SECA 216 wall-mounted stadiometer (SECA Corp., Chino, CA, USA). Weight was measured in the non-fasting state using the Tanita Body Composition Analyzer BC-420MA III, the Tanita Body Composition Analyzer DC-430MA or the Tanita WB-110A digital medical scale (Tanita Corp., Tokyo, Japan). Body mass index (BMI) was determined by dividing the weight in kilograms by the height in meters squared (kg/m^2^).

### Questionnaire data

The participants filled in questionnaires that included questions on smoking status (never, former, current), physical activity (activity level during the last year: vigorous, moderate, light, sedentary) and alcohol consumption (the intake frequency of ≥ 5 units: never, rarely, weekly, monthly or daily), medicine intake and the presence of chronic conditions (such as cardiovascular disease [CVD], diabetes and cancer) at the time of participation in LOFUS.

### Statistical analysis

#### Missing data

For variables and outcomes with > 5% missing observations, dependency of the missingness on age and sex was examined using a logistic regression model with missingness as a binary outcome. A certain level of physical ability is required to complete the three CARTs. Therefore, not every participant was capable of performing all three tests. Older and fragile individuals may struggle with tests such as the lying-to-standing manoeuvre and Valsalva manoeuvre. As a result, there will be some degree of missing data for these specific outcomes, especially among individuals likely to have a poor result on the CART. Hence, missing data at random could not be assumed and imputation of missing data was not possible. Complete case analysis was therefore applied, and the study estimated a normative reference threshold for individuals who were able to successfully complete the tests.

#### Model selection

A quantile regression model was applied to estimate the normative thresholds of the continuous outcomes derived from the CARTs based on age and the HRV based on age and heart rate. The cut-off point of normality was defined as the lower fifth percentile, which represents a specificity of 95% based on the common practice of a 5% false-positive rate in statistical testing. The use of extreme percentiles maximises test specificity but may reduce sensitivity, and we anticipated that a low percentile will have the greatest clinical relevance to physicians and researchers. In cases where data are sparse at the extremes, the estimation of extreme percentiles may be associated with higher uncertainty and wider confidence intervals. Thus, we have also estimated the 10th percentile, which is more robust in terms of accuracy and precision but also has a lower specificity. Since no adverse effects of increasing levels are reported for both CARTs and HRV indices, there was no need to assess an upper threshold.

The results of the CARTs and the HRV measurement showed log-normal distribution. Hence, we applied a log transformation to the outcome indices of HRV, which ensured that the percentile estimates remained positive (> 0), as it is not biologically plausible for HRV measures to exhibit negative values. Similarly, for the CARTs, as the ratios cannot yield values between 0 and 1, we applied a log transformation to the ratio minus 1 (see electronic supplementary material [ESM] Appendix section 3).

Age was included in the models as the exposure parameter. Resting heart rate was added to the models estimating HRV outcomes. Bootstrapping was used to estimate standard errors, and a two-sided statistical significance level of 0.05 was applied. To assess a potential difference in the outcomes between males and females, sex was added as a covariate in additional models. Subsequently, we stratified our sample by sex and applied the same method as described above for the entire study population (ESM Appendix sections 4, 5).

#### Adjustment for multiple testing

The Benjamini–Hochberg procedure was applied to all models to control for multiple testing and reduce false positive results. We decided to apply this method as it is a less conservative method compared to, for example, the Bonferroni method. Since many of the outcomes are correlated to some degree, and the tests are not entirely independent, the Benjamini–Hochberg method is a reasonable choice as it balances the need to control false–positive results with maintaining statistical power. Moreover, because the method increases power, it is a suitable method to use in exploratory studies like the present study.

#### Age-specific estimates

For all the CART ratios, reference values with 95% confidence intervals (CIs) were derived for each 5-year age interval as the estimated value for the median age of that age interval; for example, the reference value for the 20- to 25-year age interval was the estimated 5th percentile value for the age of 22.5 years. For all HRV indices, the reference values were obtained for both 5-year age intervals and heart rate intervals of 10 bpm. The age-specific estimates were presented in tables in order to provide the user with a quick mean off assessing the normative reference value at an individual level.

#### Confounding effects of food and caffeine intake, physical activity and time of examination

To assess the potential confounding effect of food, caffeine and physical activity before the examination and the time of the examination (before or after noon) on the study outcomes, we evaluated the associations with the outcome measures of CARTs and HRV by applying linear regression models with the pretest condition as the determinant (binary variable) and autonomic test as outcome. The models were adjusted for age. As all outcome measures exhibited a skewed distribution, they were log-transformed before the estimation, and the estimates were subsequently back-transformed.

#### Software

Statistical analyses were performed in R Studio version 4.1.2 (R Foundation for Statistical Computing, Vienna, Austria). All applied packages are listed in ESM section 1.1.

## Results

### Study population

**Fig. 1:**
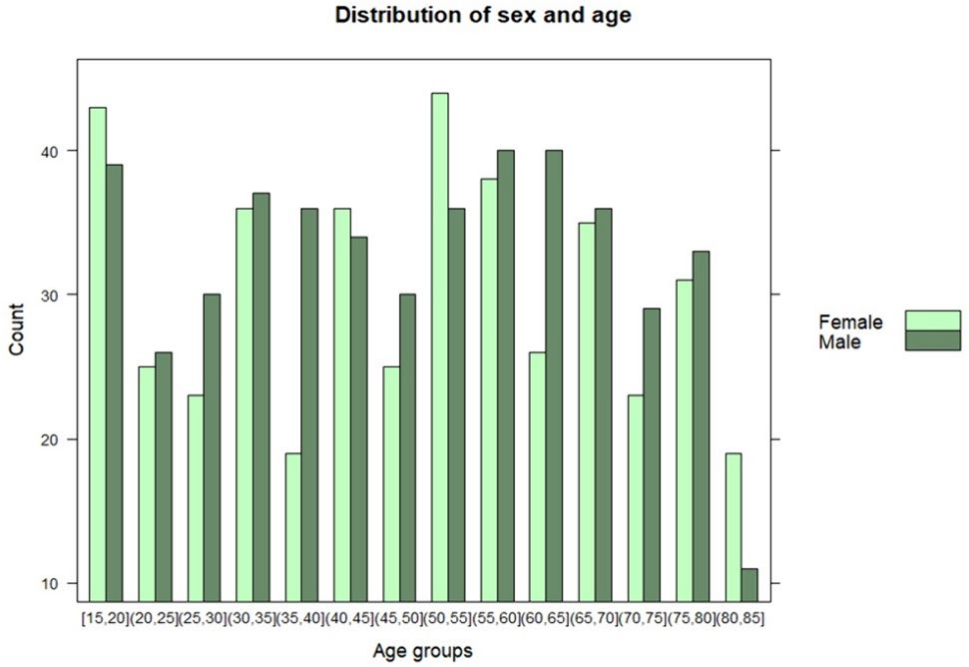
Age distribution of study cohort by sex. Light green bars = female, Dark green bars = male

A total of 1041 subjects were included in the study. Of these 1041 subjects, 41 individuals reported having diabetes and were thus ineligible to participate; seven individuals were identified with HbA1c ≥ 48 mmol/mol and were excluded due to the risk of having diabetes; two individuals reported being possibly pregnant and were therefore excluded; 31 individuals were excluded due to self-reported CVD (7 subjects had atrial fibrillation, 1 had a pacemaker and 29 had ischaemic heart disease); 28 individuals were excluded to self-reported cancer; 12 individuals were excluded because of thyroid dysregulation; and 39 individuals were ineligible due to their use of medication influencing the heart rate. The final study population therefore comprised 875 subjects, among whom 48% (*N* = 421) were females, and the median age was 50 (interquartile range [IQR] 32–65) years. The age distribution of the study cohort by sex is presented in Fig. 1. The total study population had a median BMI of 26 (IQR 24–29) kg/m^2^.

Smokers and former smokers accounted for 19% (*N* = 155) and 30% (*N* = 242), respectively, of the total study cohort. Approximately 33% (*N* = 252) categorised their physical activity level within the last year as vigorous/moderate and 67% (*N* = 508) as light/sedentary. Nine subjects (1.3%) reported a daily alcohol consumption of ≥ 5 units. Approximately 7% (*N* = 49) consumed ≥ 5 units per week. Further details on the characteristics of the study cohort are given in Table 1.

**Table 1:** Characteristics of the study population

Characteristics of study population	Pooled		Females		Males	
	*N*	Values	*N*	Values	*N*	Values
*Clinical*						
*N* (%)	875	100	421	48%	454	52%
Age (years)	875	50 [32.1–65]	421	50 [32.6–65]	454	49 [31.6–65.0]
BMI (kg/m^2^)	823	26.0 [23.5–29.3]	395	25.5 [23.0–29.5]	428	26.4 [24.0–29.1]
BP systolic (mmHg)	874	126 [116;138]	421	119 [112;135]	454	128 [119;140]
BP diastolic (mmHg)	874	77 [72;83]	420	76 [72;82]	454	77 [72;84]
Smoking	804		389		415	
- Never smoker		406 (50%)		213 (55%)		193 (47%)
Current smoker		155 (19%)		75 (19%)		81 (20%)
- Former smoker		242 (30%)		101 (26%)		141 (34%)
Physical activity level^a^	760		368		392	
Vigorous		32 (4.2%)		12 (3.3%)		20 (5.1%)
Moderate		220 (29%)		91 (25%)		129 (33%)
- Light		427 (56%)		222 (60%)		205 (52%)
Sedentary		81 (11%)		43 (12%)		38 (9.7%)
Frequency of ≥ 5 units of alcohol	695		324		372	
Daily		9 (1.3%)		2 (0.6%)		7 (1.9%)
Weekly		49 (7.1%)		13 (4.0%)		36 (9.7%)
Monthly		108 (16%)		41 (13%)		67 (18%)
Never/rarely		529 (76%)		267 (82%)		262 (70%)
Caffeine consumed before examination	871	465 (53%)	418	223 (53%)	453	244 (53%)
Physical activity before test	871	97 (11%)	418	45 (11%)	453	52 (11%)
Meal before examination	871	531 (60%)	418	264 (63%)	453	266 (58%)
Examination before 12 p.m.	875	244 (28%)	421	118 (28%)	454	126 (28%)
*Biochemical*						
HbA1c (mmol/mol)	852	35 [32–37]	409	35 [32–37]	443	34 [32–36]
UACR (mg/g)	626	10 [7–20]	293	15 [9–26]	333	8 [6–12]
Cholesterol (mmol/L)	854	4.90 [4.20–5.70]	411	5.00 [4.20–5.90]	443	4.80 [4.10–5.60]
HDL (mmol/L)	854	1.40 [1.20–1.70]	411	1.60 [1.30–1.80]	443	1.30 [1.10–1.50]
LDL (mmol/L)	840	2.80 [2.10–3.40]	408	2.80 [2.20–3.50]	432	2.80 [2.10–;3.33]
Triglycerides (mmol/L)	854	1.40 [1.00–2.00]	411	1.30 [0.90–1.80]	443	1.50 [1.10–2.30]
eGFR (ml/min/1.73 m^2^)	854	96 [84–110]	411	94 [84–108]	443	97 [84–111]

Data in table are given as the median with the interquartile range (IQR) in square brackets or as the proportions, with the percentage (%) in parentheses

*BP* Blood pressure,* BMI* basal metabolic rate, *eGFR* estimated glomerular filtration rate,* HbA1c* haemoglobin A1c, *HDL* high-density lipoprotein cholesterol, *LDL* Low-density lipoprotein cholesterol, *UACR* urine albumin-to-creatinine ratio

^a^The level of physical activity during the last year

### Missing data

A total of 136 observations were missing in the outcomes of the lying-to-standing test. Of these, 112 observations were excluded due to data errors or incorrect exercise performance, and the remaining 24 observations were missing for unknown reasons.

The results of the deep breathing test of 30 subjects were unavailable either due to data errors or their inability to perform the test.

Of the 875 subjects, 224 were unable to successfully complete the Valsalva manoeuvre (Table 2).

**Table 2:** Distribution of cardiovascular autonomic function measures

CAN measures	Pooled		Females		Males	
	*N*	875	*N*	421	*N*	454
*CARTs*	875		421		454	
Lying-to-standing (ratio)	739	1.21 [1.12–1.38]	349	1.20 [1.12–1.35]	390	1.21 [1.13–1.41]
Deep breathing (ratio)	845	1.27 [1.16–1.42]	408	1.29 [1.18–1.43]	437	1.25 [1.14–1.41]
Valsalva manoeuvre (ratio)	651	1.55 [1.38–1.74]	282	1.56 [1.39–1.79]	369	1.55 [1.37–1.72]
*HRV measures*						
Heart rate (bpm)	843	67 [61–74]	409	68 [62–75]	434	66 [60–73]
SDNN (ms)	839	37 [27–54]	409	37 [26–54]	430	38 [27–54]
RMSSD (ms)	839	27 [17–44]	409	28 [17–45]	430	26 [16–42]
LF power (ms^2^)	839	152 [67–368]	409	137 [60–334]	430	175 [75–399]
HF power (ms^2^)	839	100 [38–263]	409	115 [46–270]	430	87 [29–243]
Total power (ms^2^)	839	486 [235–1,033]	409	468 [229–1,027]	430	519 [252–1,061]

Data are given in medians (IQR)

*CAN* Cardiovascular autonomic neuropathy, *CARTs* Cardiovascular autonomic reflex tests, *HRV* Heart rate variability,* IQR* interquartile range, *LF/HF* Low frequency/ high frequency, *RMSSD* root mean square of the sum of the squares of differences between consecutive R–R intervals, *SDNN* standard deviation of normal-to-normal intervals

There were > 5% missing observations for the lying-to-standing test and for the Valsalva manoeuvre. The missingness in the outcome of the lying-to-standing test was significantly associated with higher age (ESM Appendix section 2), indicating that the likelihood of missing data for this outcome increased with higher age. For the Valsalva manoeuvre, missingness significantly increased with higher age as well as in females, suggesting that especially elderly and females had difficulties in completing the tests. A visualisation of the estimated regression models can be seen in ESM Appendix section 2.

### Distribution of the outcomes

The median lying-to-standing ratio was 1.21 (IQR 1.12–1.38), the median deep breathing ratio was 1.27 (IQR 1.16–1.42) and the median Valsalva manoeuvre was 1.55 (IQR 1.38–1.74) (Table 2). Distribution of the HRV indices and LF and HF power for the CARTs are shown in Table 2 and ESM Appendix Table 1 (Appendix section 1.1.2).

### Age-specific final models

**Fig. 2:**
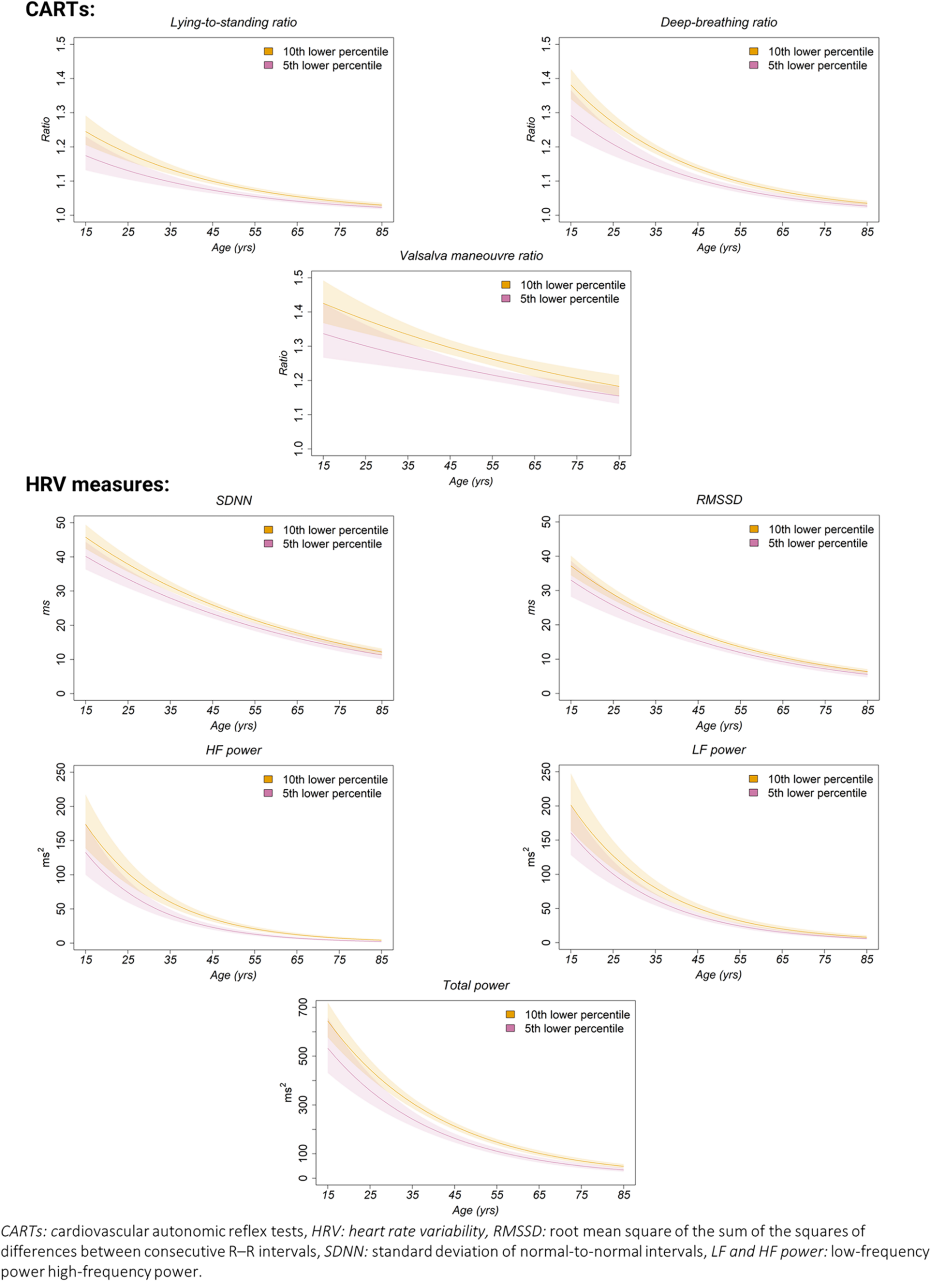
Estimated normative thresholds of cardiovascular autonomic function at the 5th (purple) and 10th (orange) lower percentile in subjects without diabetes and known cardiovascular disease. HRV measures are presented for the median heart rate 67 bpm. The outcome measures were log-transformed before the analysis. The estimates displayed along the* y*-axis have been back-transformed to the original scale. *CARTs* Cardiovascular autonomic reflex tests, *HRV* heart rate variability, *RMSSD* root mean square of the sum of the squares of differences between consecutive R–R intervals, *SDNN* standard deviation of normal-to-normal intervals

Higher age was statistically significantly and inversely associated with all outcomes of CARTs and HRV (Fig. 2; ESM Appendix section 3). The log transformation of the outcomes allowed a gradual decline in the generated trajectories at the lowest 5th and 10th percentiles with increasing age. For LF and HF power during the 5-min resting state and the reflex tests, the declines levelled off and assumed a horizontal trend above the age of approximately 55 years (Fig. 2; ESM Appendix section 3).

All associations between age and the outcomes retained statistical significance after correcting for multiple tests.

#### Age-specific estimates

The estimated normative reference values for CARTs derived for 5-year intervals are presented in Table 3. The age- and heart rate-specific reference values for HRV indices are presented in ESM Appendix section 3.2–3.

**Table 3:** Age-specific normative thresholds for the cardiovascular autonomic reflex tests, estimated at the lower 5th percentile

Age range group (years)	Lying-to-standing ratio (*N* = 739)^a^		Deep-breathing ratio (*N* = 845)^a^		Valsalva manoeuvre ratio (*N* = 651)^a^	
	*N*	Estimate (95% CI)	*N*	Estimate (95% CI)	*N*	Estimate (95% CI)
15–19	68	1.16 (1.12–1.21)	78	1.27 (1.22–1.33)	59	1.33 (1.26–1.41)
20–24	47	1.14 (1.11–1.18)	50	1.23 (1.19–1.27)	36	1.31 (1.25–1.38)
25–29	51	1.12 (1.10–1.15)	51	1.19 (1.16–1.23)	45	1.29 (1.25–1.35)
30–34	64	1.11 (1.09–1.13)	72	1.16 (1.14–1.19)	58	1.28 (1.24–1.32)
35–39	47	1.09 (1.08–1.11)	55	1.14 (1.12–1.15)	51	1.26 (1.23–1.30)
40–44	58	1.08 (1.07–1.09)	66	1.11 (1.10–1.13)	56	1.25 (1.22–1.28)
45–49	48	1.07 (1.06–1.08)	53	1.10 (1.09–1.11)	46	1.23 (1.21–1.26)
50–54	62	1.06 (1.05–1.07)	78	1.08 (1.07–1.09)	71	1.22 (1.20–1.24)
55–59	62	1.05 (1.04–1.06)	75	1.07 (1.06–1.08)	56	1.21 (1.19–1.23)
60–64	59	1.04 (1.04–1.05)	66	1.06 (1.05–1.07)	50	1.20 (1.18–1.22)
65–69	60	1.04 (1.03–1.05)	69	1.05 (1.04–1.06)	49	1.19 (1.17–1.21)
70–74	38	1.03 (1.03–1.04)	46	1.04 (1.03–1.05)	32	1.18 (1.16–1.20)
75–79	56	1.03 (1.02–1.04)	61	1.03 (1.03–1.04)	31	1.17 (1.15–1.19)
80–85	19	1.02 (1.02–1.03)	25	1.03 (1.02–1.04)	11	1.16 (1.14–1.19)

Results from log-transformed models. Estimates and confidence intervals (CIs) are back-transformed. The results are estimated from the median age of the specific age spans and are presented as ratios with 95% CI

^a^Total number of subjects performing the respective test

 The outcome specific model formulas for the CARTs and HRV thresholds estimated at the lowest 10th percentile are given in ESM Appendix section 6.1, and the formulas for both CARTs and HRV thresholds for females and males, respectively, are given in ESM Appendix section 6.2. Outcome-specific model formulas for the CARTs and HRV thresholds estimated at the lowest 5th percentile are as follows:

CARTs:

$$Lying - to - standing\,\,threshold_{{5th\,percentile}} = exp\left( { - 1.31 - 0.029*age} \right) + 1$$

$$Deep - breathing\,\,threshold_{{5th\,percentile}} = exp\left( { - 0.72 - 0.034*age} \right) + 1$$

$$Valsalva\,\,maneouvre\,\,threshold_{{5th\,percentile}} = exp\left( { - 0.92 - 0.011*age} \right) + 1$$

HRV:

$$SDNN\,\,threshold_{{5th\,percentile}} = exp\left( {5.43 - 0.018*age - 0.022*heart\,\,rate} \right)$$

$$RMSSD\,\,threshold_{{5th\,percentile}} = exp\left( {6.46 - 0.025*age - 0.039*heart\,\,rate} \right)$$

$$HFpower\,\,threshold_{{5th\,percentile}} = exp\left( {10.21 - 0.058*age - 0.066*hear\,\,trate} \right)$$

$$LF\,\,power\,\,threshold_{{5th\,percentile}} = exp\left( {7.86 - 0.047*age - 0.031*heart\,\,rate} \right)$$

$$Total\,\,power\,\,threshold_{{5th\,percentile}} = exp\left( {9.33 - 0.039*age - 0.037*heart\,\,rate} \right)$$

### Sex-stratified models

Being a male compared to being a female was significantly associated with a 34% lower level of deep breathing (95% CI 22–44%; *P* < 0.001), a 16% lower level of RMSSD (95% CI 6–25%; *P* < 0.002), a 54% lower level of HF power during the 5-min supine recording (95% CI 41%–64%; *P* < 0.001), a 49% lower level of HF power during lying-to-standing (95% CI 21–67%; *P* = 0.003), a 29% lower level of LF power during deep breathing (95% CI 11–44%; *P* = 0.003) and a 46% lower level of HF power during the Valsalva manoeuvre (95% CI 19–64%; *P* = 0.004). All associations held significance after correcting for multiple testing.

Sex-stratified models for all outcomes are shown in ESM Appendix section 5. The data distributions of all outcomes (CARTs and HRV measures) between sexes and age groups. are visualised in ESM Appendix section 1.2. 

### Effect of pre-test exposures

#### Physical activity

Among the 875 individuals participating in the study, 97 (11%) reported being physically active on the day of the examination. Being physically active on the day of the examination was significantly associated with a decrease of 3% (95% CI 0.4–6.4%; *P* = 0.025) in the lying-to-standing ratio and a decrease of 4% (95% CI 0.5–8%, *P* = 0.025) in the Valsalva manoeuvre ratio. Following correction for multiple testing, the identified associations were no longer statistically significant.

#### Meals and caffeine intake

In total, 531 (60%) of the participants reported having consumed a meal within 3 h before the examination, while 465 (53%) had consumed caffeine within 1 h before the tests. No significant associations between these factors and cardiovascular autonomic function were observed (ESM Appendix section 1.1.3).

#### Time of examination

Examinations for 244 (28%) participants occurred before noon (12 p.m.). Examinations conducted before noon (12 p.m.) were significantly associated with a 19% (95% CI 1–40%; *P* = 0.03) increase in resting HF power, an 18% (95% CI 1–39%; *P* = 0.04) increase in LF power during the Valsalva manoeuvre and a 23% (95% CI 2–48%; *P* = 0.03) increase in HF power during the Valsalva manoeuvre. However, none of the observed associations retained significance after adjusting for multiple tests (ESM Appendix section 1.1.3).

## Discussion

In this population-based study, we present normative threshold values for the internationally recommended CARTs to be used in the diagnosis of CAN. In addition, we present normative threshold values for HRV indices traditionally used in autonomic research, namely the time-domain measures SDNN and RMSSD and the frequency-domain metrics LF, HF and total frequency power. As novel outcomes, we have estimated threshold values for LF and HF power during the three CARTs (ESM Appendix section 6.1.3) which may enable a more reliable assessment of the HRV, as these are measured under provocation testing where measures may be more standardised compared to the resting state. We provide both tables with age- and heart rate-specific thresholds and equations that can be used to calculate a more precise threshold. The study is to our knowledge the largest of its kind in terms of both quantity of participants and range of age, including 875 participants (48% females) aged 15–85 years. Previous studies have presented normative thresholds for CARTs that were often based on data from smaller cohorts [14, 16]. Our estimates of the CARTs are within the ranges of estimates from other studies; notably, our values are in the lower part of these ranges. To our knowledge, only a few studies have established normative thresholds for HRV indices [14, 32]. One of these latter studies [14] provides normative thresholds for both CARTs and HRV indices, including RMSSD, LF power and HF power, based on data from 120 healthy individuals, with thresholds estimated at the 2.3rd lowest percentile. The RMSSD threshold in our study is higher than this previously reported value. The normative thresholds for LF and HF power are not directly comparable with those of the previous study [14] due to differences in units. However, the median LF and HF power values from our study closely resemble the mean LF and HF power reported in another study of 286 healthy persons in a general population [32]. Threshold values of LF and HF power during the three CARTs have not previously been established.

### Effect of age

We found statistically significantly lower levels of both CART outcomes and HRV indices with increasing age. It is well-established that age-dependent decreases in both CART outcomes and HRV indices are present in healthy individuals [14, 15, 17–19]. In our study, a logarithmic relationship between age and these indices provided the best fit, ensuring that our quantile predictions remained within biologically plausible ranges, namely > 1 for CART outcomes and > 0 for HRV indices. The logarithmic relationship has been observed previously [14, 16] and suggests a gradual tapering of cardiovascular autonomic nerve function, which may be physiologically plausible. The HRV indices HF power and LF power appeared to plateau around the age of 55 years (Fig. 2; ESM Appendix section 3.2).

A plateau phase for some CAN outcomes has only been demonstrated in a few studies [17, 33]. In a previously published study, we reported normative data on CARTs and HRV indices in a Greenlandic Inuit population [17]. In that study, the outcomes of the lying-to-standing test plateaued at the age of 60 years. Whether these plateaus represent a natural physiological phenomenon or are the result of unknown confounding factor(s) remains to be elucidated. It is possible that the plateau phenomena could be a result of healthy survivor bias or recruitment bias in the sense that participants in older age groups could be particularly healthy compared to non-responders and therefore could exhibit preserved autonomic function with no drop in autonomic measures with increasing age. In addition, after exclusion, there was fewer participants in the older age groups, thus increasing the risk of outlier effects on estimates.

### Effect of sex

We demonstrated significantly higher values in females for six autonomic measures: the deep-breathing ratio, RMSSD, HF power, HF power during the lying-to-standing test, LF power during the deep breathing test and HF power during the Valsalva manoeuvre. These higher values mainly indicate that females may have a higher parasympathetic drive compared to males. These findings are in line with those of a small number of earlier studies on the topic showing sex differences in autonomic function, with males possibly showing a higher sympathetic drive compared to women [20, 34, 35]. This difference may be particularly significant between sexes when women are premenopausal, suggesting that oestrogen may have a modulating effect on the sympathetic and parasympathetic balance by enhancing the parasympathetic activity [36]. Previously, HF power has been shown to be similar in males and females, while LF power tends to be lower in females [20]. However, these findings were based on a relatively narrow age range of the participants (age 45–65 years) and may not be comparable to those for our population. Taken together, our findings confirm that autonomic measures may differ between sexes.

We lack an understanding of the underlying factors contributing to the potential disparity in autonomic function between the sexes. Males may have a higher prevalence of CVD risk factors, and if it is this higher prevalence that causes males’ normal threshold value to be lower than that of females, there is a risk of incorrectly categorising a greater number of males as healthy when the normal range is applied to a sick population. Thus, here we present normative thresholds of cardiovascular autonomic function for the entire study population, including both males and females. However, to account for potential differences between the sexes, sex-specific normative thresholds for all outcomes are presented in the ESM Appendix section 6.2.

### Effect of pre-test exposures

#### Physical activity

Strenuous physical activity on the day of the examination was associated with marginally but statistically significantly lower outcomes for the lying-to-standing test and Valsalva manoeuvre. However, none of the associations retained significance after adjusting for multiple tests, suggesting that the initial significant associations observed might have been due to random chance. Thus, it would appear to be of little importance to exclude people from autonomic testing if they have exercised before testing.

#### Meals and caffeine intake

Intake of larger meals and caffeine within 3 h before autonomic testing did not affect the outcomes. Thus, placing restrictions on these parameters may not be of importance in people without diseases capable of inducing metabolic changes that could affect autonomic function. However, in individuals with metabolic diseases, food intake could have a more pronounced effect on autonomic function. One example are people with diabetes, who are prone to glucose excursions after meals; these changes in blood glucose levels have been shown to affect CAN measures. Therefore, it is recommended not to test people with diabetes within 2 h after a meal and in the presence of marked hypoglycaemia or hyperglycaemia [37]. In our study, caffeine intake did not affect outcomes, indicating that the exclusion of people who have ingested caffeine before testing may not be strictly necessary. However, it is still recommended in the literature to have people refrain from caffeine at least 2 h before testing [37].

#### Time of examination

No differences in CART outcomes measured before and after noon were noted, suggesting that the timing of tests performed to diagnose CAN has little importance. Nevertheless, HF power was significantly higher before noon, indicating a higher parasympathetic tone in this time slot. This result suggests that the timing of HRV indices could be taken into account when designing studies including these measures. However, this association lost significance after adjusting for multiple tests, which may infer that the initial finding could have occurred by chance.

### Strengths and limitations

This study is based on a large population free of diabetes, CVD and drugs that potentially could confound certain autonomic measures. The inclusion of individuals covering a wide age range allows for representative and reliable normative data.

It is a general limitation that population-based normative thresholds do not take the underlying CAN risk factors among the background population into account. The residents of Lolland and Falster generally exhibit a low socio-economic status, a higher occurrence of physical and mental illnesses and a reduced life expectancy in comparison to the broader Danish population [21]. These factors may have influenced cardiovascular autonomic function and the estimated thresholds, causing them not to accurately represent the true cut-off between normal and abnormal cardiovascular autonomic function.

Moreover, the Danish population primarily consists of Caucasians, who may exhibit genetic or environmental characteristics that can have influenced the normative thresholds. This aspect should be taken into account when applying these thresholds to diverse ethnicities.

The algorithm of identifying R-R intervals may differ from the device used in the present study and other devices. Thus, the applicability of the presented threshold estimates may rely on the method of R peak identification used.

## Conclusions

We present age-specific normative reference thresholds for CARTs and age and sex-specific normative thresholds for HRV indices based on a large Danish cohort. We provide both reference tables and equations that can be used to assess CAN in clinical and research settings. We observed no significant impact of pre-test conditions on either CARTs or HRV measures and, therefore, the presence of these factors may not lead to exclusion from autonomic testing. While our findings suggest that females exhibit a higher drive of parasympathetic activity, further studies are needed to uncover the underlying mechanisms.

## Supplementary Information

Below is the link to the electronic supplementary material.

Supplementary file1 (DOCX 2148 KB)

## Data Availability

The datasets generated during and/or analysed during the current study are not publicly available due to restrictions by the Danish Data Protection Agency but are available from the corresponding author upon reasonable request and permission from LOFUS.

## Abbreviations

BMI: Body mass index
CAN: Cardiovascular autonomic neuropathy
CARTs: Cardiovascular autonomic reflex tests
CVD: Cardiovascular disease
HF: High frequency
HRV: Heart rate variability
IQR: Interquartile range
LF: Low frequency
LOFUS: The Lolland-Falster Health Study
RMSSD: Root mean square of the sum of the squares of differences between consecutive R–R intervals
SDNN: Standard deviation of normal-to-normal intervals
TSH: Thyroid stimulating hormone
