# Supplementary material for: Normative data on measures of cardiovascular autonomic neuropathy and the effect of pretest conditions in a large Danish non-diabetic CVD-free population from the Lolland-Falster Health Study
Source: Clin Auton Res. 2024 Oct 17;35(1):101–13. doi: 10.1007/s10286-024-01069-6 (PMC11937105; doi:10.1007/s10286-024-01069-6)
Supplement: Supplementary file 1 — Supplementary file1 (DOCX 2148 KB) [file 10286_2024_1069_MOESM1_ESM.docx]

Appendix for NORMATIVE DATA ON MEASURES OF CARDIOVASCULAR AUTONOMIC NEUROPATHY AND THE EFFECT OF PRETEST CONDITIONS IN A LARGE DANISH NON-DIABETIC CVD-FREE POPULATION

**Christian S Hansen*^1^, Marie Mathilde Bjerg Christensen*^2,3^; Dorte Vistisen^2,4^, Randi Jepsen^5^, Christina Ellervik^6,7,8^, Marit Eika Jørgensen^2,9,10^, Jesper Fleischer^11,12^.**

^1^Translational Type 2 Diabetes Research, Steno Diabetes Center Copenhagen, Herlev, Denmark

^2^Clinical Epidemiology Research, Steno Diabetes Center Copenhagen, Herlev, Denmark

^3^Department of Public Health, Aarhus University, Denmark

^4^Novo Nordisk A/S, Søborg, Denmark

^5^Lolland-Falster Health Study, Nykøbing F. Hospital, Region Zealand, Denmark

^6^Department of Clinical Medicine, University of Copenhagen, Copenhagen, Denmark

^7^Department of Clinical Biochemistry, Zealand University Hospital, Køge, Denmark

^8^Department of Laboratory Medicine, Boston Children’s Hospital, Harvard Medical School, Boston, MA, USA

^9^Steno Diabetes Center Greenland, Nuuk, Greenland

^10^National Institute of Public Health, University of Southern Denmark, Odense, Denmark

^11^Steno Diabetes Center Aarhus, Aarhus University Hospital, Aarhus, Denmark

^12^Steno Diabetes Center Zealand, Holbæk, Denmark

Table of content

[1 Introduction 5](#_Toc174452931)

[1.1 Packages, RStudio version 4.1.2 5](#_Toc174452932)

[1.2 Data distribution 6](#_Toc174452933)

[1.2.1 Distribution of age and sex 6](#_Toc174452934)

[1.2.2 Distribution of cardiovascular autonomic function measures over age 7](#_Toc174452935)

[1.2.3 Distribution of cardiovascular autonomic function measures by age group and sex 14](#_Toc174452936)

[1.2.4 Distribution of cardiovascular autonomic function measures: 21](#_Toc174452937)

[1.3 Influence of caffeine, food and exercise before examination on the outcomes 21](#_Toc174452938)

[2 Missing data 30](#_Toc174452939)

[3 Quantile regression models 32](#_Toc174452940)

[3.1 CARTs: 33](#_Toc174452941)

[3.1.1 Lying-to-standing: 33](#_Toc174452942)

[3.1.2 Deep breathing: 34](#_Toc174452943)

[3.1.3 Valsalva manoeuvre: 35](#_Toc174452944)

[3.2 HRV (adjusted for resting heart rate): 36](#_Toc174452945)

[3.2.1 SDNN: 36](#_Toc174452946)

[3.2.2 RMSSD: 37](#_Toc174452947)

[3.2.3 HF power: 38](#_Toc174452948)

[3.2.4 LF power: 39](#_Toc174452949)

[3.2.5 Total power: 40](#_Toc174452950)

[3.3 Frequency domain analyses during the CARTs: 41](#_Toc174452951)

[3.3.1 HF power during Lying to standing: 41](#_Toc174452952)

[3.3.2 LF power during Lying to standing: 41](#_Toc174452953)

[3.3.3 HF power during Deep breathing: 43](#_Toc174452954)

[3.3.4 LF power during Deep breathing: 44](#_Toc174452955)

[3.3.5 HF power during Valsalva manoeuvre: 45](#_Toc174452956)

[3.3.6 LF power during Valsalva manoeuvre: 46](#_Toc174452957)

[4 Adjustment for age and sex 46](#_Toc174452958)

[5 Final models stratified by sex 49](#_Toc174452959)

[5.1 Females 50](#_Toc174452960)

[CARTs 50](#_Toc174452961)

[5.1.1 Lying-to-standing: 50](#_Toc174452962)

[5.1.2 Deep breathing: 51](#_Toc174452963)

[5.1.3 Valsalva manoeuvre: 52](#_Toc174452964)

[HRV (adjusted for resting heart rate): 53](#_Toc174452965)

[5.1.4 SDNN: 53](#_Toc174452966)

[5.1.5 RMSSD: 54](#_Toc174452967)

[5.1.6 HF power: 55](#_Toc174452968)

[5.1.7 LF power: 56](#_Toc174452969)

[5.1.8 Total power: 57](#_Toc174452970)

[Frequency domain analyses during the CARTs: 58](#_Toc174452971)

[5.1.9 HF power during Lying-to-standing: 58](#_Toc174452972)

[5.1.10 LF power during Lying-to-standing: 59](#_Toc174452973)

[5.1.11 HF power during Deep-breathing: 60](#_Toc174452974)

[5.1.12 LF power during Deep-breathing: 61](#_Toc174452975)

[5.1.13 HF power during Valsalva manoeuvre: 62](#_Toc174452976)

[5.1.14 LF power during Valsalva manoeuvre: 63](#_Toc174452977)

[5.2 Males 64](#_Toc174452978)

[CARTs 64](#_Toc174452979)

[5.2.1 Lying-to-standing: 64](#_Toc174452980)

[5.2.2 Deep-breathing: 65](#_Toc174452981)

[5.2.3 Valsalva manoeuvre: 66](#_Toc174452982)

[HRV (adjusted for resting heart rate): 67](#_Toc174452983)

[5.2.4 SDNN: 67](#_Toc174452984)

[5.2.5 RMSSD: 68](#_Toc174452985)

[5.2.6 HF power: 69](#_Toc174452986)

[5.2.7 LF power: 70](#_Toc174452987)

[5.2.8 Total power: 71](#_Toc174452988)

[Frequency domain analyses during the CARTs: 72](#_Toc174452989)

[5.2.9 HF power during Lying-to-standing: 72](#_Toc174452990)

[5.2.10 LF power during Lying-to-standing: 73](#_Toc174452991)

[5.2.11 HF power during Deep-breathing: 74](#_Toc174452992)

[5.2.12 LF power during Deep-breathing: 75](#_Toc174452993)

[5.2.13 HF power during Valsalva manoeuvre: 76](#_Toc174452994)

[5.2.14 LF power during Valsalva manoeuvre: 77](#_Toc174452995)

[6 Formulas 77](#_Toc174452996)

[6.1 Total study population 77](#_Toc174452997)

[6.1.1 CARTs: 77](#_Toc174452998)

[6.1.2 HRV: 78](#_Toc174452999)

[6.1.3 Frequency domain analyses for the CARTs: 78](#_Toc174453000)

[6.2 Sex stratified study population 79](#_Toc174453001)

[Females: 79](#_Toc174453002)

[6.2.1 CARTs: 79](#_Toc174453003)

[6.2.2 HRV: 80](#_Toc174453004)

[6.2.3 Frequency domain analyses for the CARTs: 80](#_Toc174453005)

[Males 81](#_Toc174453006)

[6.2.4 CARTs: 81](#_Toc174453007)

[6.2.5 HRV: 81](#_Toc174453008)

[6.2.6 Frequency domain analyses for the CARTs: 82](#_Toc174453009)

[7 Tables 83](#_Toc174453010)

[7.1 CARTs 83](#_Toc174453011)

[7.2 SDNN 84](#_Toc174453012)

[7.3 RMSSD: 85](#_Toc174453013)

[7.4 HF power: 86](#_Toc174453014)

[7.5 LF power: 87](#_Toc174453015)

[7.6 Total power: 88](#_Toc174453016)

#

- 1. **Abbreviations:**

CAN – Cardiovascular autonomic neuropathy

CARTs – Cardiovascular autonomic reflex tests

HRV – Heart rate variability

bpm_hr - Heart rate

E:I – Deep-breathing ratio

RS – Lying-to-standing ratio

VM - Valsalva manoeuvre ratio

HR_SDNN - Standard deviation of normal-to-normal intervals, SDNN

HR_RMSSD - Root mean square of the sum of the squares of differences between consecutive R–R intervals, RMSSD

HR_LF - Low-frequency power, LF

HR_HF - High-frequency power, HF

HR_TOTAL - Total power

RS_HF – High-frequency power during the Lying-to-standing test

RS_LF - Low-frequency power during the Lying-to-standing test

EI_HF – High-frequency power during the Deep-breathing test

RS_LF - Low-frequency power during the Deep-breathing test

VM_HF – High-frequency power during the Valsalva manoeuvre test

VM_LF - Low-frequency power during the Valsalva manoeuvre test

# Introduction

We applied quantile regression to estimate the 5th and 10th percentile values of cardiovascular autonomic function outcomes in subjects without diabetes and known heart disease. Quantile regression extends the concept of linear regression and is suitable for establishing normative thresholds by fitting conditional quantiles/percentiles of the response using a general linear model that does not assume a specific distribution for the response. The regression coefficients are specific to the chosen percentile and do not apply to the entire range of data. This makes the method ideal for computing estimates of specific low or high percentiles, which is often the approach in normative threshold studies. Quantile regression offers greater flexibility compared to linear regression models as it requires fewer restrictive assumptions about normality.

We defined the normal threshold as the 5th percentile, based on the commonly accepted practice of a 5% false-positive rate in statistical testing. Additionally, we estimated the 10th percentile, which provides increased accuracy and precision but has lower specificity. Since there is no theory describing the adverse effects of exceeding high levels in heart variability measures, assessing an upper limit is not relevant.

To account for age-specific differences in the thresholds for each outcome, we included age as the exposure variable in the models. We applied a log transformation to the outcome measures of heart rate variability (HRV) to ensure that the percentile estimates remained positive (>0), as it is not biologically plausible for HRV measures to exhibit negative values. Similarly, for the cardiovascular autonomic reflex tests (CARTs), as the ratios cannot yield values between 0 and 1, we applied a log transformation to the ratio minus 1.

To address potential differences in autonomic nervous system functioning between males and females, we stratified our sample by sex and applied the same method described above. This approach was adopted to align with the assumption that variations in autonomic function may exist between the two sexes. Previous research has indicated that females, during the period after puberty and before menopause, tend to exhibit a lower sympathetic drive and a higher parasympathetic drive compared to males.

## Packages, RStudio version 4.1.2

The following packages were applied:

library("writexl")
library("excel.link")
library("dplyr")
library("ggplot2")
library("quantreg")
library("pander")
library("Epi")
library("tinytex")
library("flextable")
library("gtsummary")
library("tidyverse")
library("knitr")
library("kableExtra")
library("broom")
library("pandoc")

## Data distribution

### Distribution of age and sex

N=875


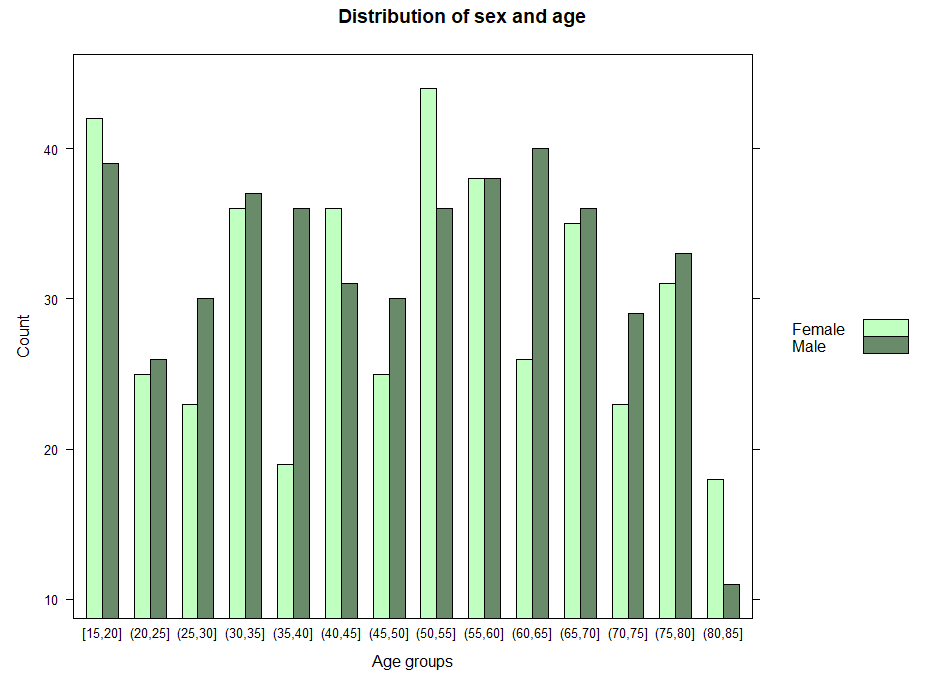


### Distribution of cardiovascular autonomic function measures over age


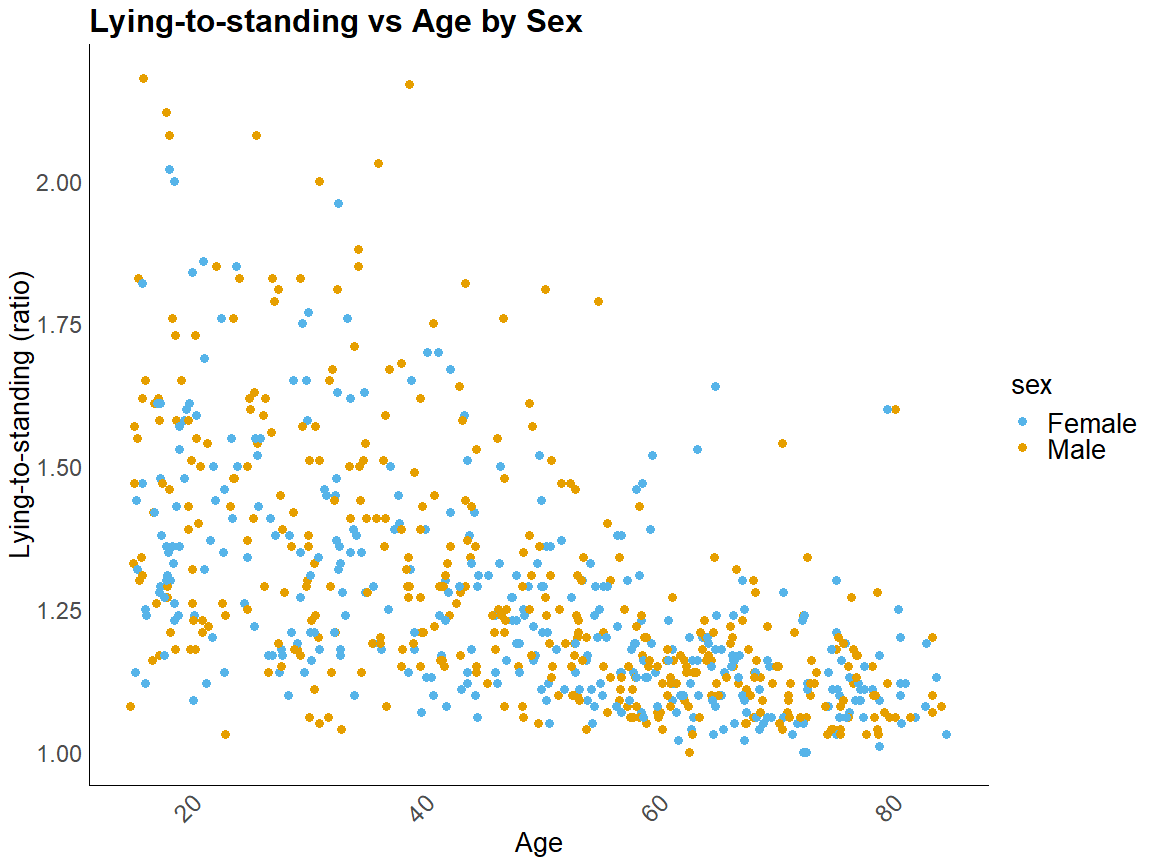

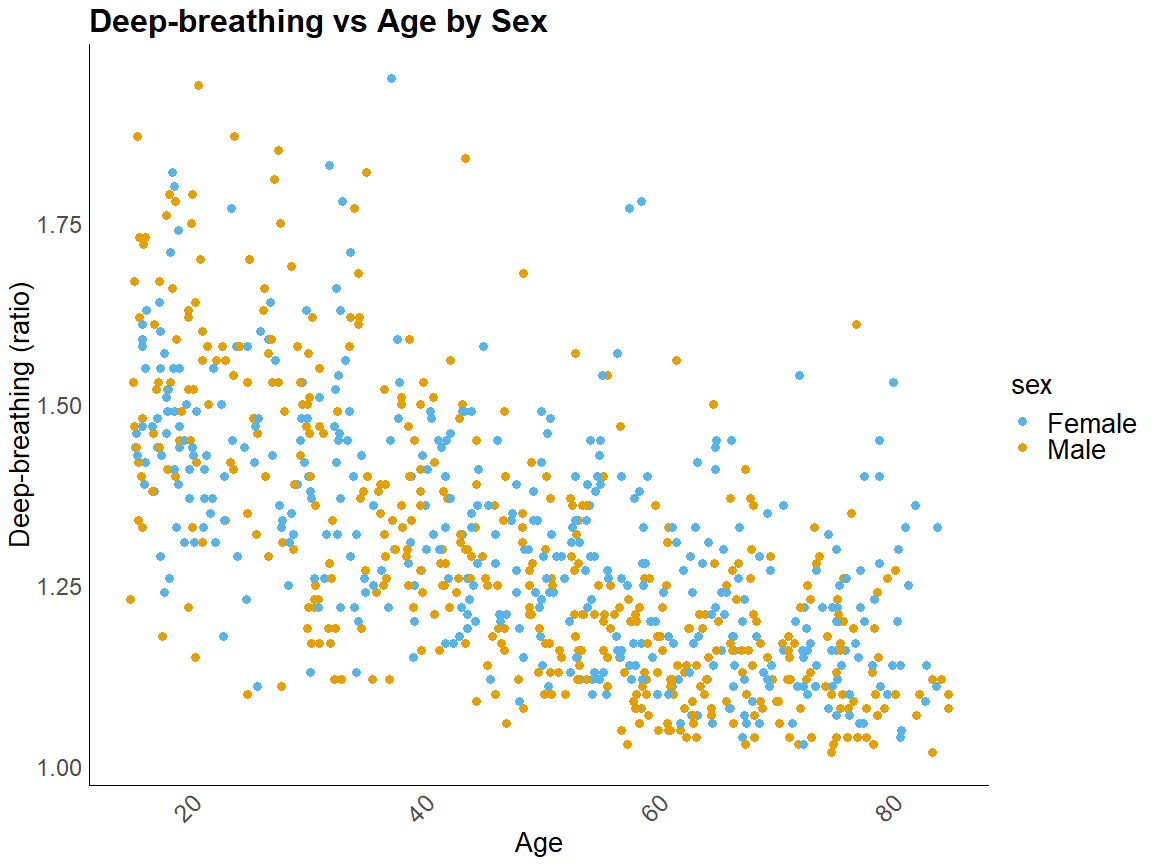

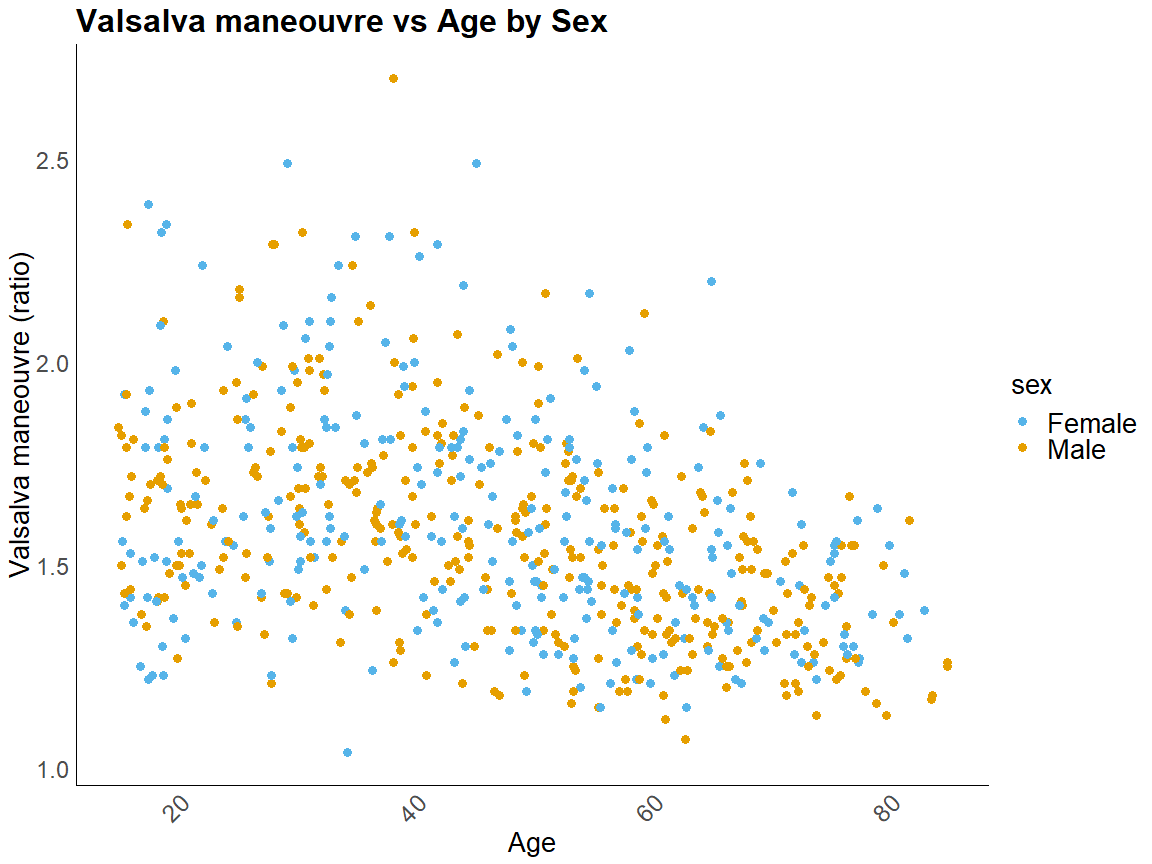

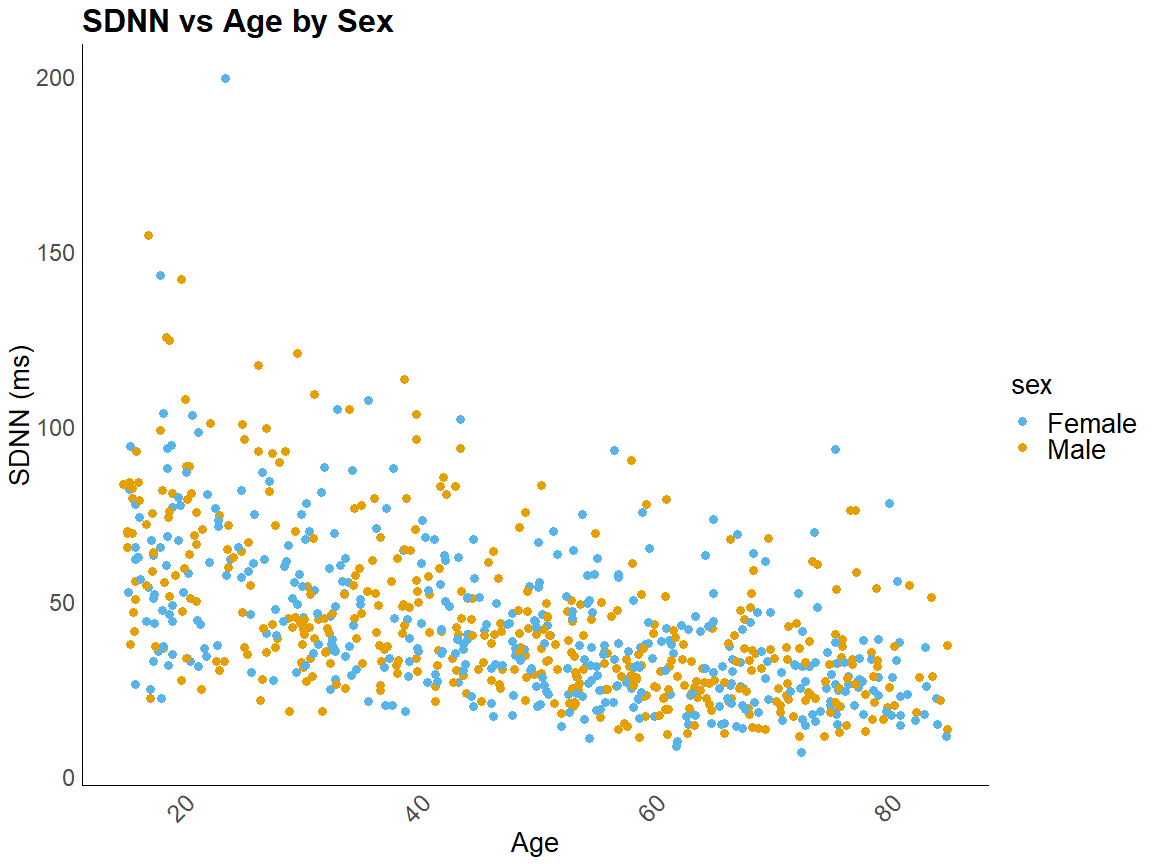

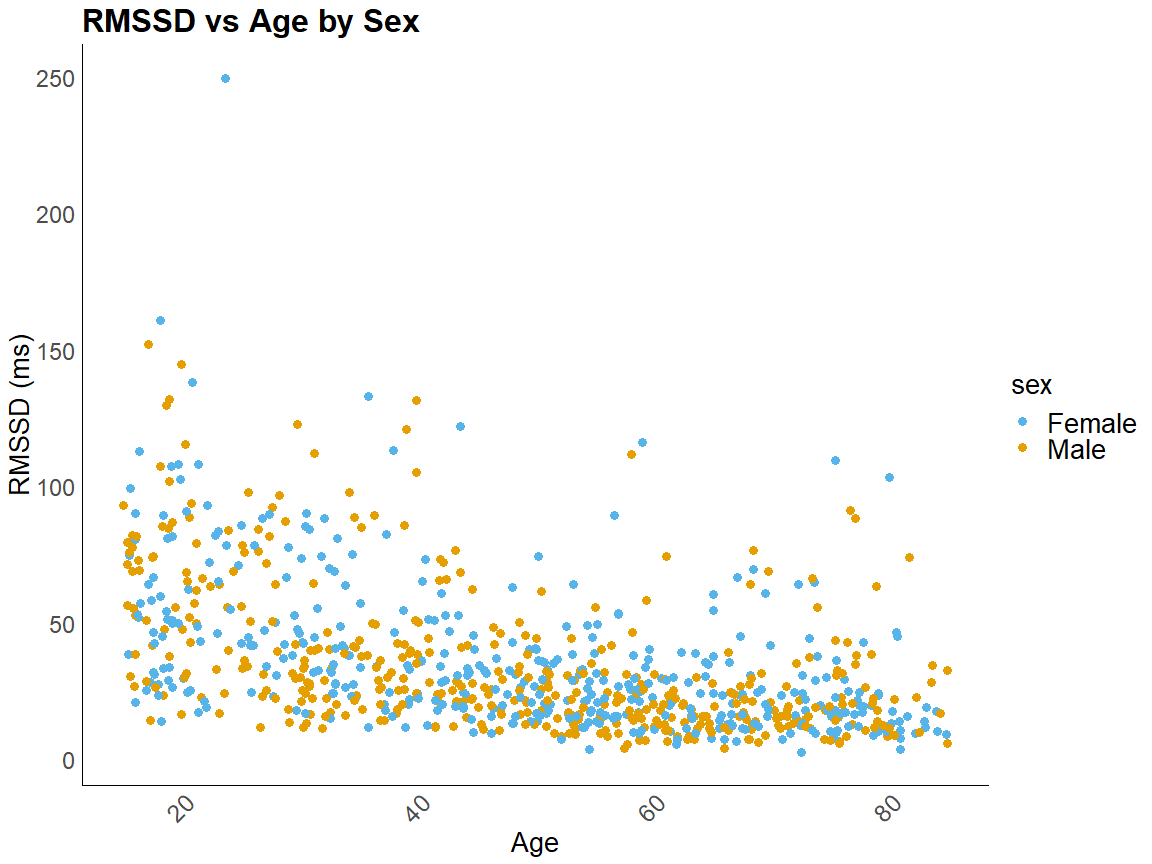

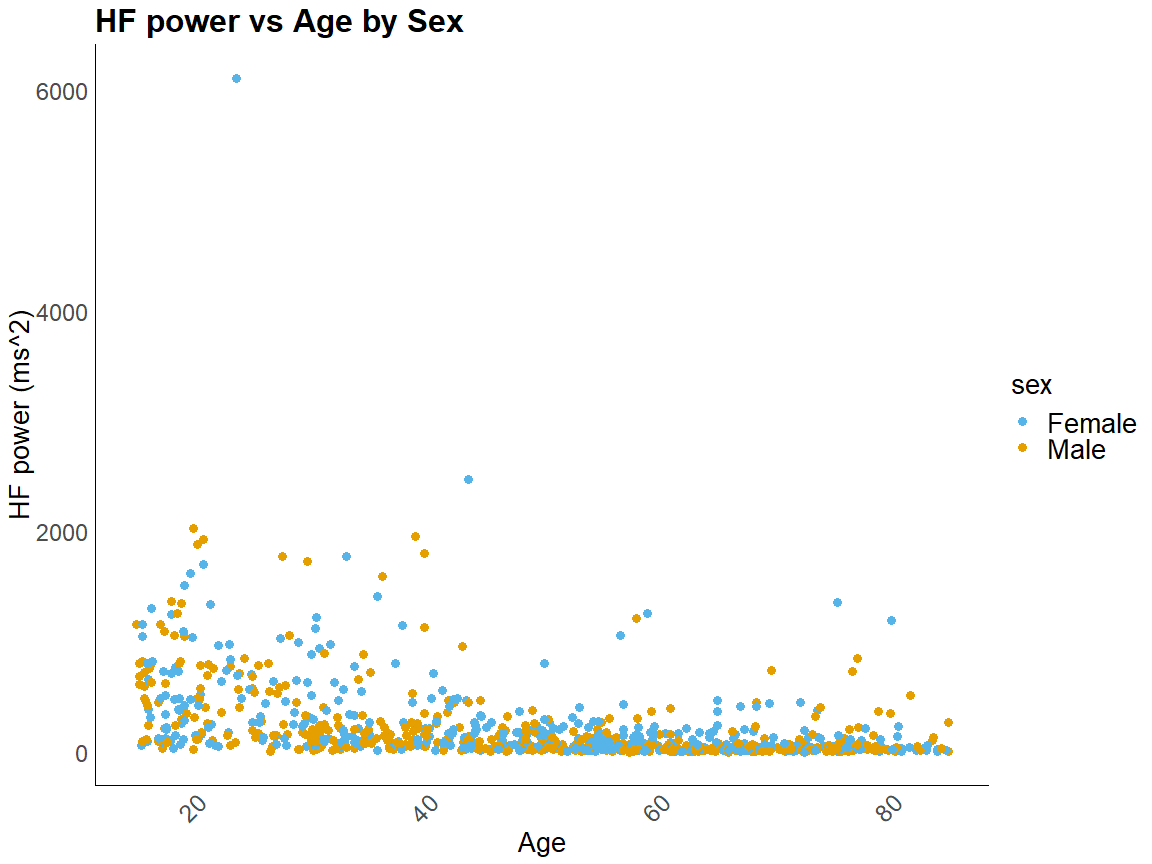

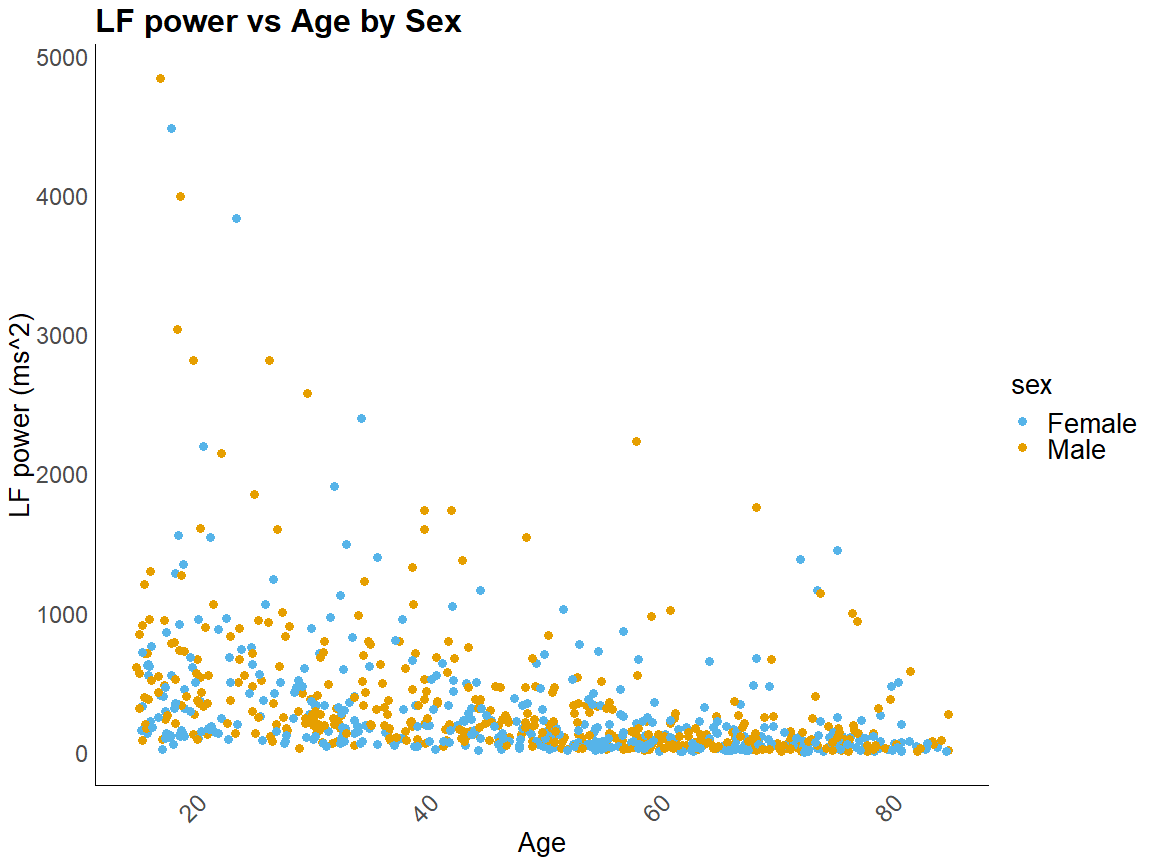

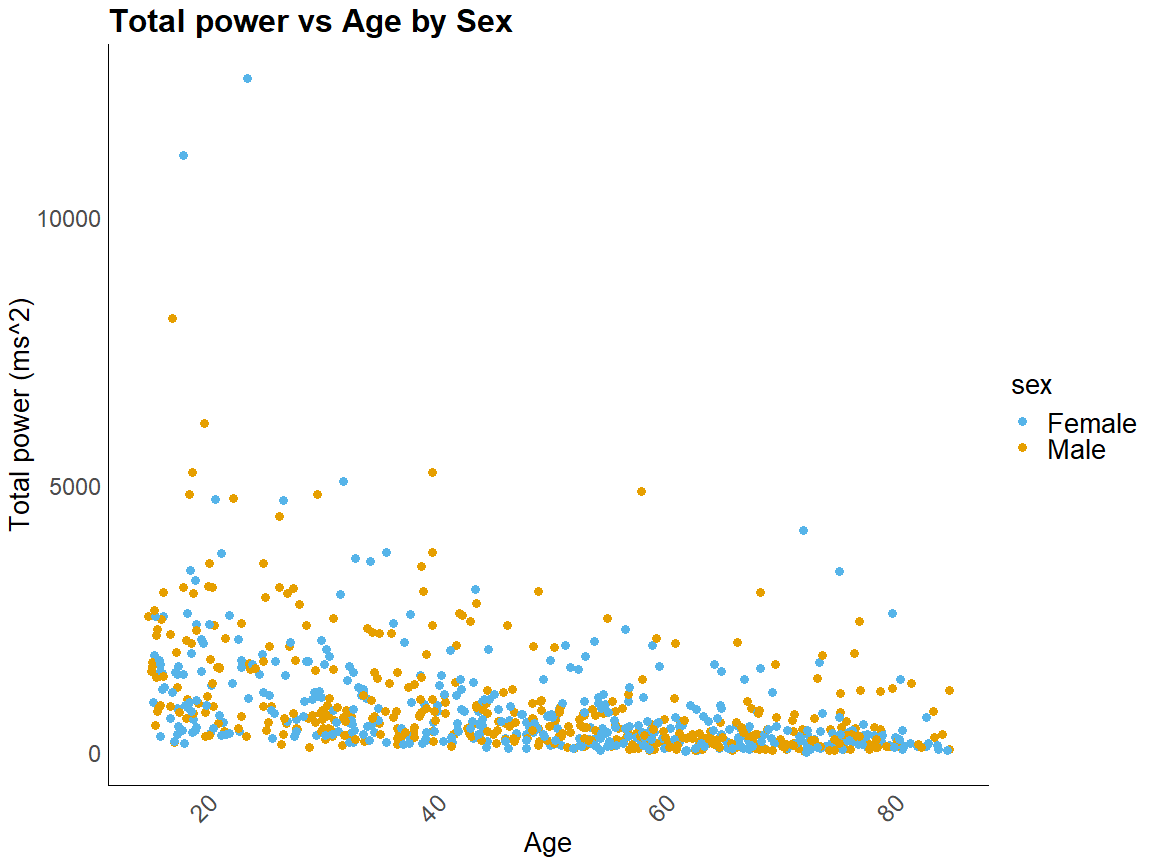

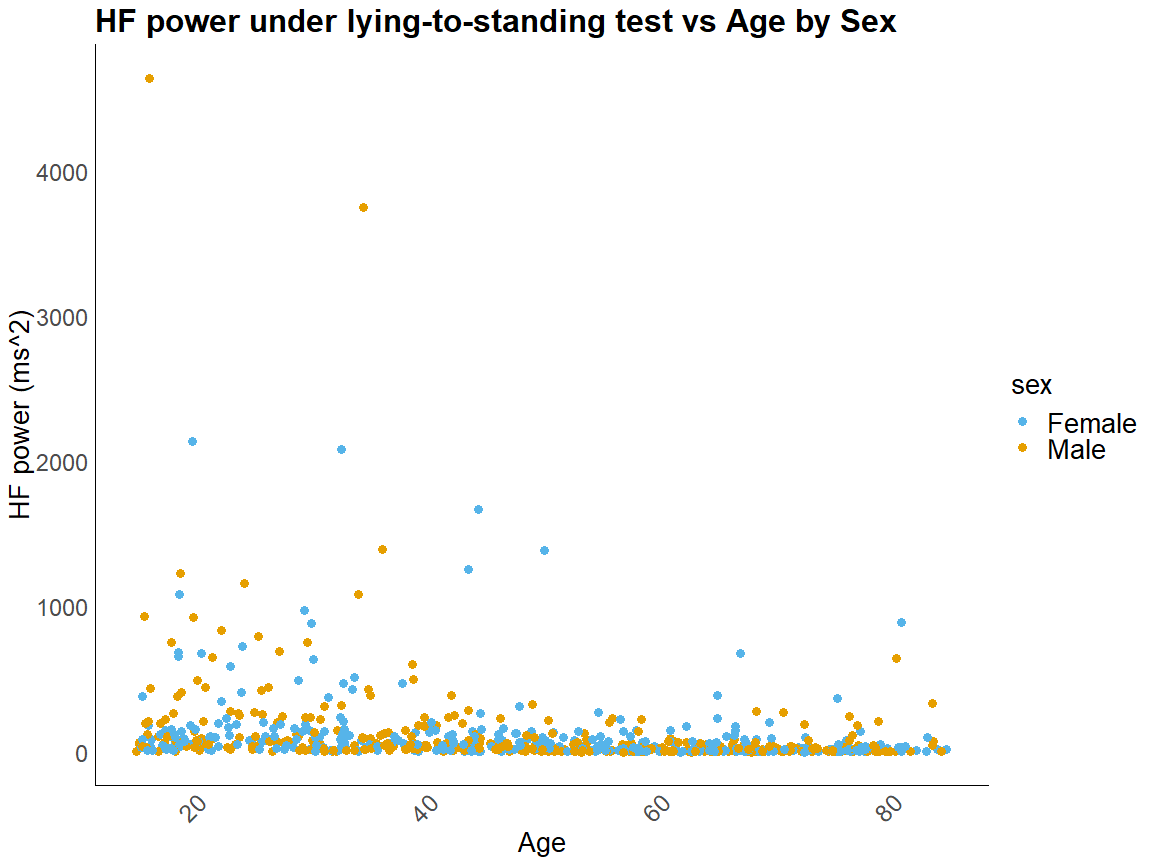

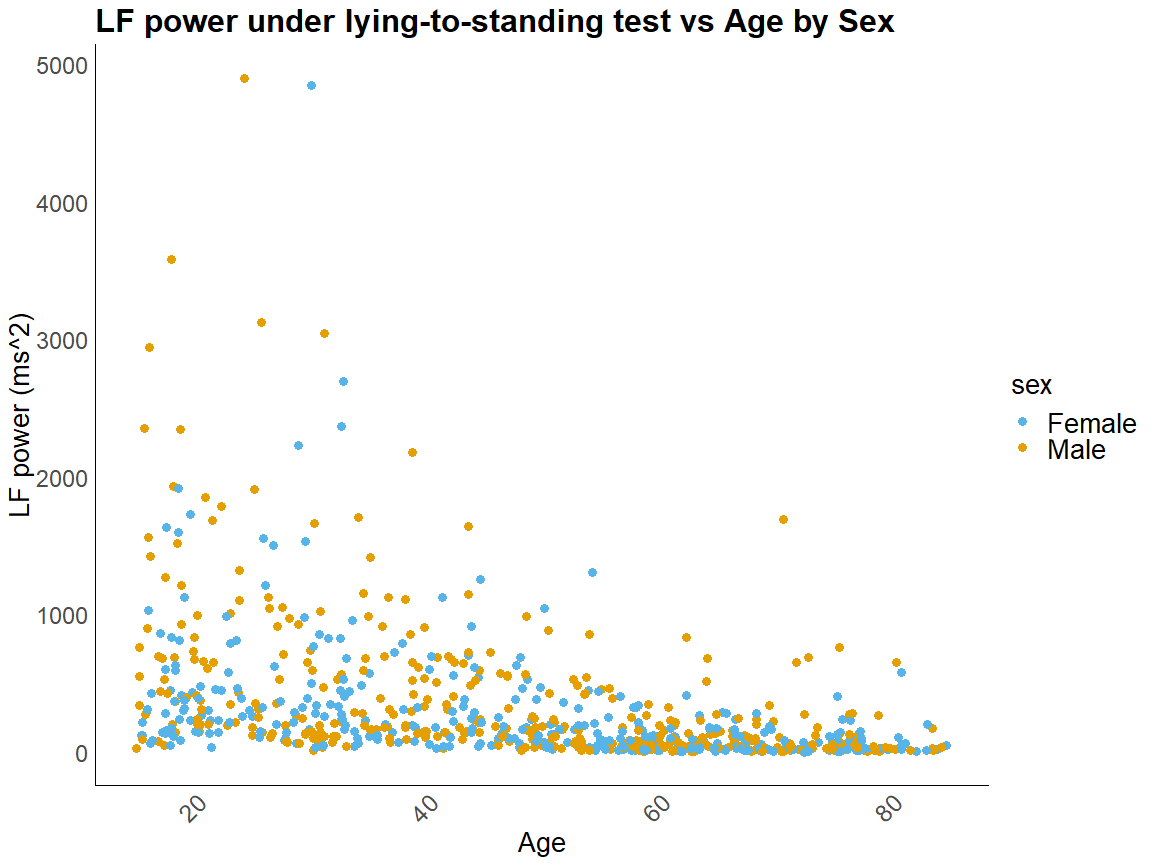

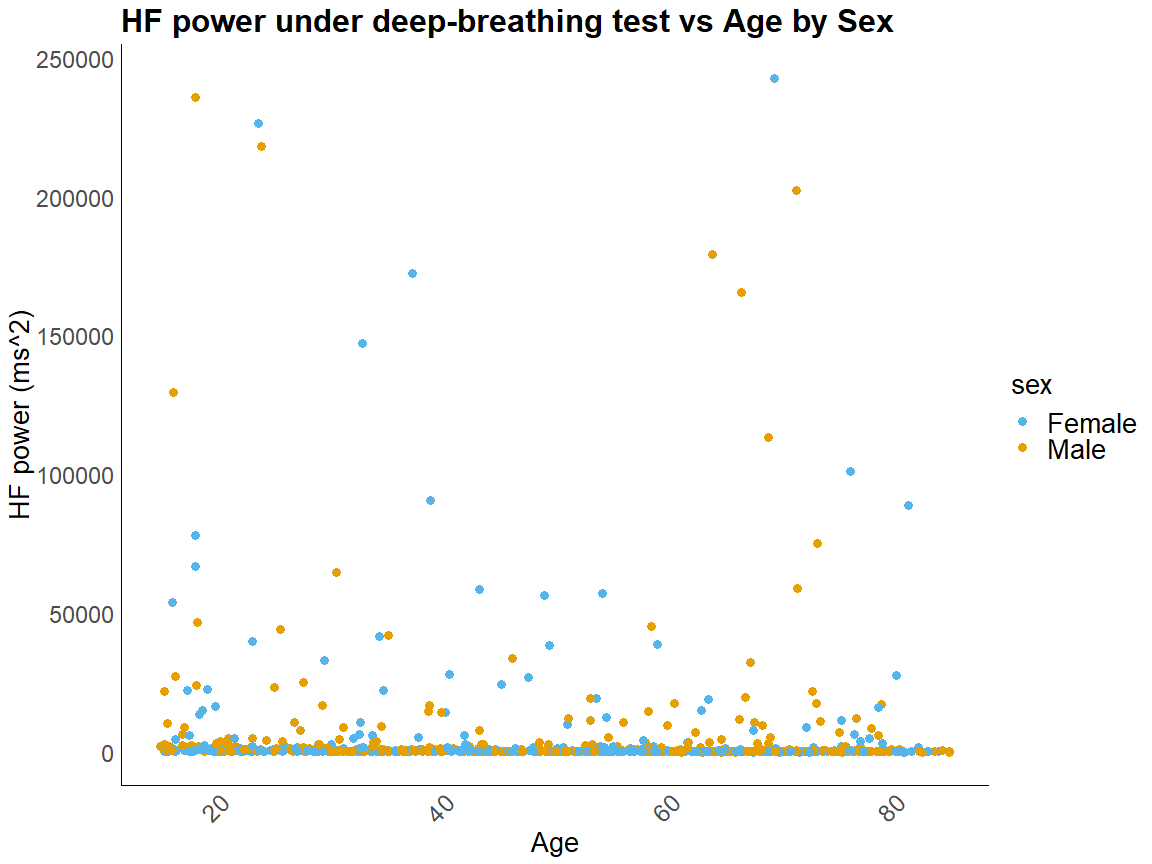

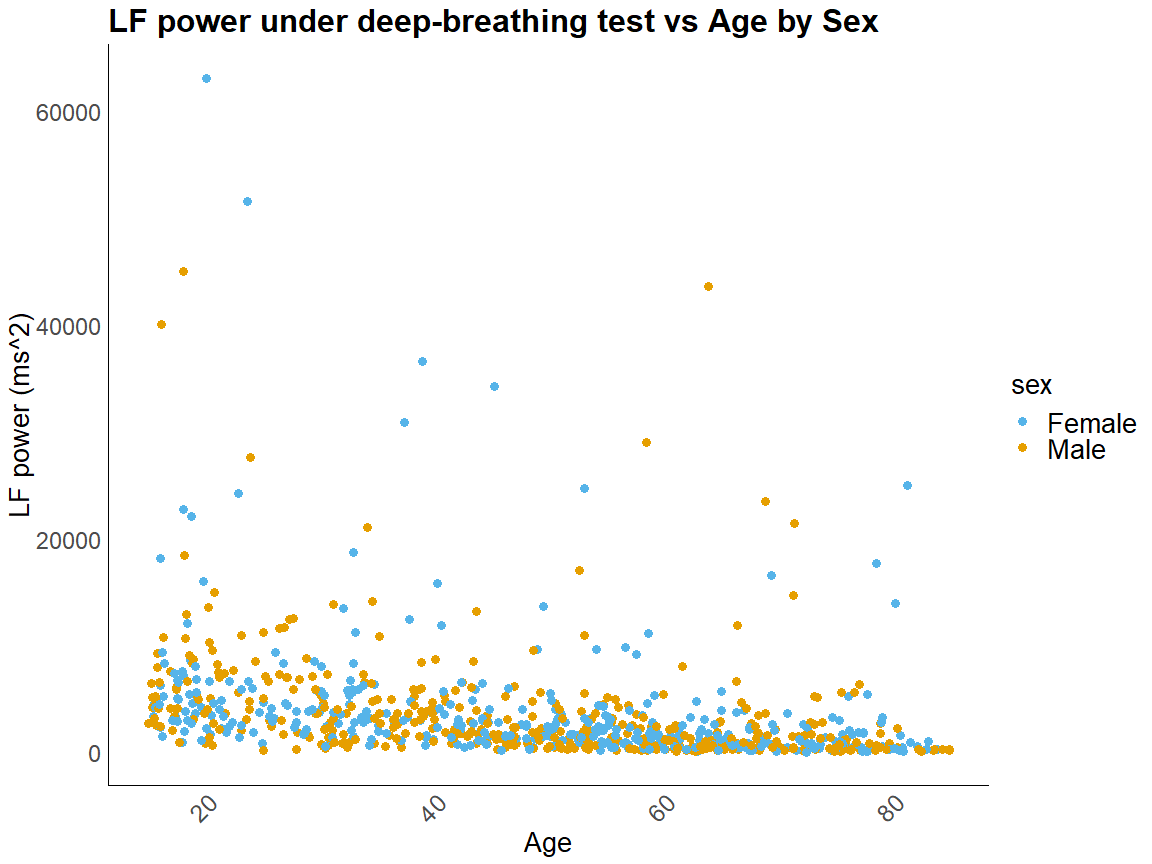

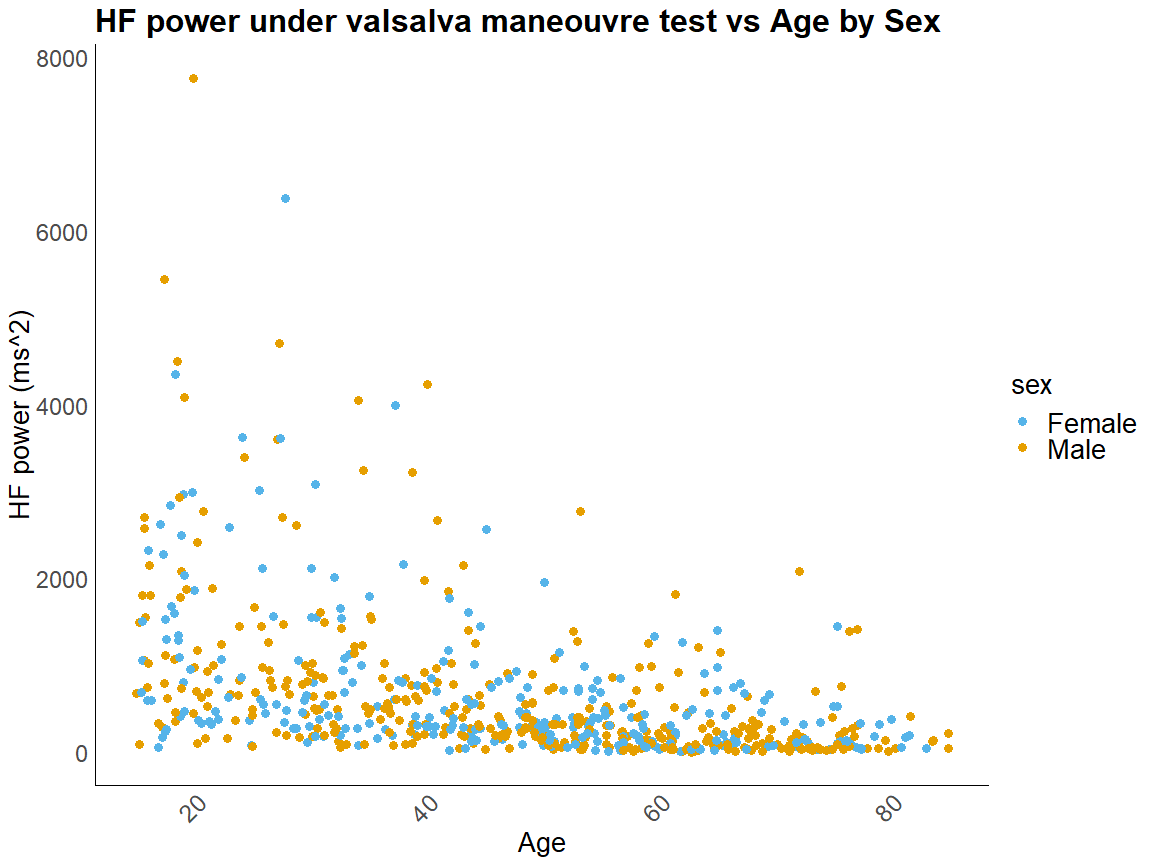

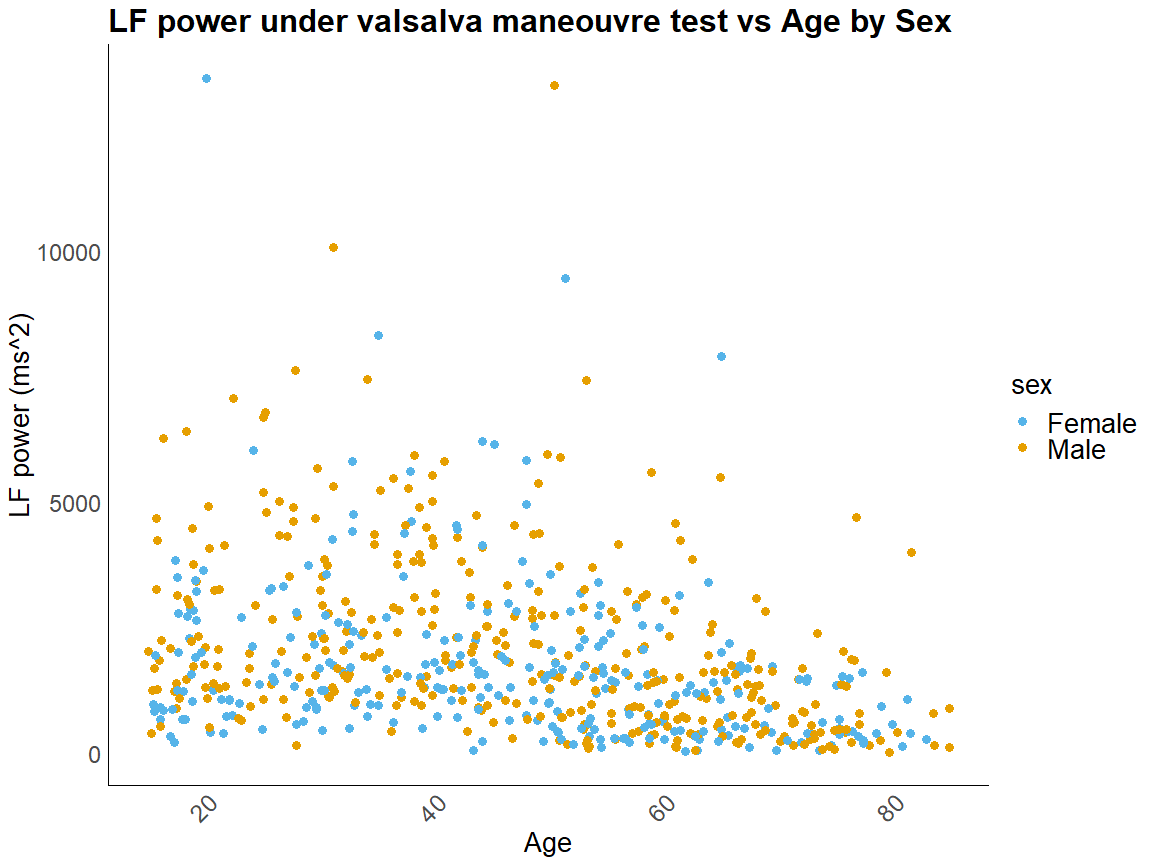


### Distribution of cardiovascular autonomic function measures by age group and sex

The violin plots show the distribution of the different indices by age groups of 5 years. The width of each "violin" represents the density of data, with wider sections indicating more data points. The plot reveals how the distribution varies across different age categories.
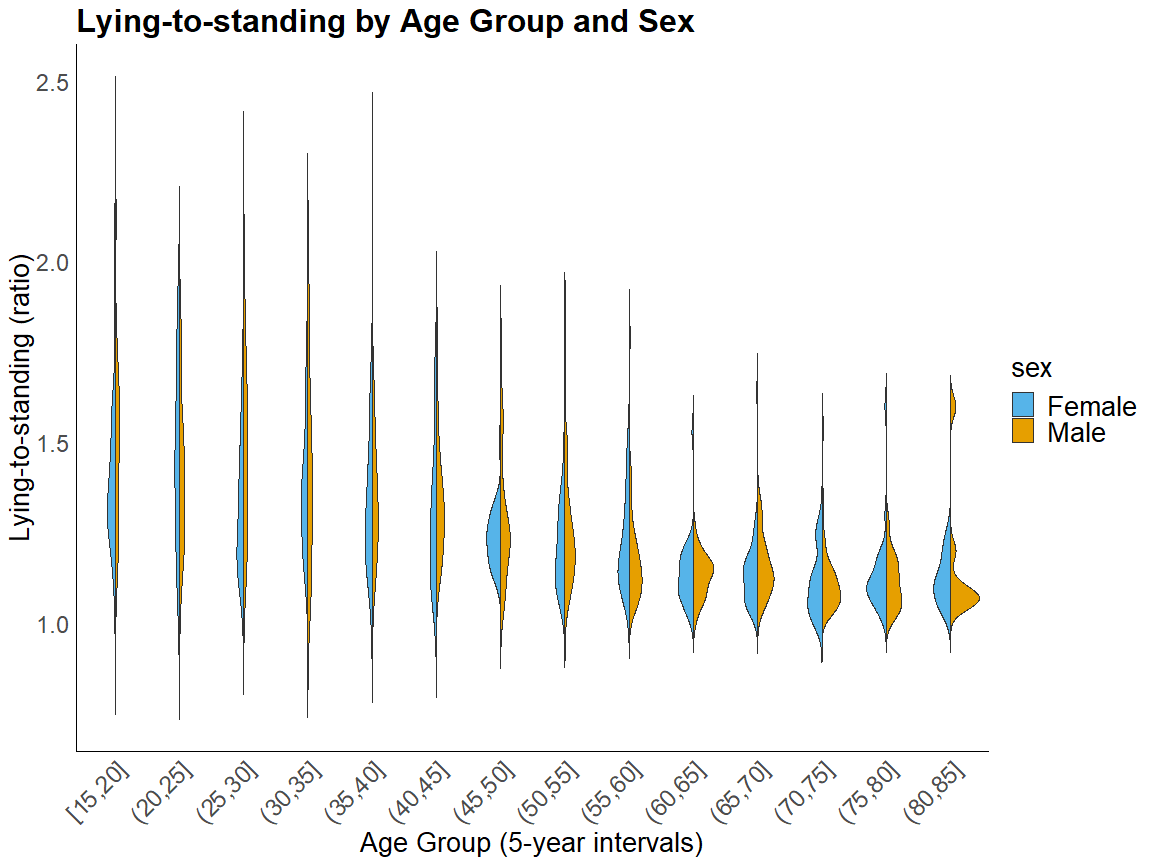

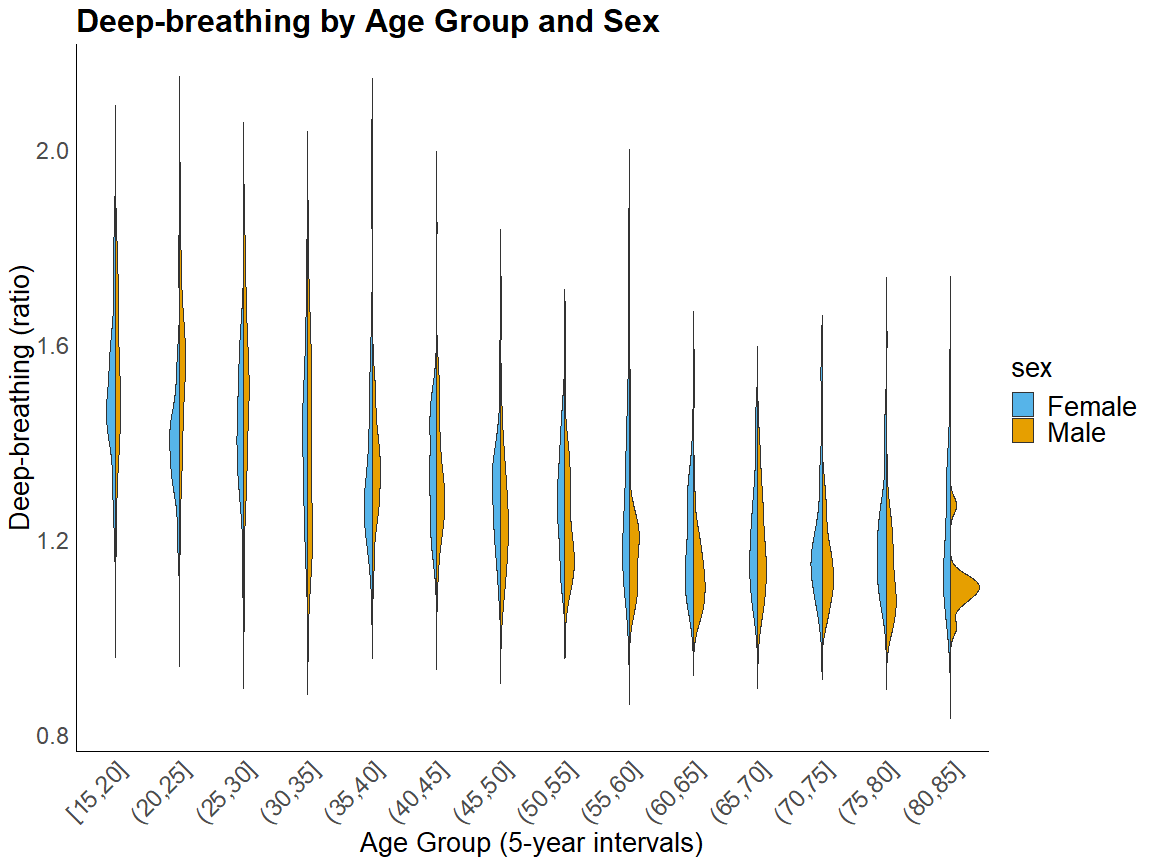

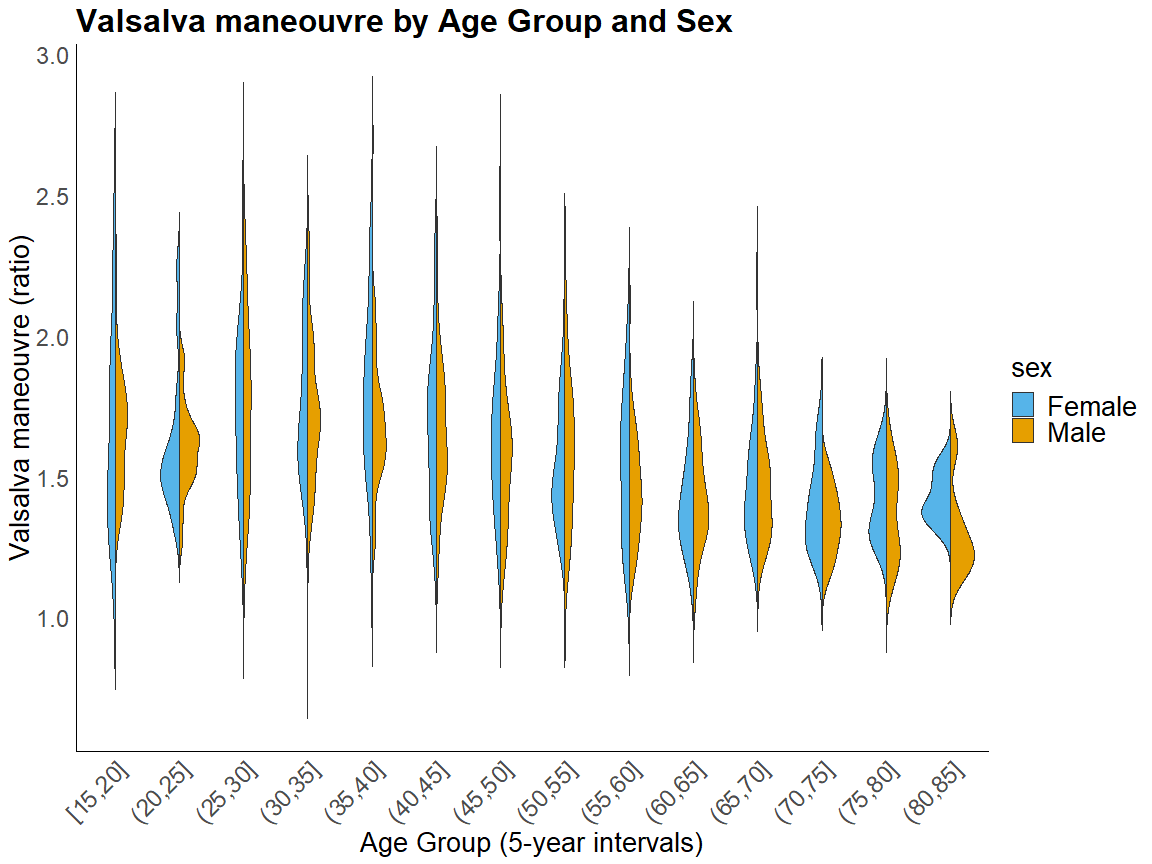

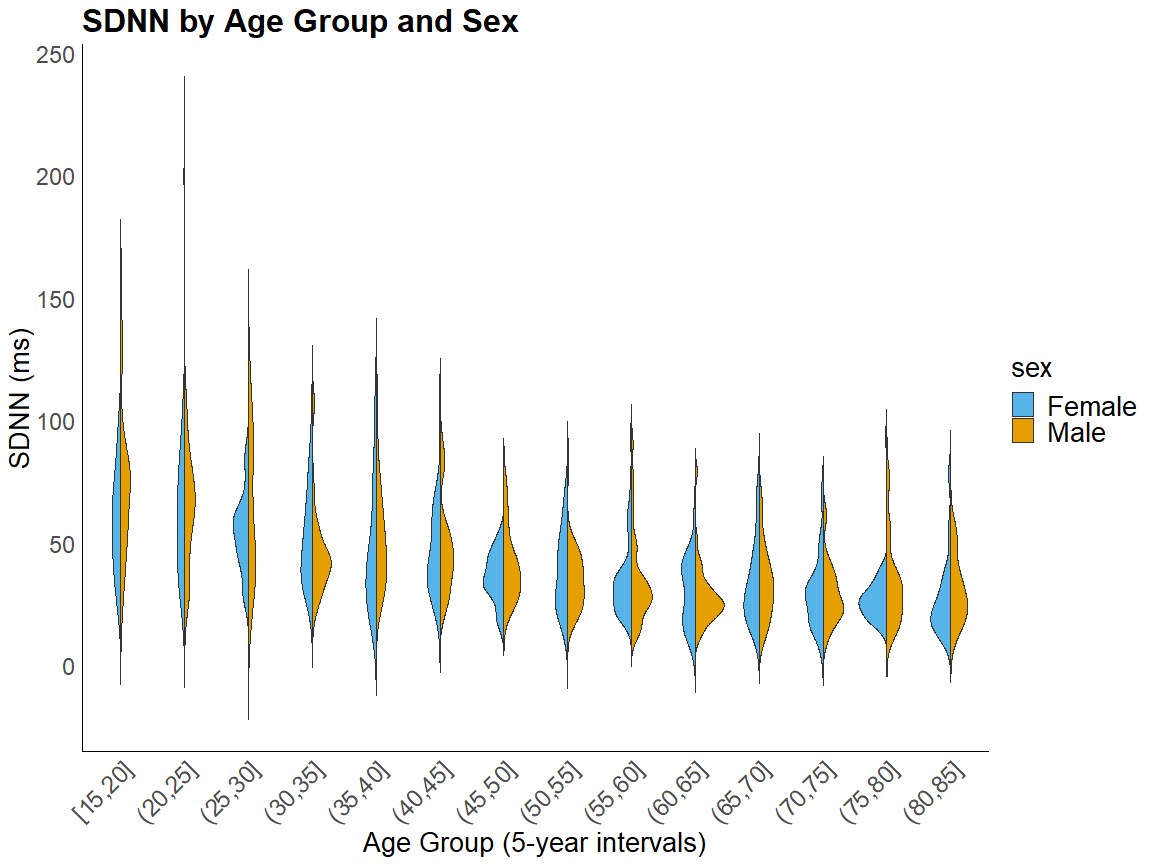

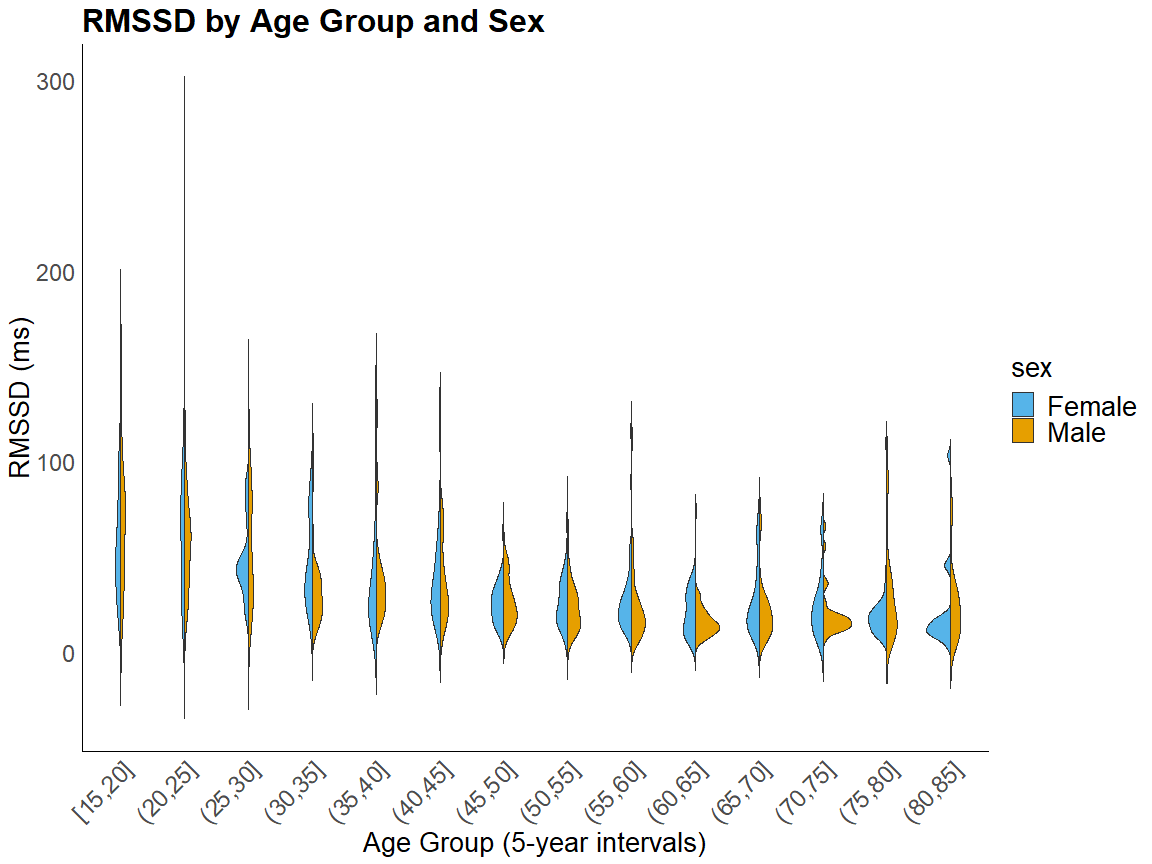

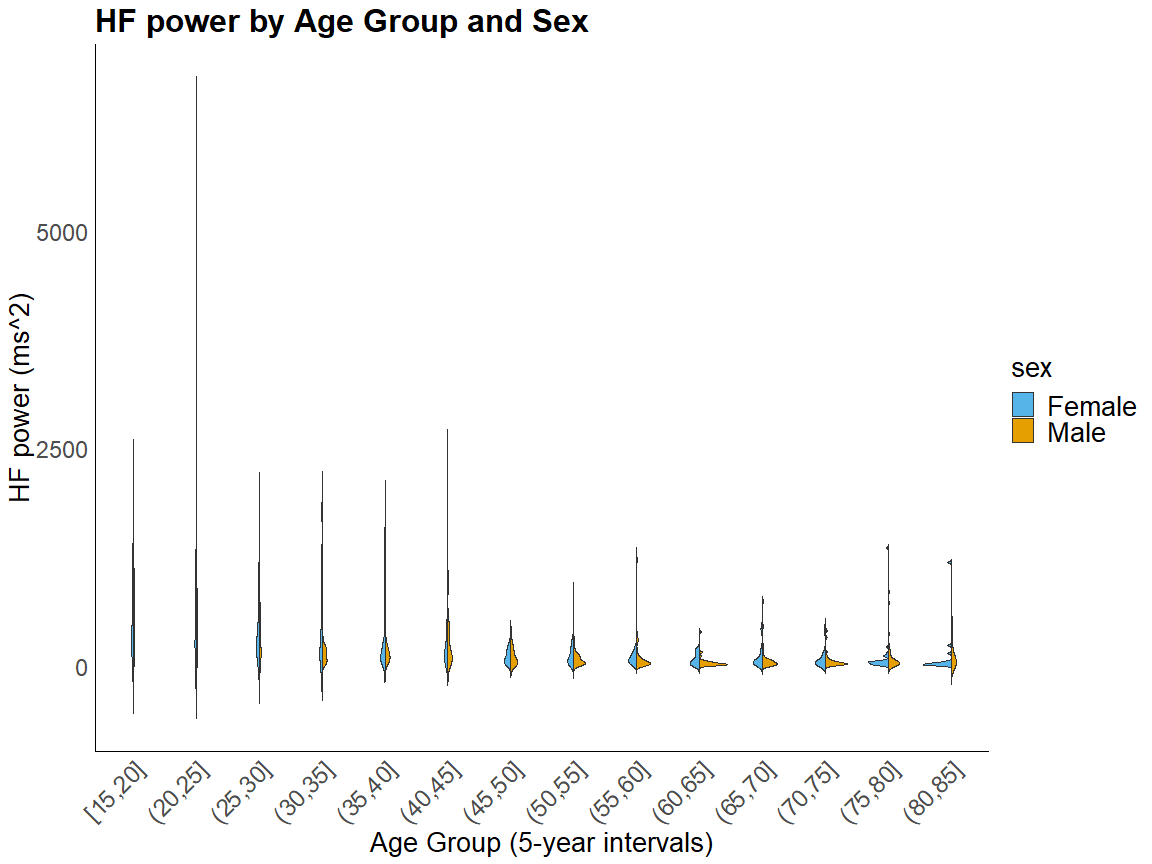

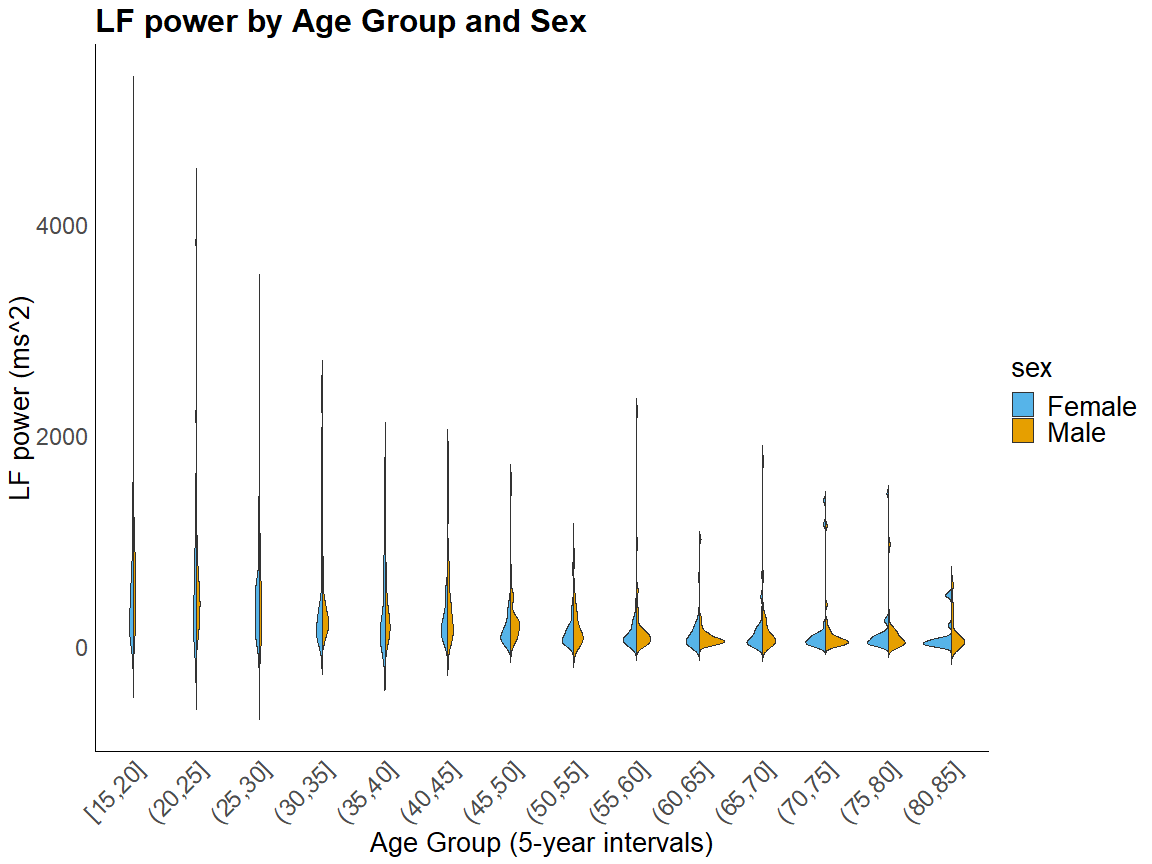

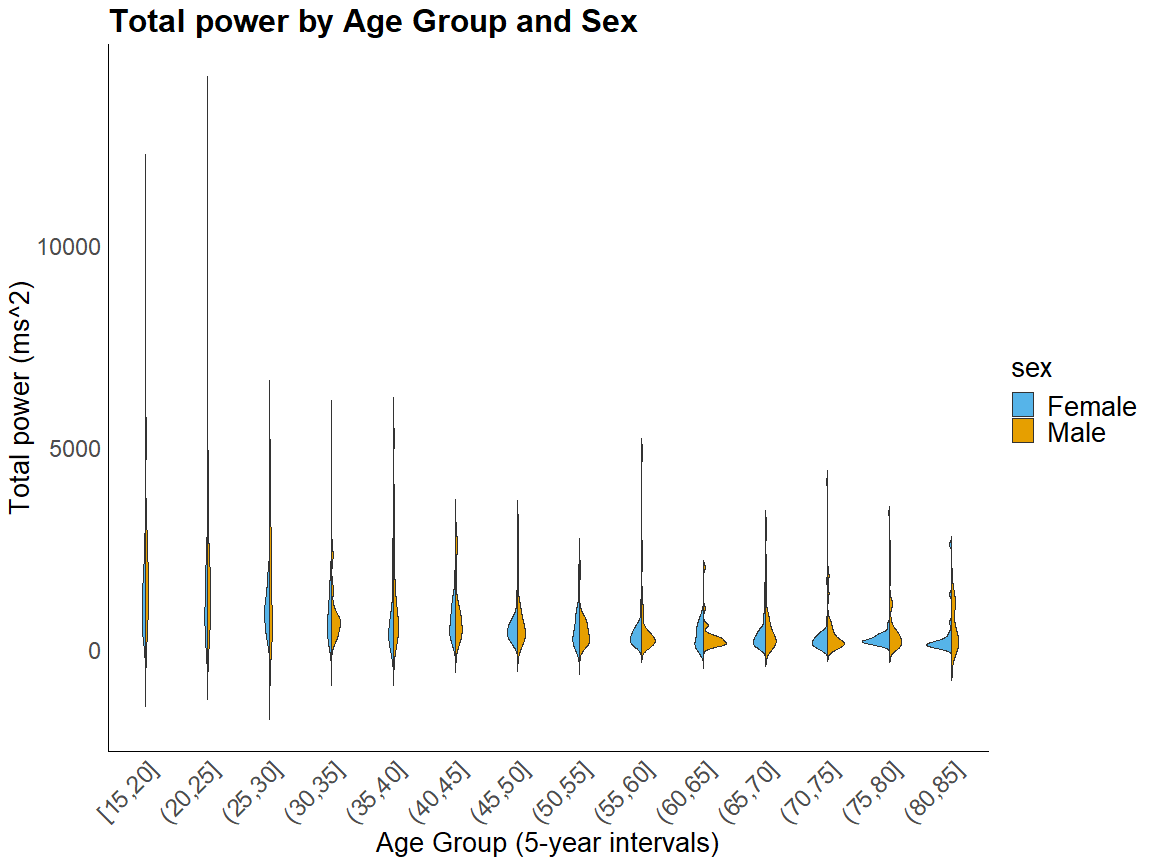

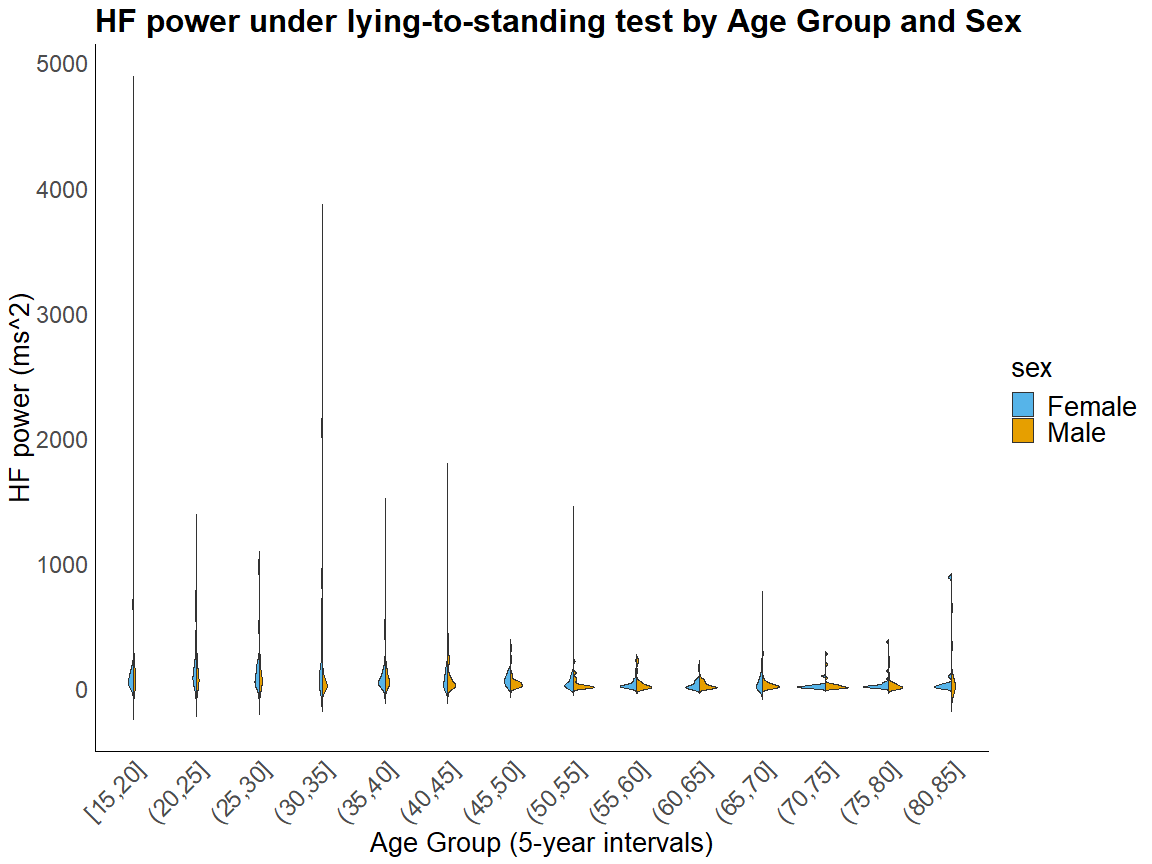

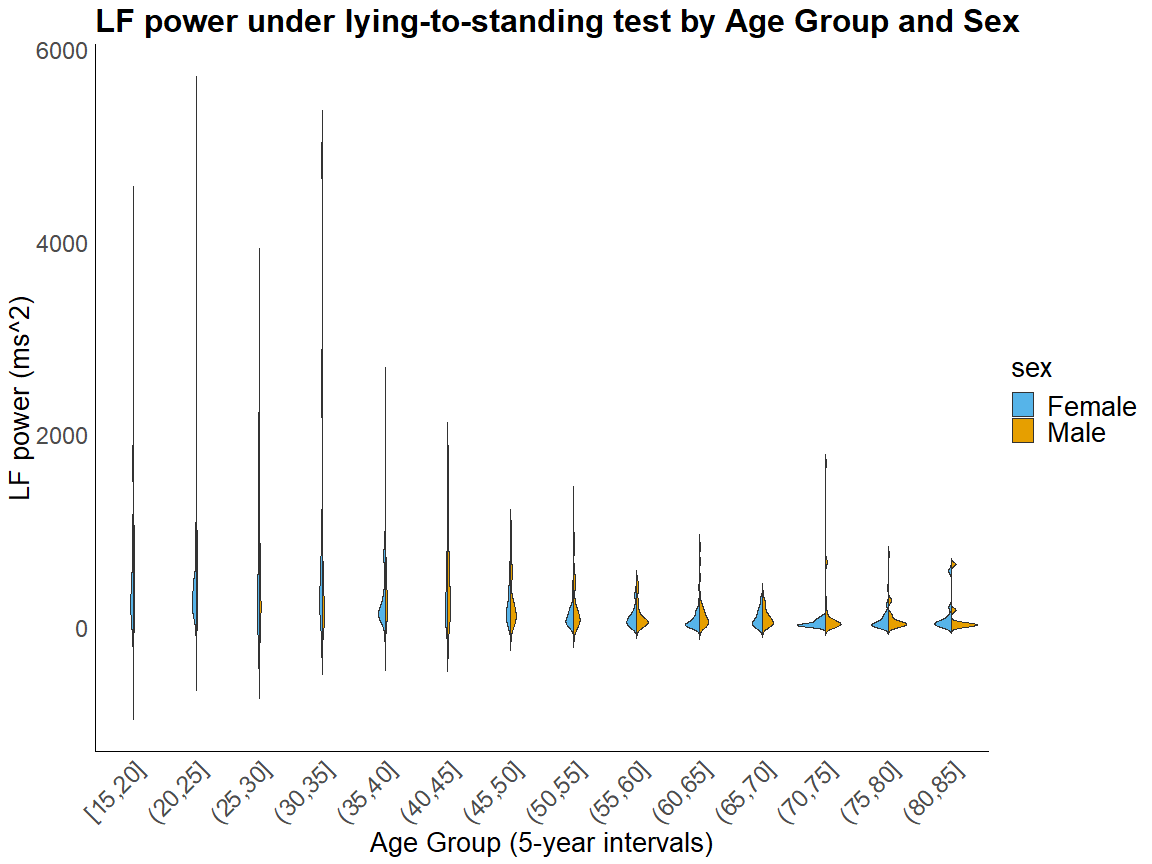

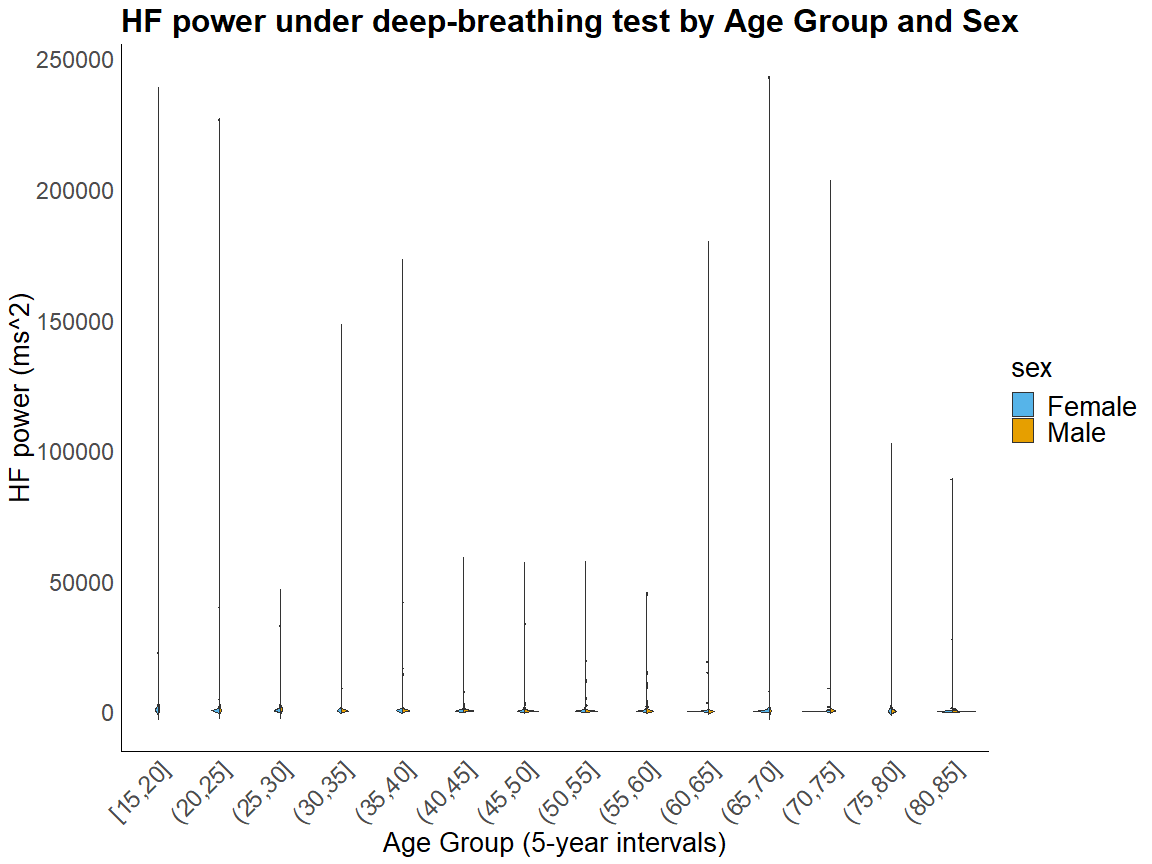

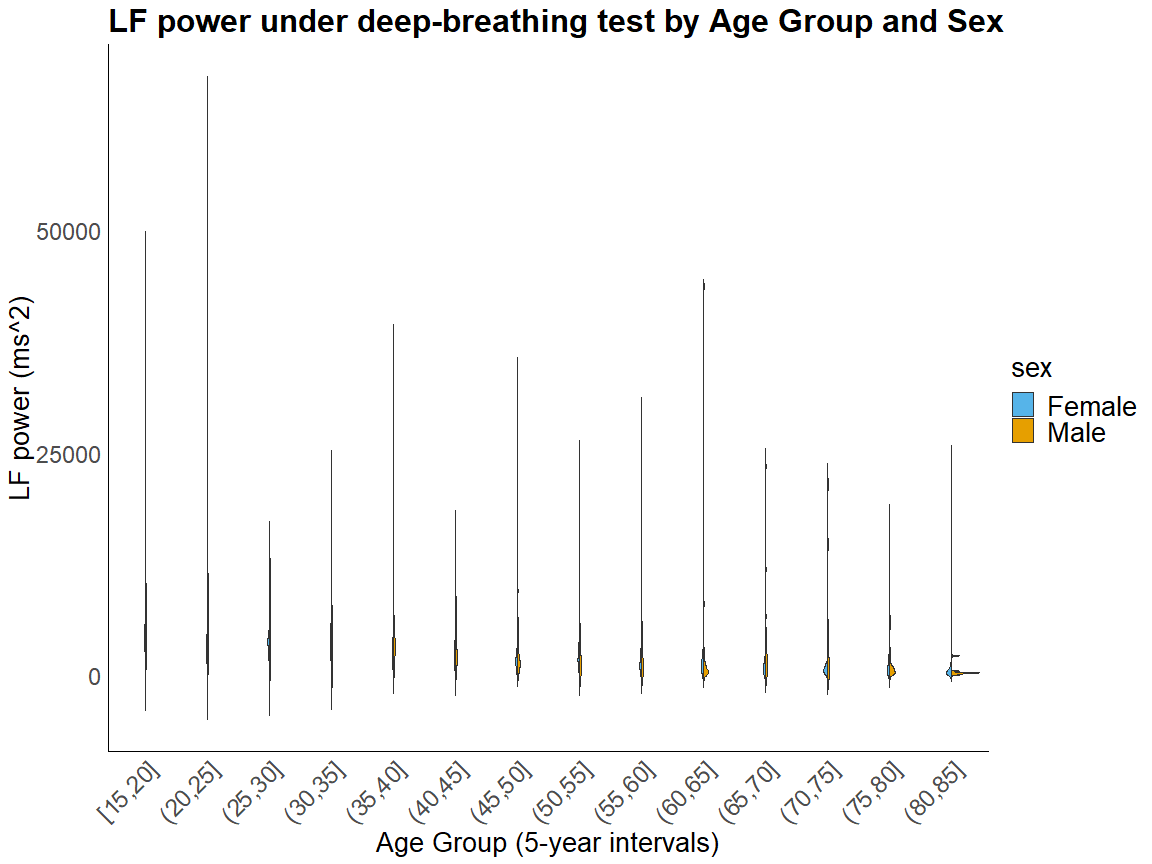

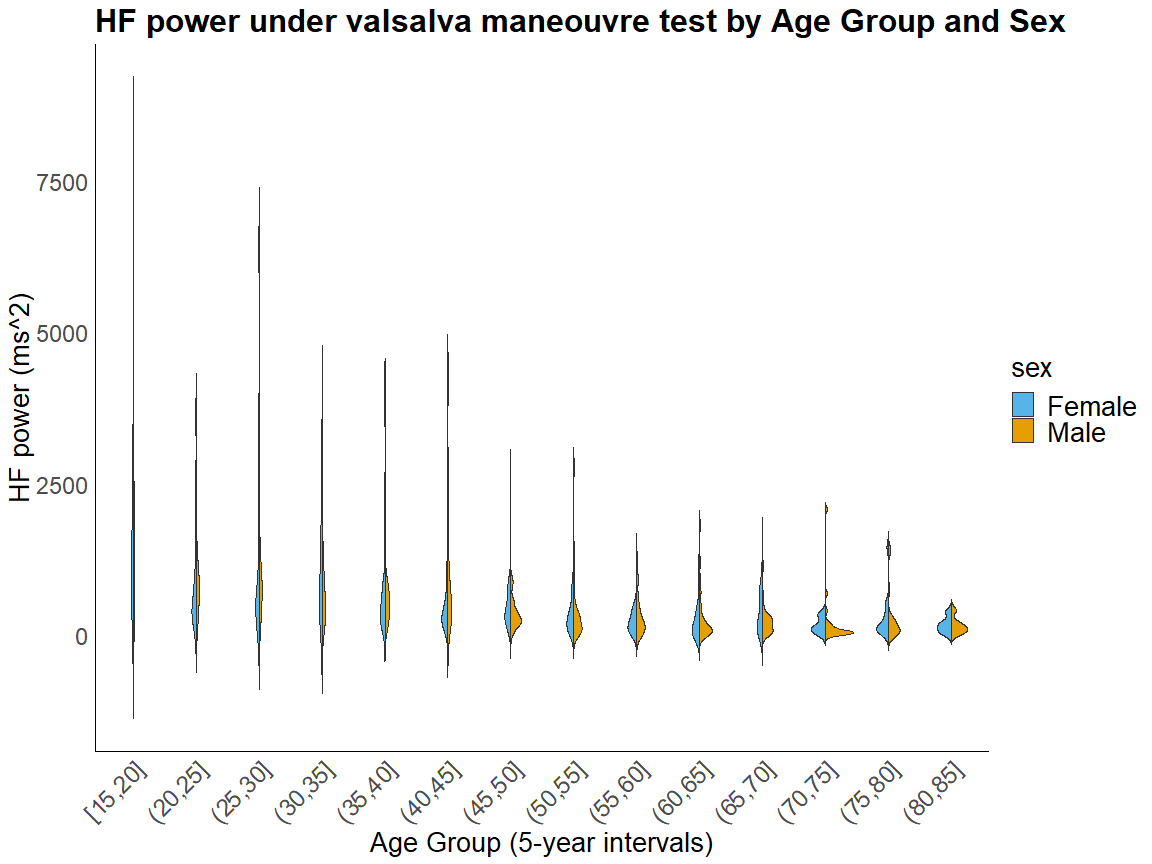

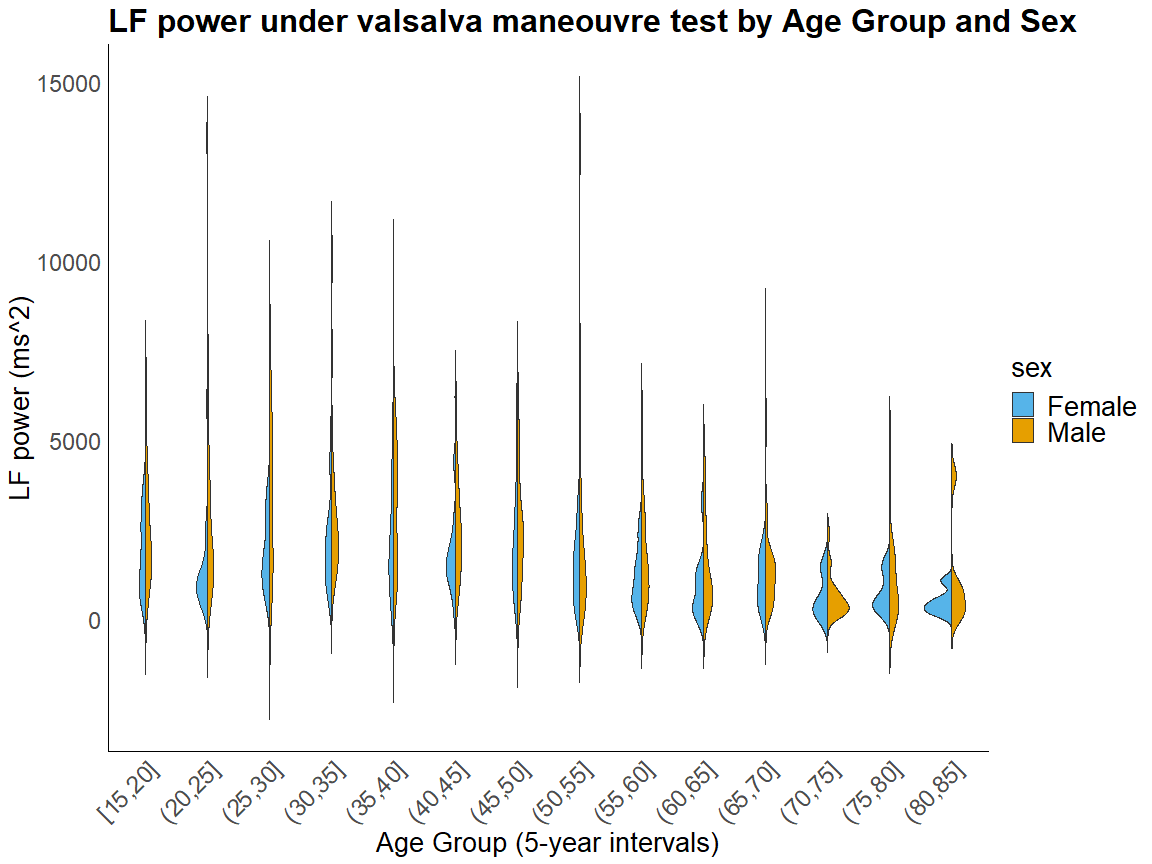


### Distribution of cardiovascular autonomic function measures:

| Appendix table 1 | | | | | | |
| --- | --- | --- | --- | --- | --- | --- |
| **Cardiovascular autonomic function** | **Pooled** | | **Females** | | **Males** | |
| *CARTs* | N | 875 |  | 421 |  | 454 |
| Lying-to-standing (ratio) | 739 | 1.21 [1.12;1.38] | 349 | 1.20 [1.12;1.35] | 390 | 1.21 [1.13;1.41] |
| Deep breathing (ratio) | 845 | 1.27 [1.16;1.42] | 408 | 1.29 [1.18;1.43] | 437 | 1.25 [1.14;1.41] |
| Valsalva manoeuvre (ratio) | 651 | 1.55 [1.38;1.74] | 282 | 1.56 [1.39;1.79] | 369 | 1.55 [1.37;1.72] |
| *HRV measures* |  |  |  |  |  |  |
| Heart rate (bpm) | 843 | 67 [61;74] | 409 | 68 [62;75] | 434 | 66 [60;73] |
| SDNN (ms) | 839 | 37 [27;54] | 409 | 37 [26;54] | 430 | 38 [27;54] |
| RMSSD (ms) | 839 | 27 [17;44] | 409 | 28 [17;45] | 430 | 26 [16;42] |
| LF power (ms²) | 839 | 152 [67;368] | 409 | 137 [60;334] | 430 | 175 [75;399] |
| HF power (ms²) | 839 | 100 [38;263] | 409 | 115 [46;270] | 430 | 87 [29;243] |
| Total power (ms²) | 839 | 486 [235;1,033] | 409 | 468 [229;1,027] | 430 | 519 [252;1,061] |
| LF [Lying-to-standing] (ms²) | 738 | 150 [52;392] | 349 | 142 [48;329] | 389 | 160 [58;482] |
| HF [Lying-to-standing] (ms²) | 738 | 39 [13;105] | 349 | 44 [16;111] | 389 | 34 [11;92] |
| LF [Deep breathing] (ms²) | 825 | 2166 [851;4296] | 398 | 2293 [1,007;4101] | 427 | 2,046 [753;4603] |
| HF [Deep breathing] (ms²) | 825 | 392 [171;1,155] | 398 | 367 [180;1,057] | 427 | 426 [164;1,334] |
| LF [Valsalva manoeuvre] (ms²) | 651 | 1,502 [727;2,693] | 282 | 1,332 [646;2,223] | 369 | 1,629 [838;2,958] |
| HF [Valsalva manoeuvre] (ms²) | 651 | 351 [145;804] | 282 | 363 [164;805] | 369 | 328 [131;796] |
| *Data are given in medians (IQR). CARTs, Cardiovascular autonomic reflex tests; HRV, heart rate variability; LF and HF power, low-frequency power high-frequency power; RMSSD, root mean square of the sum of the squares of differences between consecutive R–R intervals; SDNN, SD of normal-to-normal intervals.* | | | | | | |

## Influence of caffeine, food and exercise before examination on the outcomes

Linear regression models adjusted for age. When the outcome is an HRV measure, we also adjusted for heart rate.

**The association between caffeine and…**

Lying to standing ratio:

exp(Est.) 2.5% 97.5% P
(Intercept) 1.574 1.531 1.617 0.000
CaffeineNO 1.008 0.989 1.026 0.422
AgeVisit 0.995 0.995 0.996 0.000

Deep breathing ratio:

exp(Est.) 2.5% 97.5% P
(Intercept) 1.605 1.572 1.639 0.000
CaffeineNO 1.010 0.996 1.024 0.157
AgeVisit 0.995 0.995 0.996 0.000

Valsalva manoeuvre:

exp(Est.) 2.5% 97.5% P
(Intercept) 1.866 1.800 1.934 0.000
CaffeineNO 0.994 0.970 1.017 0.589
AgeVisit 0.996 0.996 0.997 0.000

SDNN:

exp(Est.) 2.5% 97.5% P
(Intercept) 302.584 248.537 368.385 0.000
CaffeineNO 1.018 0.966 1.072 0.511
AgeVisit 0.985 0.984 0.986 0.000
HR_BPM 0.980 0.978 0.983 0.000

RMSSD:

exp(Est.) 2.5% 97.5% P
(Intercept) 652.900 501.509 849.992 0.000
CaffeineNO 1.014 0.945 1.087 0.703
AgeVisit 0.980 0.978 0.982 0.000
HR_BPM 0.969 0.965 0.972 0.000

LF power:

exp(Est.) 2.5% 97.5% P
(Intercept) 8293.514 4932.312 13945.259 0.00
CaffeineNO 0.969 0.844 1.111 0.65
AgeVisit 0.964 0.960 0.967 0.00
HR_BPM 0.968 0.962 0.975 0.00

HF power:

exp(Est.) 2.5% 97.5% P
(Intercept) 28202.303 16153.564 49238.042 0.000
CaffeineNO 1.020 0.880 1.182 0.791
AgeVisit 0.958 0.954 0.962 0.000
HR_BPM 0.948 0.941 0.956 0.000

Total power:

exp(Est.) 2.5% 97.5% P
(Intercept) 29770.958 19428.618 45618.785 0.000
No caffeine 0.995 0.889 1.114 0.935
AgeVisit 0.970 0.967 0.972 0.000
HR_BPM 0.963 0.957 0.968 0.000

LF power during RS:

exp(Est.) 2.5% 97.5% P
(Intercept) 19267.911 10353.837 35856.503 0.000
No caffeine 0.991 0.842 1.167 0.915
AgeVisit 0.955 0.951 0.959 0.000
HR_BPM 0.960 0.952 0.968 0.000

HF power during RS:

exp(Est.) 2.5% 97.5% P
(Intercept) 5886.384 2805.302 12351.437 0.000
No caffeine 0.927 0.763 1.126 0.445
AgeVisit 0.963 0.958 0.968 0.000
HR_BPM 0.953 0.944 0.963 0.000

LF power during EI:

exp(Est.) 2.5% 97.5% P
(Intercept) 55674.665 33493.784 92544.587 0.00
No caffeine 0.990 0.866 1.131 0.88
AgeVisit 0.965 0.962 0.969 0.00
HR_BPM 0.976 0.969 0.982 0.00

HF power during EI:

exp(Est.) 2.5% 97.5% P
(Intercept) 19653.065 7999.725 48282.031 0.00
CaffeineNO 0.982 0.776 1.243 0.88
AgeVisit 0.975 0.969 0.981 0.00
HR_BPM 0.967 0.955 0.978 0.00

LF power during VM:

exp(Est.) 2.5% 97.5% P
(Intercept) 12330.168 7040.050 21595.448 0.000
CaffeineNO 0.954 0.827 1.100 0.514
AgeVisit 0.976 0.972 0.980 0.000
HR_BPM 0.984 0.976 0.991 0.000

HF power during VM:

exp(Est.) 2.5% 97.5% P
(Intercept) 9109.119 4718.166 17586.504 0.00
CaffeineNO 0.948 0.802 1.120 0.53
AgeVisit 0.959 0.955 0.964 0.00
HR_BPM 0.980 0.971 0.988 0.00

**The association between physical activity and…**

Lying to standing ratio:

exp(Est.) 2.5% 97.5% P
(Intercept) 1.540 1.489 1.593 0.000
**Physical_acti_NO 1.033 1.004 1.064 0.025**
AgeVisit 0.995 0.995 0.996 0.000

Deep breathing ratio:

exp(Est.) 2.5% 97.5% P
(Intercept) 1.626 1.585 1.669 0.000
Physical_acti_NO 0.992 0.971 1.014 0.491
AgeVisit 0.995 0.995 0.996 0.000

Valsalva manoeuvre:

exp(Est.) 2.5% 97.5% P
(Intercept) 1.797 1.721 1.876 0.000
**Physical_acti_NO 1.042 1.005 1.080 0.025**
AgeVisit 0.996 0.996 0.997 0.000

SDNN:

exp(Est.) 2.5% 97.5% P
(Intercept) 300.928 243.259 372.268 0.000
Physical_acti_NO 1.016 0.936 1.102 0.709
AgeVisit 0.985 0.984 0.986 0.000
HR_BPM 0.980 0.978 0.983 0.000

RMSSD:

exp(Est.) 2.5% 97.5% P
(Intercept) 680.314 511.533 904.786 0.000
Physical_acti_NO 0.970 0.870 1.082 0.587
AgeVisit 0.980 0.978 0.981 0.000
HR_BPM 0.968 0.965 0.972 0.000

LF power:

exp(Est.) 2.5% 97.5% P
(Intercept) 8563.497 4882.947 15018.283 0.000
Physical_acti_NO 0.952 0.768 1.181 0.657
AgeVisit 0.964 0.960 0.967 0.000
HR_BPM 0.968 0.961 0.975 0.000

HF power:

exp(Est.) 2.5% 97.5% P
(Intercept) 27742.621 15188.899 50672.076 0.000
Physical_acti_NO 1.027 0.816 1.294 0.818
AgeVisit 0.958 0.954 0.961 0.000
HR_BPM 0.949 0.941 0.956 0.000

Total power:

exp(Est.) 2.5% 97.5% P
(Intercept) 29038.912 18307.209 46061.548 0.000
Physical_acti_NO 1.021 0.856 1.218 0.821
AgeVisit 0.970 0.967 0.972 0.000
HR_BPM 0.963 0.957 0.968 0.000

LF power during RS:

exp(Est.) 2.5% 97.5% P
(Intercept) 23152.968 11896.255 45061.235 0.000
Physical_acti_NO 0.834 0.645 1.077 0.164
AgeVisit 0.956 0.952 0.960 0.000
HR_BPM 0.960 0.952 0.968 0.000

HF power during RS:

exp(Est.) 2.5% 97.5% P
(Intercept) 4750.639 2144.304 10524.893 0.000
Physical_acti_NO 1.168 0.860 1.586 0.319
AgeVisit 0.963 0.959 0.968 0.000
HR_BPM 0.954 0.944 0.963 0.000

LF power during EI:

exp(Est.) 2.5% 97.5% P
(Intercept) 58420.396 33732.309 101177.262 0.000
Physical_acti_NO 0.951 0.772 1.170 0.633
AgeVisit 0.966 0.962 0.969 0.000
HR_BPM 0.975 0.969 0.982 0.000

HF power during EI:

exp(Est.) 2.5% 97.5% P
(Intercept) 20389.038 7717.343 53867.362 0.000
Physical_acti_NO 0.957 0.662 1.382 0.813
AgeVisit 0.975 0.969 0.981 0.000
HR_BPM 0.966 0.955 0.978 0.000

LF power during VM:

exp(Est.) 2.5% 97.5% P
(Intercept) 11634.249 6351.591 21310.526 0.000
Physical_acti_NO 1.027 0.825 1.279 0.812
AgeVisit 0.976 0.973 0.980 0.000
HR_BPM 0.984 0.976 0.991 0.000

HF power during VM:

exp(Est.) 2.5% 97.5% P
(Intercept) 8894.974 4371.093 18100.866 0.000
Physical_acti_NO 0.992 0.767 1.284 0.954
AgeVisit 0.960 0.955 0.964 0.000
HR_BPM 0.980 0.971 0.988 0.000

**The association between a meal and…**

Lying to standing ratio:

exp(Est.) 2.5% 97.5% P
(Intercept) 1.578 1.539 1.619 0.000
Meal_consumpt_NO 1.006 0.988 1.025 0.518
AgeVisit 0.995 0.995 0.996 0.000

Deep breathing ratio:

exp(Est.) 2.5% 97.5% P
(Intercept) 1.622 1.590 1.654 0.000
Meal_consumpt_NO 0.992 0.978 1.006 0.252
AgeVisit 0.995 0.995 0.996 0.000

Valsalva manoeuvre:

exp(Est.) 2.5% 97.5% P
(Intercept) 1.849 1.788 1.911 0.000
Meal_consumpt_NO 1.012 0.989 1.036 0.313
AgeVisit 0.996 0.996 0.997 0.000

SDNN:

exp(Est.) 2.5% 97.5% P
(Intercept) 306.117 251.731 372.252 0.00
Meal_consumpt_NO 0.999 0.948 1.053 0.97
AgeVisit 0.985 0.984 0.986 0.00
HR_BPM 0.980 0.978 0.983 0.00

RMSSD:

exp(Est.) 2.5% 97.5% P
(Intercept) 654.283 504.731 848.147 0.000
No meal 0.983 0.917 1.054 0.637
AgeVisit 0.980 0.978 0.981 0.000
HR_BPM 0.969 0.965 0.972 0.000

LF power:

exp(Est.) 2.5% 97.5% P
(Intercept) 8033.206 4792.723 13464.661 0.00
Meal_consumpt_NO 1.025 0.892 1.176 0.73
AgeVisit 0.964 0.960 0.967 0.00
HR_BPM 0.968 0.962 0.975 0.00

HF power:

exp(Est.) 2.5% 97.5% P
(Intercept) 28707.865 16499.529 49949.394 0.000
Meal_consumpt_NO 0.989 0.853 1.147 0.888
AgeVisit 0.958 0.954 0.961 0.000
HR_BPM 0.948 0.941 0.956 0.000

Total power:

exp(Est.) 2.5% 97.5% P
(Intercept) 29532.678 19324.096 45134.277 0.000
Meal_consumpt_NO 1.011 0.902 1.132 0.855
AgeVisit 0.970 0.967 0.972 0.000
HR_BPM 0.963 0.957 0.968 0.000

LF power during RS:

(Intercept) 19353.827 10450.410 35842.674 0.000
Meal_consumpt_NO 0.979 0.831 1.154 0.799
AgeVisit 0.955 0.951 0.959 0.000
HR_BPM 0.960 0.952 0.968 0.000

HF power during RS:

exp(Est.) 2.5% 97.5% P
(Intercept) 5589.812 2678.489 11665.533 0.000
Meal_consumpt_NO 0.999 0.821 1.215 0.993
AgeVisit 0.964 0.959 0.968 0.000
HR_BPM 0.953 0.944 0.963 0.000

LF power during EI:

exp(Est.) 2.5% 97.5% P
(Intercept) 56789.422 34253.349 94152.502 0.000
Meal_consumpt_NO 0.946 0.828 1.081 0.412
AgeVisit 0.965 0.962 0.969 0.000
HR_BPM 0.976 0.969 0.982 0.000

HF power during EI:

exp(Est.) 2.5% 97.5% P
(Intercept) 19389.831 7925.972 47434.629 0.000
Meal_consumpt_NO 1.006 0.795 1.273 0.961
AgeVisit 0.975 0.969 0.981 0.000
HR_BPM 0.967 0.955 0.978 0.000

LF power during VM:

exp(Est.) 2.5% 97.5% P
(Intercept) 12031.803 6883.475 21030.699 0.000
Meal_consumpt_NO 0.992 0.860 1.144 0.911
AgeVisit 0.976 0.973 0.980 0.000
HR_BPM 0.984 0.976 0.991 0.000

HF power during VM:

exp(Est.) 2.5% 97.5% P
(Intercept) 9369.221 4869.610 18026.557 0.000
Meal_consumpt_NO 0.884 0.748 1.045 0.149
AgeVisit 0.960 0.955 0.964 0.000
HR_BPM 0.980 0.971 0.988 0.000

**The association between examination time and…**

Lying to standing ratio:

exp(Est.) 2.5% 97.5% P
(Intercept) 1.579 1.542 1.618 0.000
examination before 12 pm 0.990 0.971 1.010 0.318
AgeVisit 0.995 0.995 0.996 0.000

Deep breathing ratio:

exp(Est.) 2.5% 97.5% P
(Intercept) 1.612 1.582 1.642 0.000
examination before 12 pm 1.009 0.994 1.025 0.235
AgeVisit 0.995 0.995 0.996 0.000

Valsalva manoeuvre:

exp(Est.) 2.5% 97.5% P
(Intercept) 1.860 1.801 1.919 0.00
examination before 12 pm 1.006 0.980 1.033 0.622
AgeVisit 0.996 0.996 0.997 0.00

SDNN:

exp(Est.) 2.5% 97.5% P

(Intercept) 313.024 258.394 379.203 0.000
examination before 12 pm 1.050 0.992 1.112 0.097
AgeVisit 0.985 0.984 0.986 0.000
HR_BPM 0.980 0.977 0.982 0.000

RMSSD:

exp(Est.) 2.5% 97.5% P
(Intercept) 656.896 507.857 849.673 0.00
examination before 12 pm 1.076 0.994 1.159 0.062
AgeVisit 0.980 0.978 0.981 0.00
HR_BPM 0.968 0.965 0.972 0.00

LF power:

exp(Est.) 2.5% 97.5% P
(Intercept) 8529.615 5140.879 14152.121 0.000
examination before 12 pm 1.016 0.874 1.181 0.839
AgeVisit 0.964 0.960 0.967 0.000
HR_BPM 0.967 0.961 0.974 0.000

HF power:

exp(Est.) 2.5% 97.5% P
(Intercept) 29250.629 16981.071 50385.475 0.000
**examination before 12 pm 1.193 1.006 1.391 0.033**
AgeVisit 0.957 0.954 0.961 0.000
HR_BPM 0.948 0.941 0.955 0.000

Total power:

exp(Est.) 2.5% 97.5% P
(Intercept) 31290.378 20646.255 47422.050 0.000
examination before 12 pm 1.084 0.958 1.227 0.208
AgeVisit 0.969 0.967 0.972 0.000
HR_BPM 0.962 0.956 0.967 0.000

LF power during RS:

exp(Est.) 2.5% 97.5% P
(Intercept) 22744.340 12367.518 41827.715 0.000
examination before 12 pm 0.952 0.799 1.144 0.589
AgeVisit 0.956 0.952 0.960 0.000
HR_BPM 0.957 0.950 0.965 0.000

HF power during RS:

exp(Est.) 2.5% 97.5% P
(Intercept) 6025.679 2925.035 12413.116 0.000
examination before 12 pm 1.058 0.855 1.309 0.591
AgeVisit 0.964 0.959 0.969 0.000
HR_BPM 0.952 0.943 0.961 0.000

LF power during EI:

exp(Est.) 2.5% 97.5% P
(Intercept) 57189.983 34775.544 94051.560 0.000
examination before 12 pm 1.035 0.890 1.193 0.65
AgeVisit 0.966 0.962 0.969 0.000
HR_BPM 0.975 0.968 0.981 0.000

HF power during EI:

exp(Est.) 2.5% 97.5% P
(Intercept) 18456.970 7647.027 44547.997 0.00
examination before 12 pm 1.022 0.776 1.305 0.86
AgeVisit 0.976 0.970 0.982 0.00
HR_BPM 0.967 0.955 0.978 0.00

LF power during VM:

exp(Est.) 2.5% 97.5% P
(Intercept) 12740.699 7341.983 22109.206 0.000
**examination before 12 pm 1.182 1.011 1.390 0.038**AgeVisit 0.975 0.971 0.979 0.000
HR_BPM 0.983 0.976 0.990 0.000

HF power during VM:

exp(Est.) 2.5% 97.5% P
(Intercept) 9642.857 5063.648 18363.180 0.000
**examination before 12 pm 1.23 1.016 1.474 0.029**
AgeVisit 0.959 0.954 0.963 0.000
HR_BPM 0.978 0.970 0.987 0.000

# Missing data

Missing data for Valsalva manoeuvre:


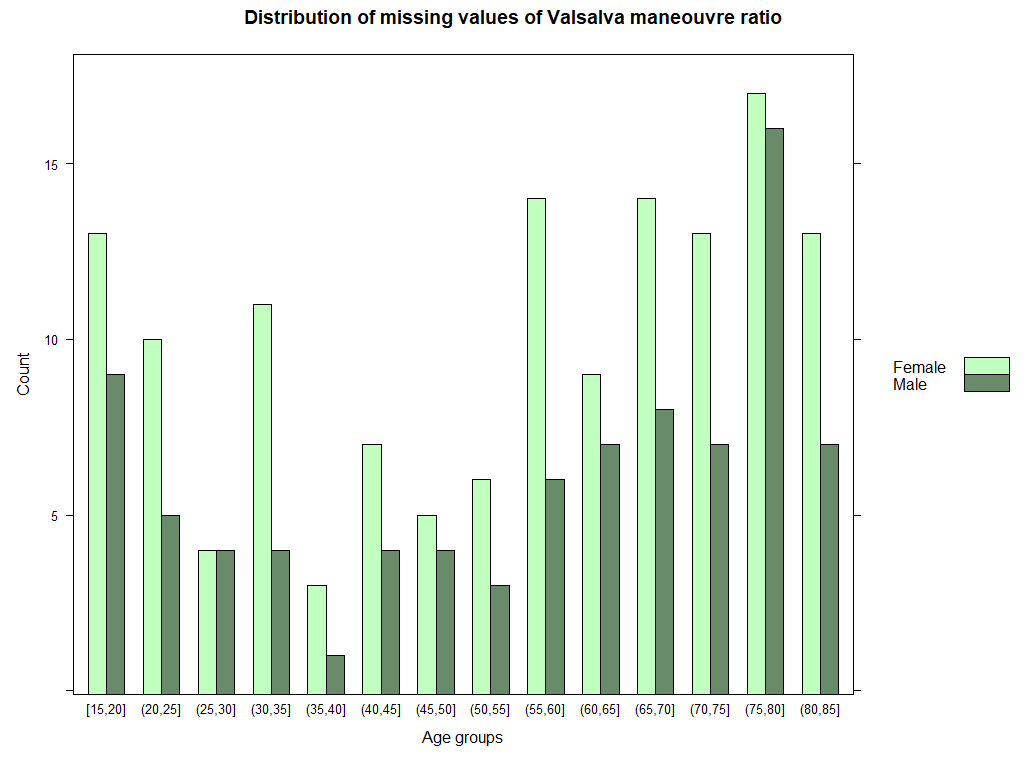


Missing data for lying-to-standing:


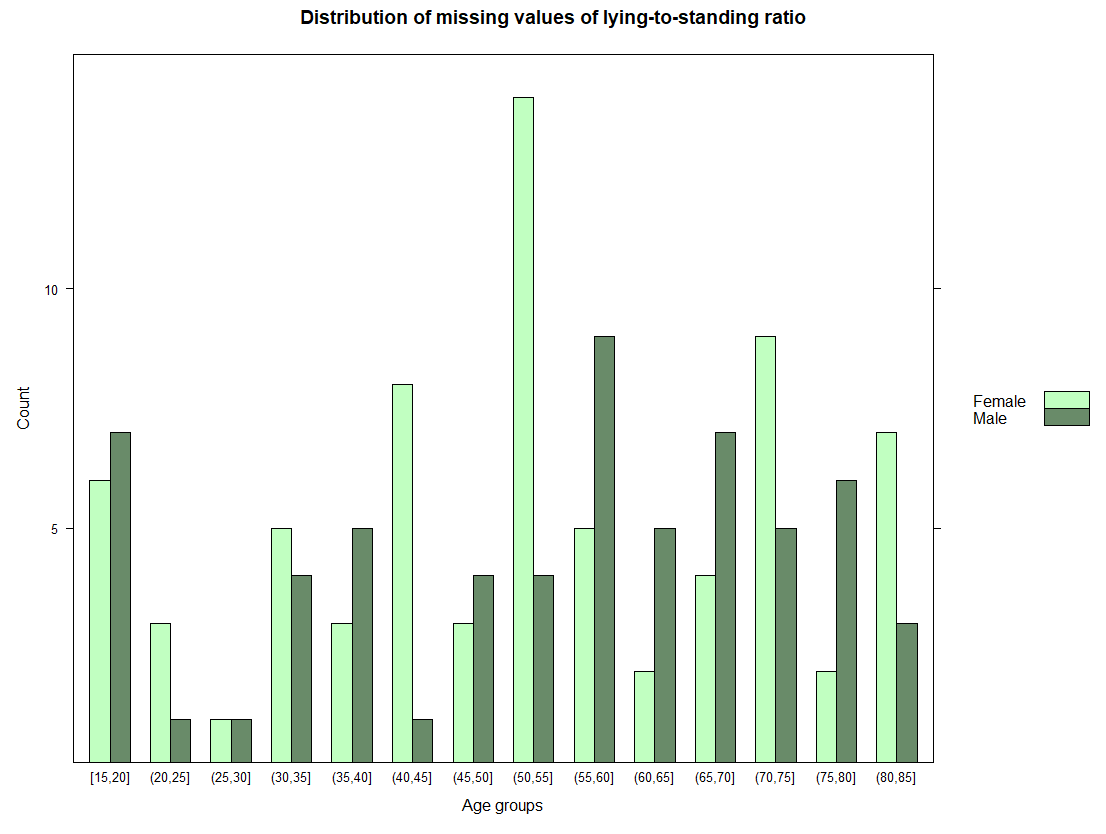


We have applied logistic regression models to assess whether the missingness of lying-to-standing or Valsalva manoeuvre was dependent on age and sex:

exp(Est.) 2.5% 97.5% P
(Intercept) 0.164 0.102 0.264 0
AgeVisit 1.022 1.014 1.031 0
sexMale 0.459 0.334 0.630 0

Table: Logistic regression, the association between age + sex and missingness of VM

exp(Est.) 2.5% 97.5% P
(Intercept) 0.102 0.058 0.180 0.000
AgeVisit 1.014 1.004 1.024 0.005
sexMale 0.797 0.552 1.152 0.227

Table: Logistic regression, the association between age + sex and missingness of RS

We have plotted the predicted association between age and the missingness of lying-to-standing maneouvre and Valsalva manoeuvre for each sex. The missingness of Valsalva manoeuvre is dependent on both age and sex. Whereas the missingness of lying-to-standing manoeuvre is only dependent on age. The dotted lines are illustrating the general level of missingness for females (orange) and males (blue).

Missingness of Valsalva manoeuvre:
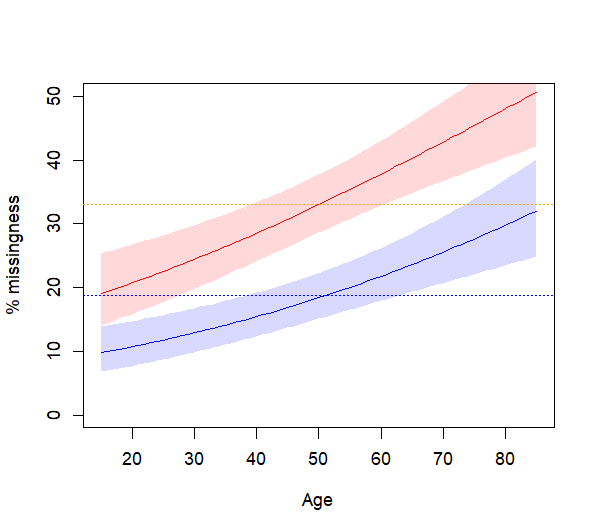


Missingness of lying-to-standing:
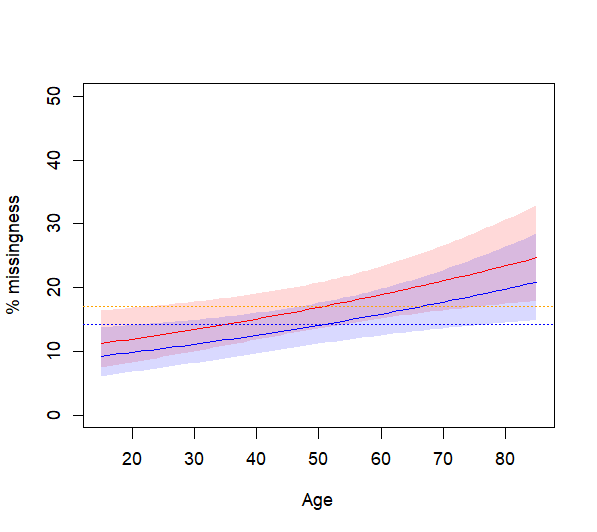


# Quantile regression models

We examined the association between age and all outcomes of cardiovascular autonomic function by applying quantile regression models at the lowest 5^th^ and 10^th^ percentile. When the association to the CARTs were assessed, we used a log(y-1) transformation of the outcome while a regular log(y) transformation was applied when HRV measures were included as outcomes. The estimates displayed at the y-axis have been back-transformed.

## CARTs:

### Lying-to-standing:


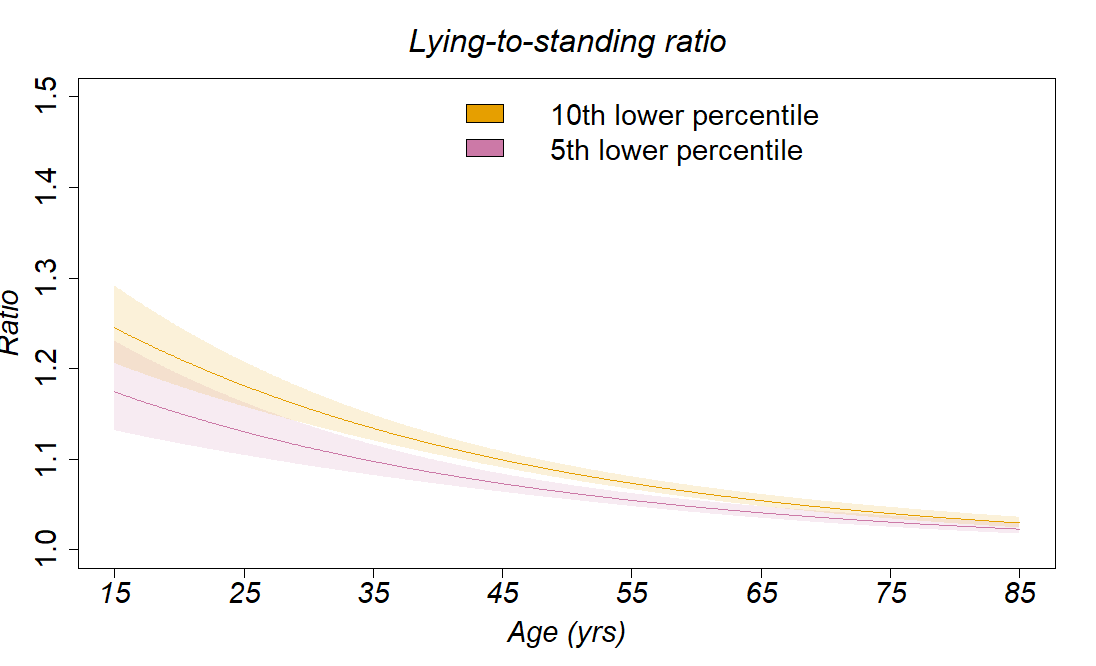


The 5^th^ lowest percentile:

exp(Est.) 2.5% 97.5% P
(Intercept) 0.269 0.185 0.391 0
AgeVisit 0.971 0.965 0.978 0

The 10^th^ lowest percentile:
 exp(Est.) 2.5% 97.5% P
(Intercept) 0.385 0.304 0.488 0
AgeVisit 0.970 0.966 0.975 0

### Deep breathing:


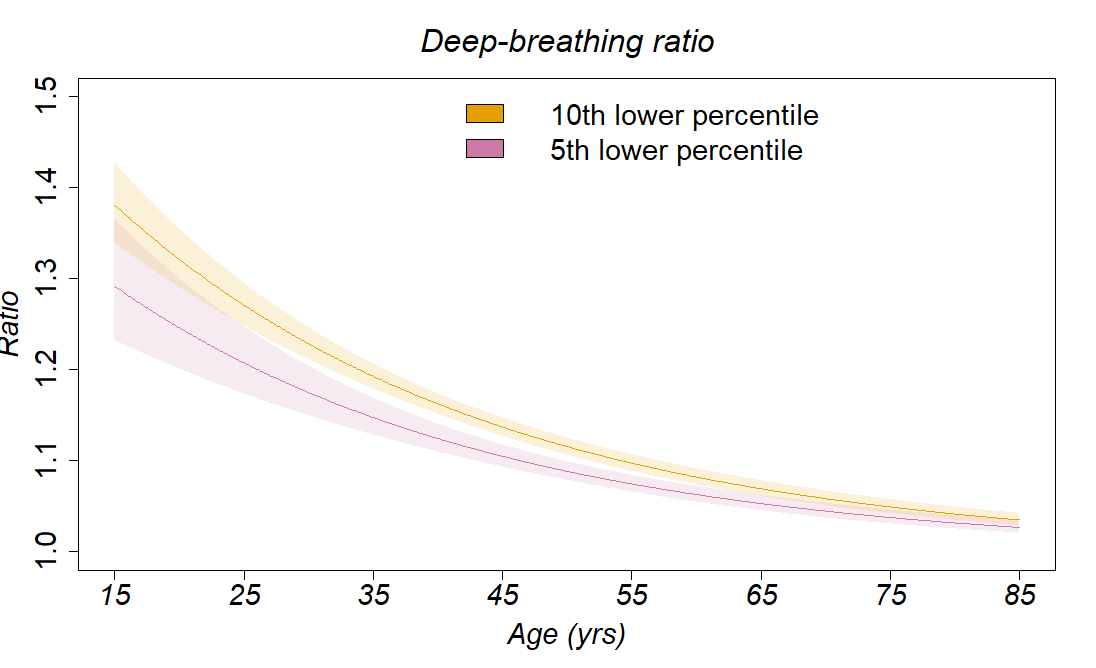


The 5^th^ lowest percentile:

exp(Est.) 2.5% 97.5% P
(Intercept) 0.487 0.358 0.661 0
AgeVisit 0.966 0.961 0.972 0

The 10^th^ lowest percentile:
 exp(Est.) 2.5% 97.5% P
(Intercept) 0.636 0.539 0.749 0
AgeVisit 0.966 0.963 0.970 0

### Valsalva manoeuvre:


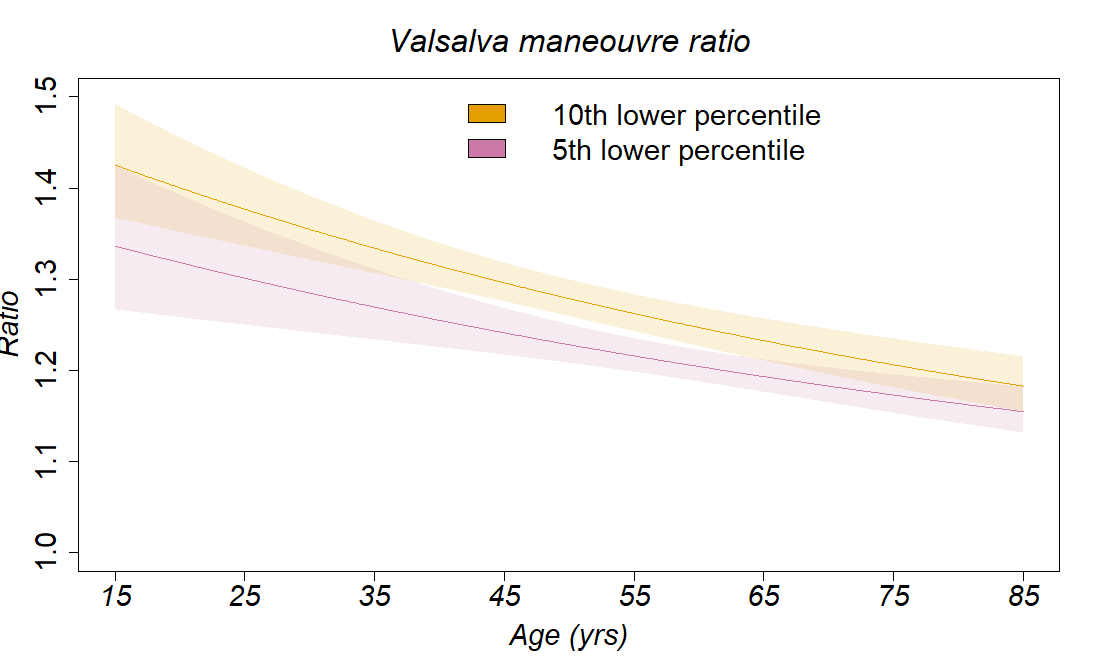


The 5^th^ lowest percentile:

exp(Est.) 2.5% 97.5% P
(Intercept) 0.397 0.293 0.539 0
AgeVisit 0.989 0.984 0.994 0

The 10^th^ lowest percentile:
 exp(Est.) 2.5% 97.5% P
(Intercept) 0.510 0.417 0.622 0
AgeVisit 0.988 0.984 0.992 0

## HRV (adjusted for resting heart rate):

### SDNN:


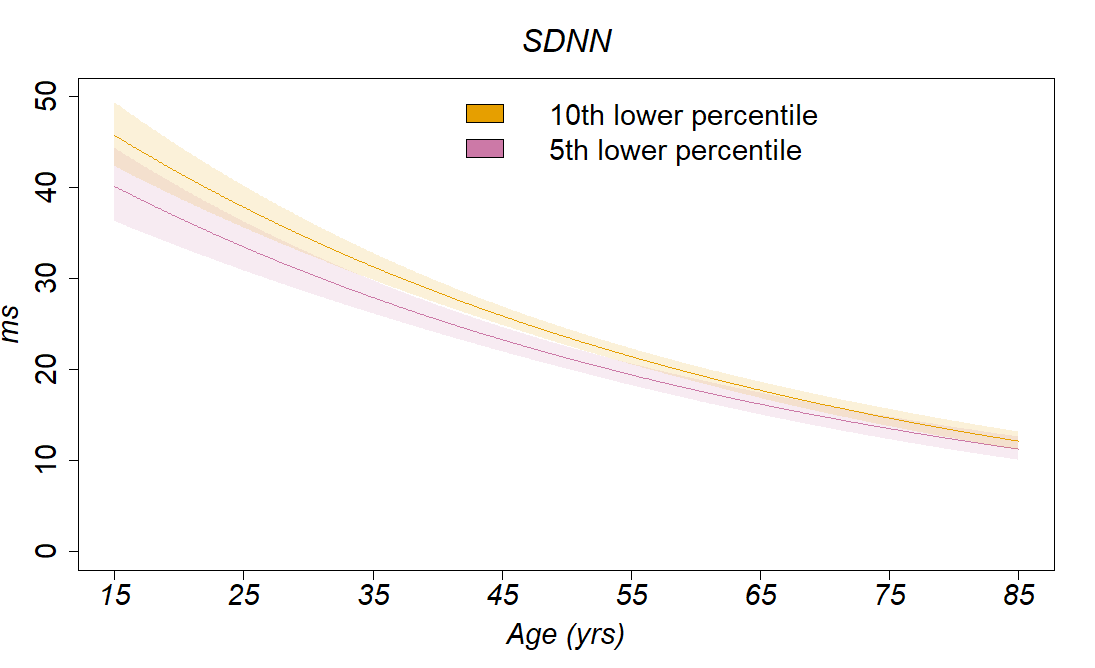


The 5^th^ lowest percentile:

exp(Est.) 2.5% 97.5% P
(Intercept) 229.119 162.129 323.789 0
AgeVisit 0.982 0.980 0.985 0
HR_BPM 0.978 0.974 0.983 0

The 10^th^ lowest percentile:
 exp(Est.) 2.5% 97.5% P
(Intercept) 296.103 222.992 393.184 0
AgeVisit 0.981 0.979 0.983 0
HR_BPM 0.977 0.973 0.980 0

### RMSSD:


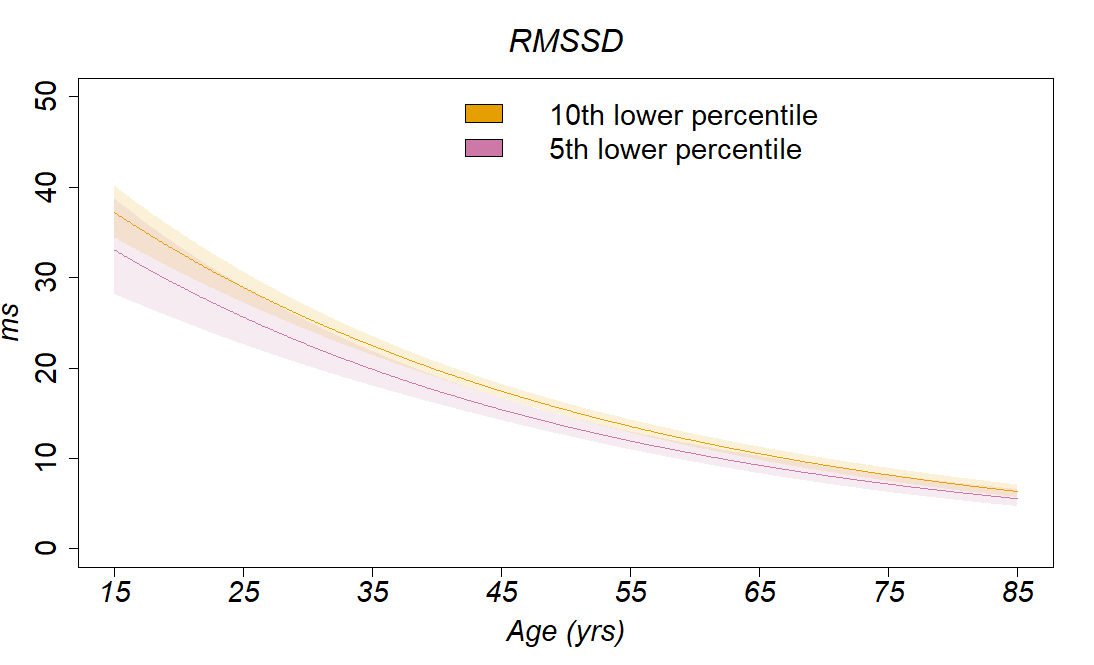


The 5^th^ lowest percentile:

exp(Est.) 2.5% 97.5% P
(Intercept) 639.129 348.133 1173.362 0
AgeVisit 0.975 0.971 0.979 0
HR_BPM 0.962 0.954 0.970 0

The 10^th^ lowest percentile:
 exp(Est.) 2.5% 97.5% P
(Intercept) 546.108 389.988 764.726 0
AgeVisit 0.975 0.973 0.977 0
HR_BPM 0.966 0.961 0.971 0

### HF power:


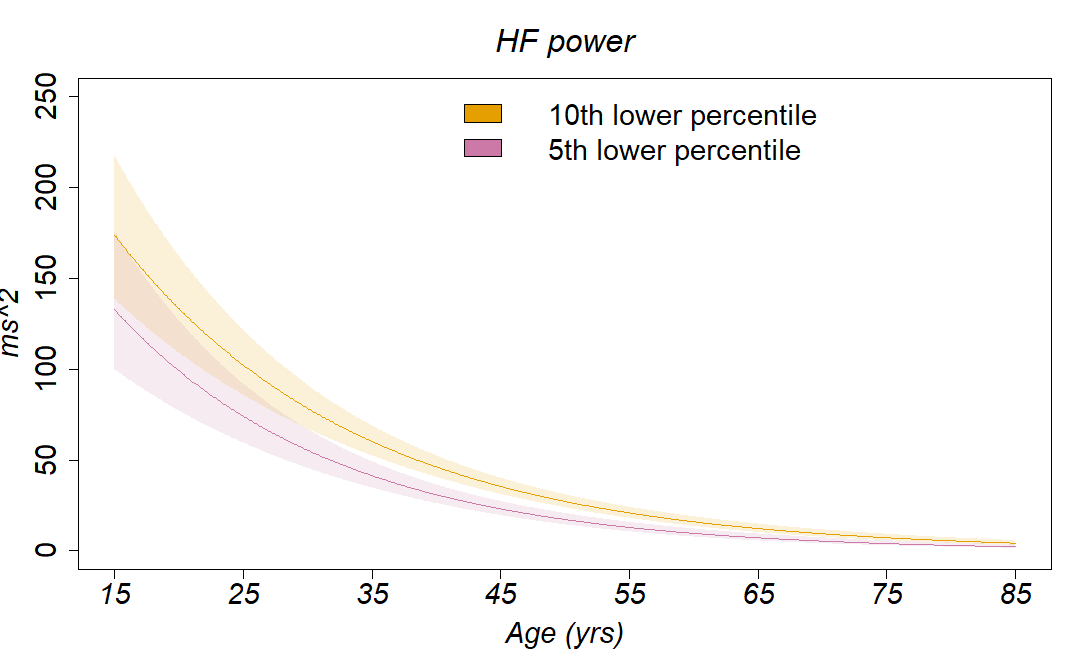


The 5^th^ lowest percentile:

exp(Est.) 2.5% 97.5% P
(Intercept) 27096.127 7906.014 92866.023 0
AgeVisit 0.943 0.935 0.952 0
HR_BPM 0.936 0.920 0.951 0

The 10^th^ lowest percentile:
 exp(Est.) 2.5% 97.5% P
(Intercept) 30145.647 11740.310 77405.110 0
AgeVisit 0.948 0.942 0.955 0
HR_BPM 0.937 0.925 0.949 0

### LF power:


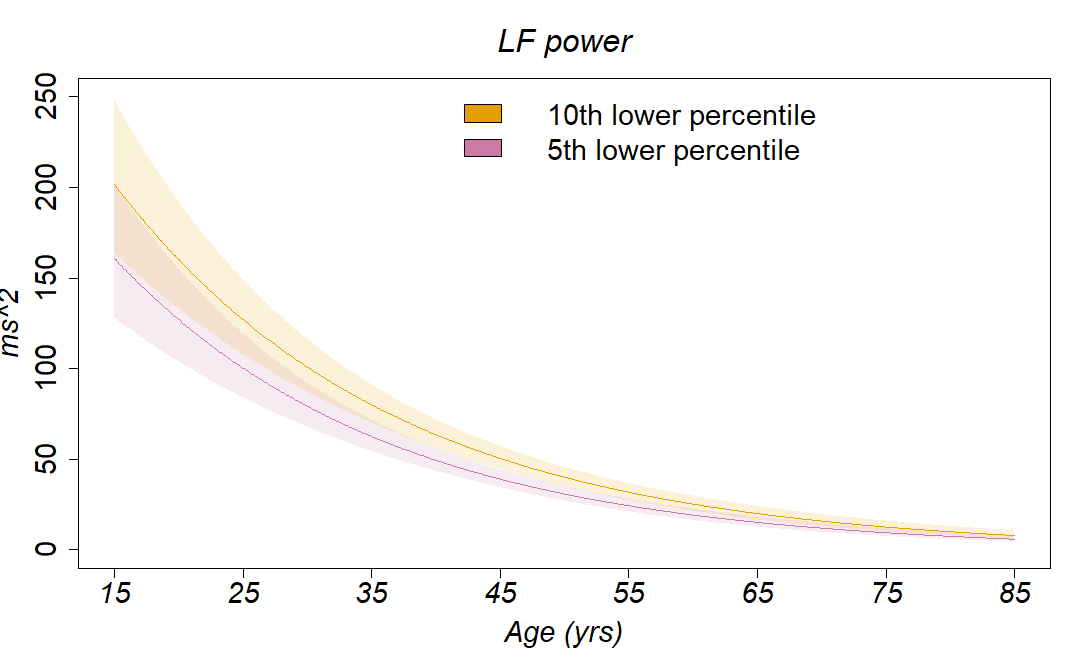


The 5^th^ lowest percentile:

exp(Est.) 2.5% 97.5% P
(Intercept) 2580.671 998.151 6672.196 0
AgeVisit 0.954 0.948 0.960 0
HR_BPM 0.970 0.957 0.982 0

The 10^th^ lowest percentile:
 exp(Est.) 2.5% 97.5% P
(Intercept) 3337.847 1409.349 7905.225 0
AgeVisit 0.955 0.949 0.961 0
HR_BPM 0.969 0.958 0.980 0

### Total power:


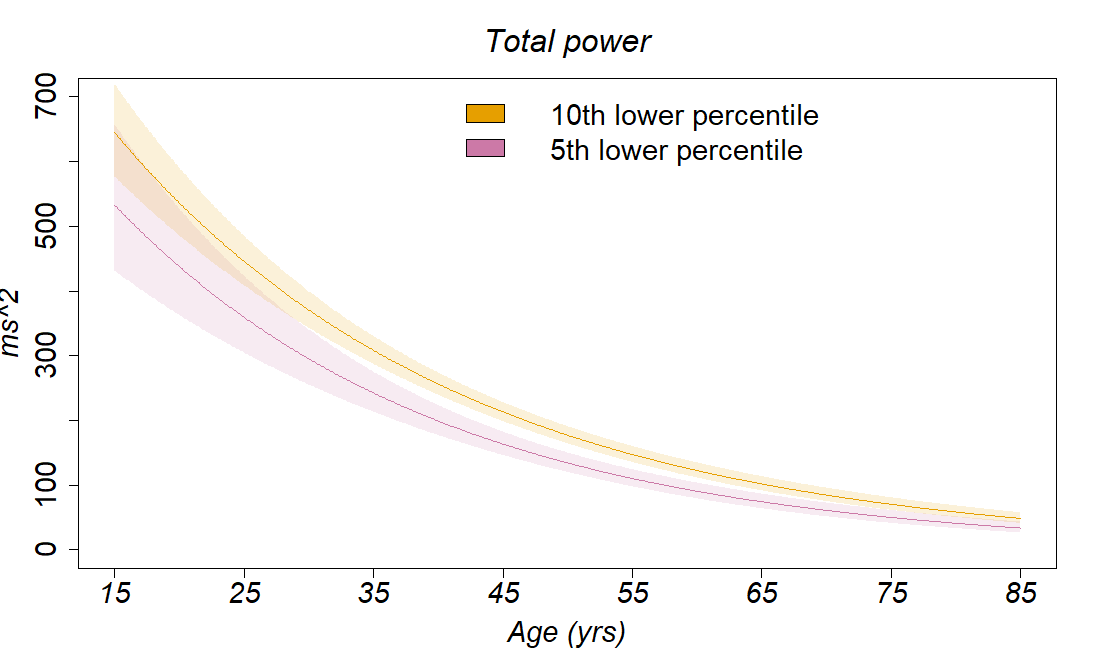


The 5^th^ lowest percentile:

exp(Est.) 2.5% 97.5% P
(Intercept) 11274.014 4894.882 25966.590 0
AgeVisit 0.961 0.956 0.967 0
HR_BPM 0.964 0.953 0.975 0

The 10^th^ lowest percentile:
 exp(Est.) 2.5% 97.5% P
(Intercept) 16638.061 10210.259 27112.444 0
AgeVisit 0.964 0.960 0.967 0
HR_BPM 0.961 0.954 0.967 0

## Frequency domain analyses during the CARTs:

### HF power during Lying to standing:


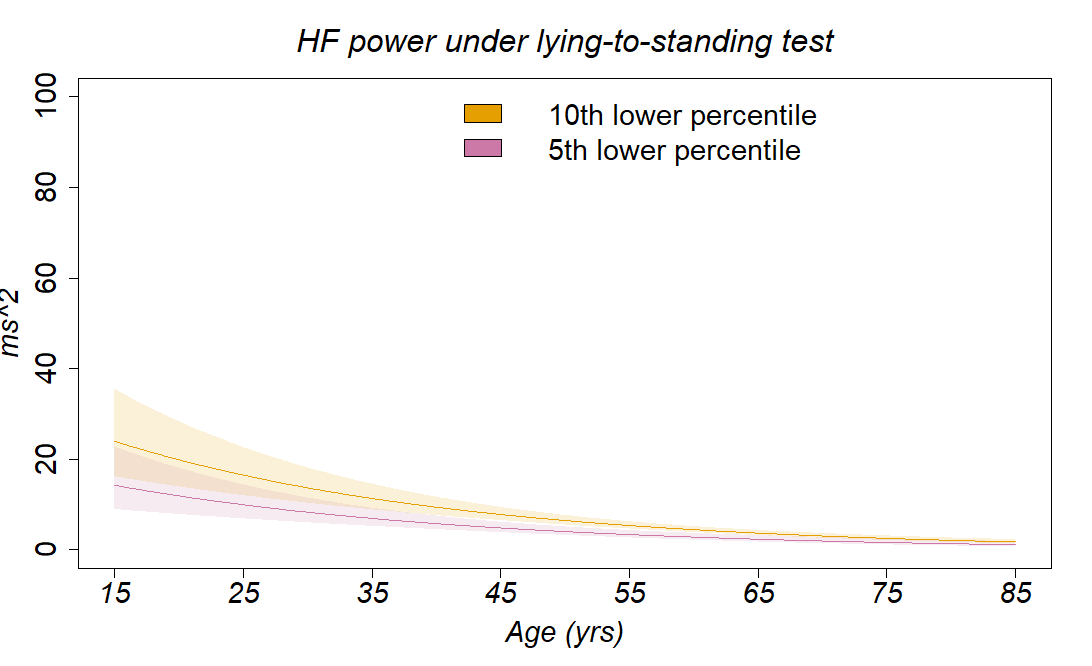


The 5^th^ lowest percentile:

exp(Est.) 2.5% 97.5% P
(Intercept) 686.230 119.758 3932.199 0
AgeVisit 0.965 0.953 0.976 0
HR_BPM 0.951 0.929 0.974 0

The 10^th^ lowest percentile:
 exp(Est.) 2.5% 97.5% P
(Intercept) 712.817 219.964 2309.958 0
AgeVisit 0.963 0.955 0.971 0
HR_BPM 0.959 0.944 0.973 0

### LF power during Lying to standing:


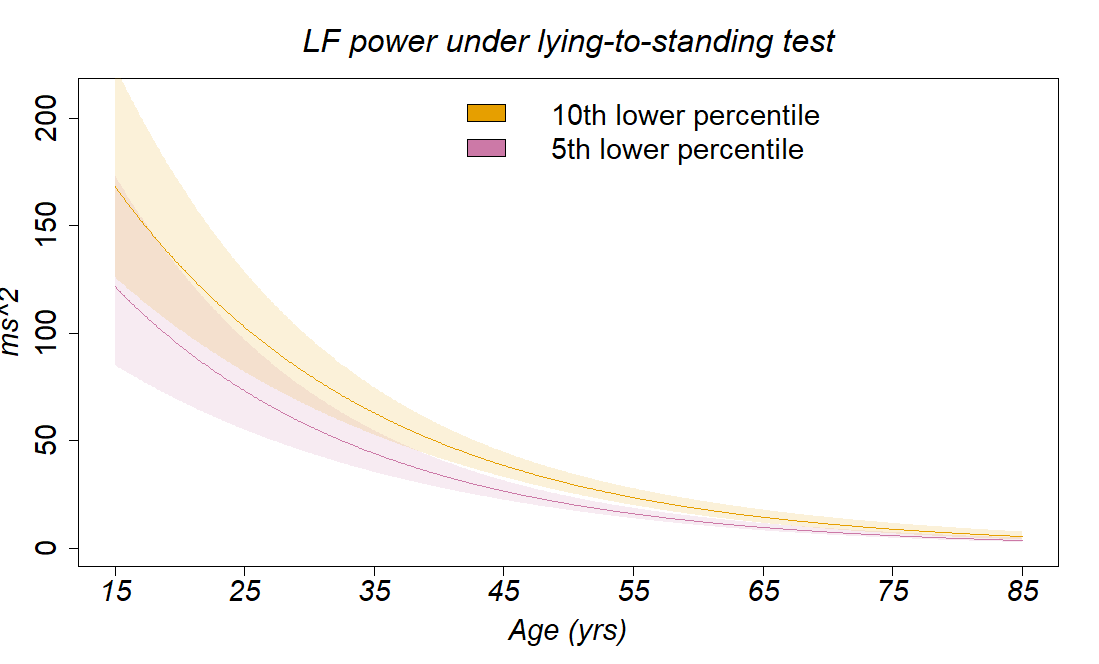


The 5^th^ lowest percentile:

exp(Est.) 2.5% 97.5% P
(Intercept) 7778.155 2313.369 26152.200 0
AgeVisit 0.950 0.943 0.958 0
HR_BPM 0.951 0.935 0.966 0

The 10^th^ lowest percentile:
 exp(Est.) 2.5% 97.5% P
(Intercept) 10032.913 3137.624 32081.392 0
AgeVisit 0.952 0.944 0.959 0
HR_BPM 0.951 0.936 0.967 0

### HF power during Deep breathing:


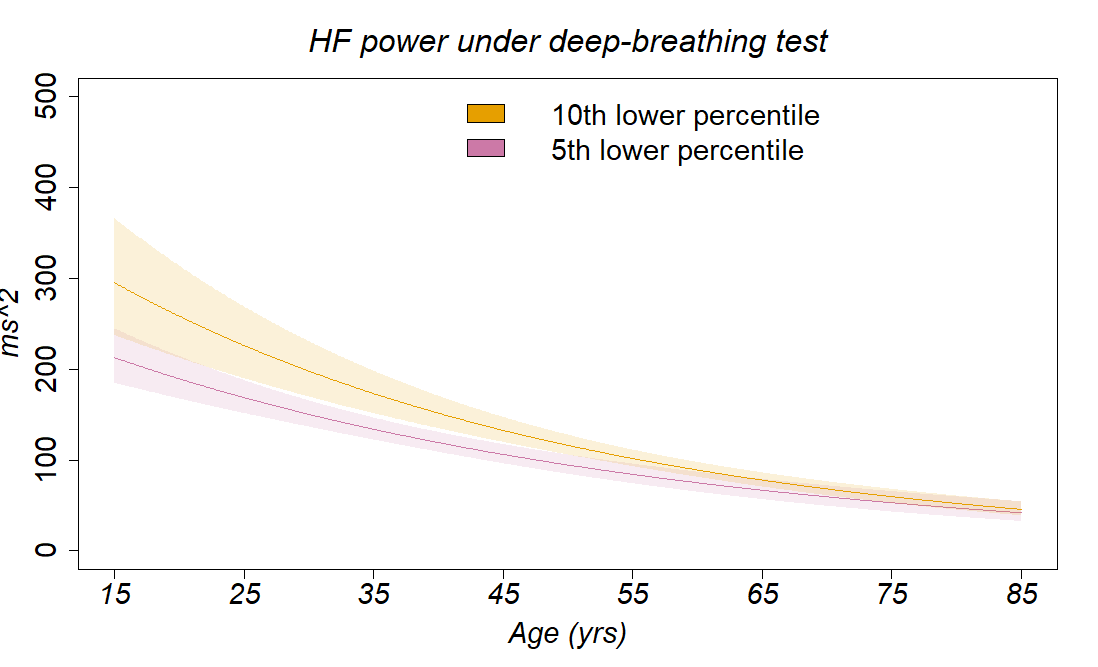


The 5^th^ percentile:

exp(Est.) 2.5% 97.5% P
(Intercept) 1940.664 980.001 3843.032 0
AgeVisit 0.977 0.972 0.982 0
HR_BPM 0.973 0.963 0.983 0

The 10^th^ percentile:
 exp(Est.) 2.5% 97.5% P
(Intercept) 2960.354 1485.144 5900.908 0
AgeVisit 0.974 0.969 0.978 0
HR_BPM 0.972 0.963 0.981 0

### LF power during Deep breathing:


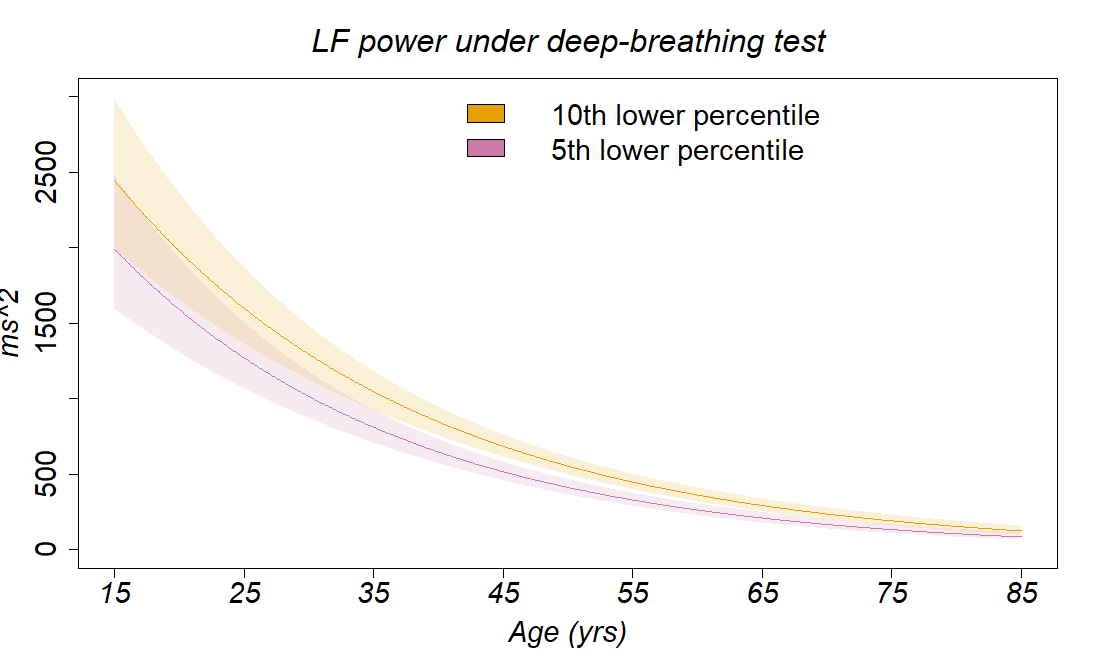


The 5^th^ lowest percentile:

exp(Est.) 2.5% 97.5% P
(Intercept) 20686.593 8548.276 50060.986 0
AgeVisit 0.956 0.950 0.962 0
HR_BPM 0.975 0.963 0.988 0

The 10^th^ lowest percentile:
 exp(Est.) 2.5% 97.5% P
(Intercept) 32087.626 14862.734 69274.992 0
AgeVisit 0.959 0.953 0.964 0
HR_BPM 0.971 0.962 0.981 0

### HF power during Valsalva manoeuvre:


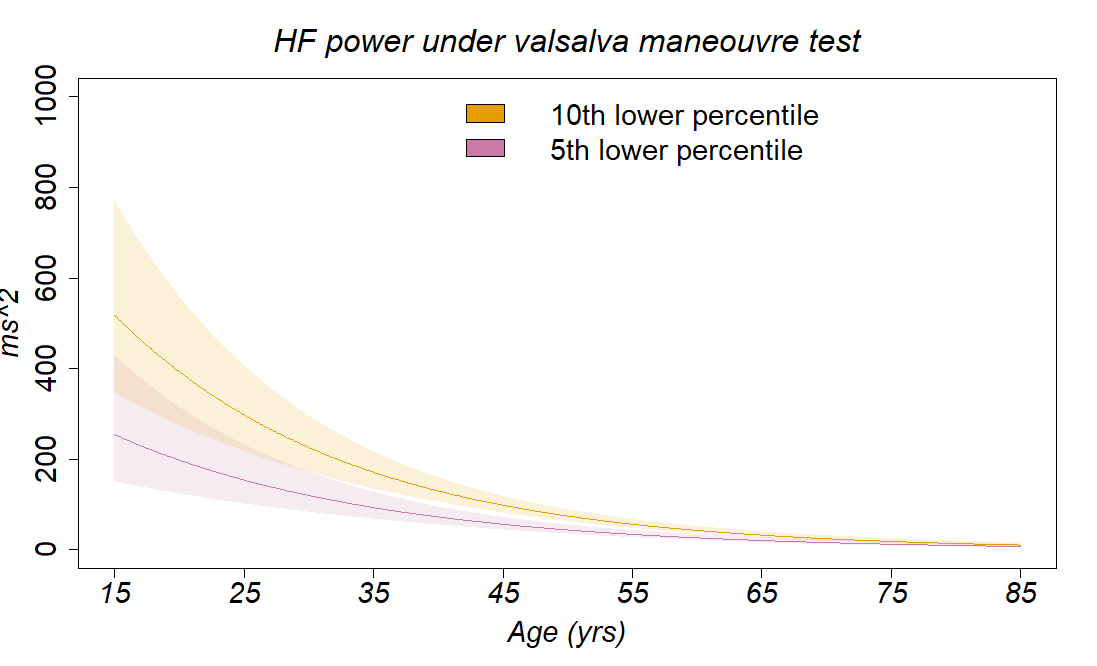


The 5^th^ lowest percentile:

exp(Est.) 2.5% 97.5% P
(Intercept) 4310.980 718.758 25856.489 0.000
AgeVisit 0.951 0.939 0.963 0.000
HR_BPM 0.969 0.947 0.992 0.009

The 10^th^ lowest percentile:
 exp(Est.) 2.5% 97.5% P
(Intercept) 13750.787 3424.808 55210.138 0
AgeVisit 0.946 0.937 0.956 0
HR_BPM 0.964 0.947 0.982 0

### LF power during Valsalva manoeuvre:


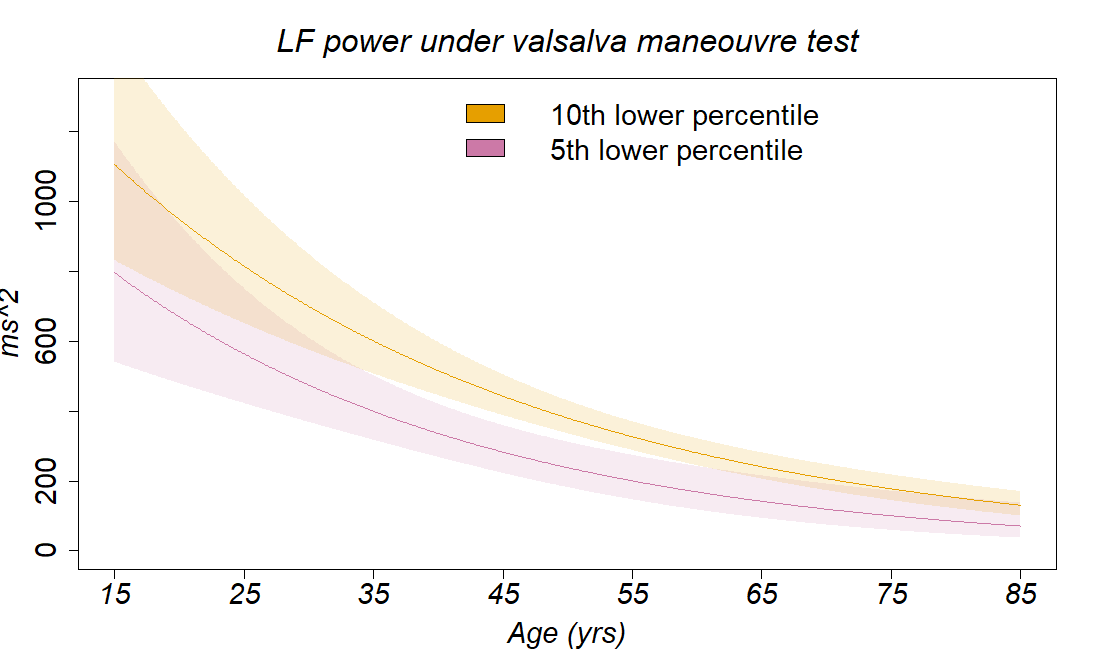


The 5^th^ lowest percentile:

exp(Est.) 2.5% 97.5% P
(Intercept) 6611.818 1038.773 42084.381 0.000
AgeVisit 0.966 0.953 0.979 0.000
HR_BPM 0.976 0.952 1.002 0.067

The 10^th^ lowest percentile:
 exp(Est.) 2.5% 97.5% P
(Intercept) 13387.61 4925.039 36391.208 0
AgeVisit 0.97 0.963 0.977 0
HR_BPM 0.97 0.957 0.983 0

# Adjustment for age and sex

We further adjusted for sex in the quantile regression models at the 5^th^ percentile. Estimates and p-values marked with a * were still significant after controlling for multiple testing (The Benjamini Hochberg procedure). The results are from log-transformed models. The estimates have been back-transformed.

**CARTs:**

Lying to standing:

exp(Est.) 2.5% 97.5% P
(Intercept) 0.251 0.171 0.369 0.00
AgeVisit 0.972 0.966 0.978 0.00
sexMale 1.108 0.869 1.412 0.41

Deep breathing:

exp(Est.) 2.5% 97.5% P
(Intercept) 0.599 0.475 0.755 0
AgeVisit 0.967 0.963 0.971 0
sexMale 0.662 0.557 0.787 0*

Valsalva manoeuvre:

exp(Est.) 2.5% 97.5% P
(Intercept) 0.399 0.285 0.560 0.00
AgeVisit 0.989 0.984 0.995 0.00
sexMale 0.977 0.793 1.205 0.83

**HRV outcomes (additionally adjusted for HR)**

SDNN:

exp(Est.) 2.5% 97.5% P
(Intercept) 232.251 158.927 339.403 0.000
AgeVisit 0.982 0.980 0.985 0.000
HR_BPM 0.979 0.974 0.983 0.000
sexMale 0.963 0.868 1.068 0.476

RMSSD:

exp(Est.) 2.5% 97.5% P
(Intercept) 472.646 312.895 713.960 0.000
AgeVisit 0.976 0.973 0.979 0.000
HR_BPM 0.967 0.962 0.973 0.000
sexMale 0.840 0.751 0.939 0.002*

HF power:

exp(Est.) 2.5% 97.5% P
(Intercept) 24138.508 9570.244 60883.254 0
AgeVisit 0.951 0.946 0.957 0
HR_BPM 0.938 0.927 0.950 0
sexMale 0.462 0.362 0.590 0*

LF power:

exp(Est.) 2.5% 97.5% P
(Intercept) 1951.733 875.223 4352.335 0.000
AgeVisit 0.954 0.949 0.959 0.000
HR_BPM 0.972 0.962 0.982 0.000
sexMale 1.190 0.963 1.470 0.107

Total power:

exp(Est.) 2.5% 97.5% P
(Intercept) 10015.037 4901.098 20464.998 0.000
AgeVisit 0.964 0.960 0.969 0.000
HR_BPM 0.965 0.956 0.974 0.000
sexMale 0.838 0.695 1.011 0.065

HF power during lying to standing:

exp(Est.) 2.5% 97.5% P
(Intercept) 359.012 64.422 2000.702 0.000
AgeVisit 0.971 0.960 0.982 0.000
HR_BPM 0.960 0.937 0.983 0.001
sexMale 0.512 0.331 0.791 0.003*

LF power during lying to standing:

exp(Est.) 2.5% 97.5% P
(Intercept) 8362.293 3077.505 22722.289 0.000
AgeVisit 0.949 0.943 0.956 0.000
HR_BPM 0.950 0.937 0.963 0.000
sexMale 1.174 0.888 1.551 0.261

HF power during deep breathing:

exp(Est.) 2.5% 97.5% P
(Intercept) 2067.348 1199.966 3561.707 0.000
AgeVisit 0.978 0.975 0.980 0.000
HR_BPM 0.972 0.964 0.979 0.000
sexMale 0.875 0.751 1.020 0.088

LF power during deep breathing:

exp(Est.) 2.5% 97.5% P
(Intercept) 39663.437 15475.310 101657.948 0.000
AgeVisit 0.957 0.951 0.963 0.000
HR_BPM 0.968 0.956 0.980 0.000
sexMale 0.708 0.561 0.892 0.003*

HF power during Valsalva manoeuvre:

exp(Est.) 2.5% 97.5% P
(Intercept) 9697.278 2472.099 38039.415 0.000
AgeVisit 0.951 0.941 0.961 0.000
HR_BPM 0.964 0.948 0.979 0.000
sexMale 0.544 0.361 0.819 0.004*

LF power during Valsalva manoeuvre:

exp(Est.) 2.5% 97.5% P
(Intercept) 9665.830 1438.218 64961.121 0.000
AgeVisit 0.961 0.949 0.974 0.000
HR_BPM 0.972 0.947 0.997 0.028
sexMale 1.461 0.918 2.327 0.110

# Final models stratified by sex

We have chosen to stratify the study population by sex and again assess the association between age and all outcomes at the lowest 5^th^ and 10^th^ percentile. The estimates displayed at the y-axis are back-transformed.

## Females

## CARTs

### Lying-to-standing:


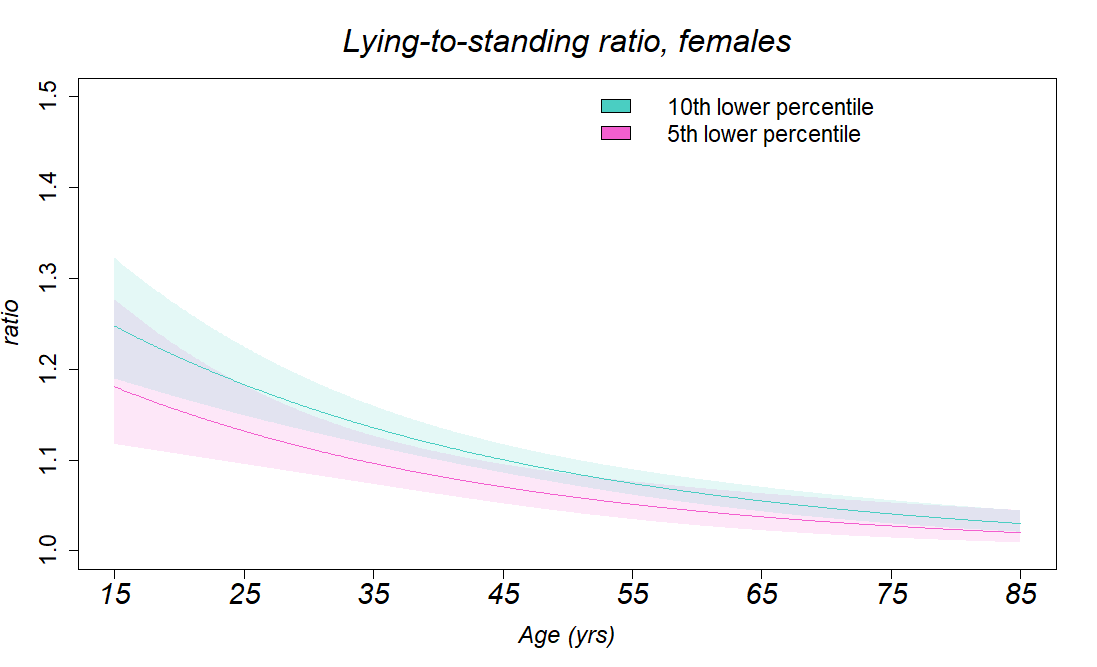


The lowest 5^th^ percentile:

exp(Est.) 2.5% 97.5% P
(Intercept) 0.289 0.155 0.540 0
AgeVisit 0.969 0.954 0.984 0

The lowest 10^th^ percentile:
 exp(Est.) 2.5% 97.5% P
(Intercept) 0.388 0.268 0.562 0
AgeVisit 0.970 0.963 0.978 0

### Deep breathing:


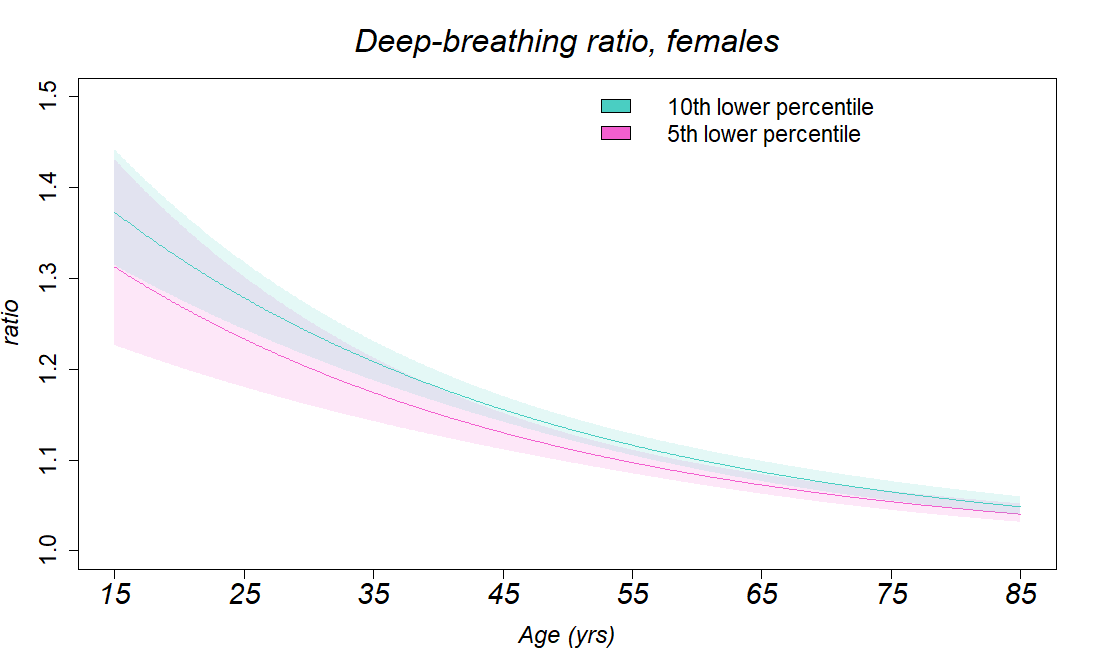


The lowest 5^th^ percentile:

exp(Est.) 2.5% 97.5% P
(Intercept) 0.484 0.318 0.738 0.001
AgeVisit 0.971 0.964 0.978 0.000

The lowest 10^th^ percentile:
 exp(Est.) 2.5% 97.5% P
(Intercept) 0.577 0.458 0.728 0
AgeVisit 0.971 0.967 0.976 0

### Valsalva manoeuvre:


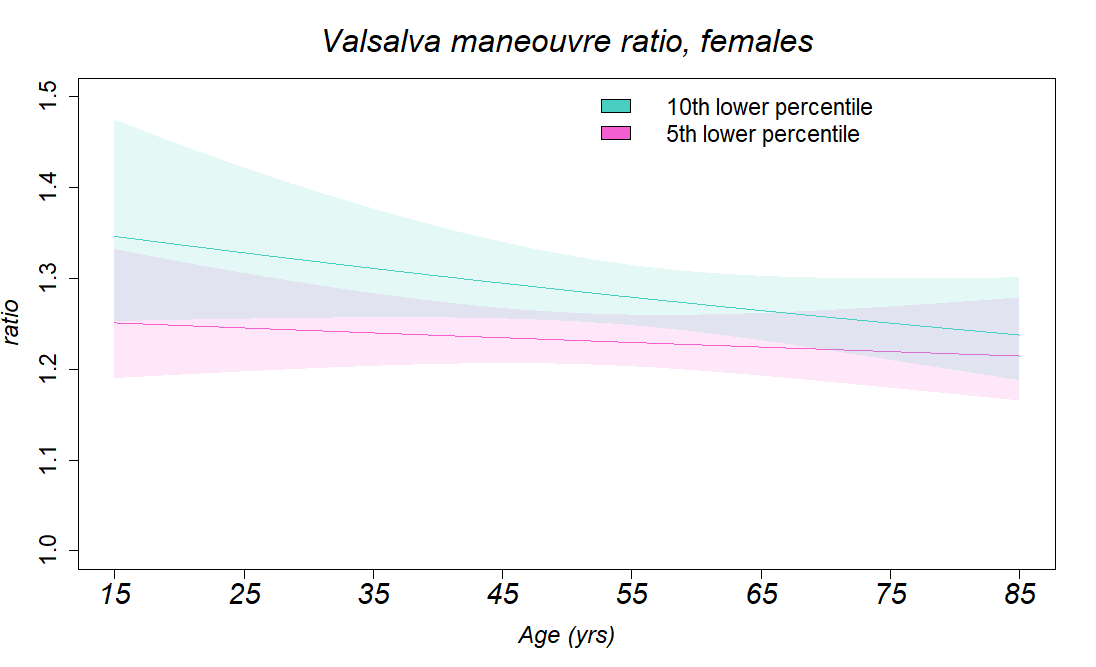


The lowest 5^th^ percentile:

exp(Est.) 2.5% 97.5% P
(Intercept) 0.260 0.179 0.377 0.000
AgeVisit 0.998 0.991 1.005 0.522

The lowest 10^th^ percentile:
 exp(Est.) 2.5% 97.5% P
(Intercept) 0.376 0.248 0.568 0.000
AgeVisit 0.995 0.988 1.002 0.136

## HRV (adjusted for resting heart rate):

### SDNN:


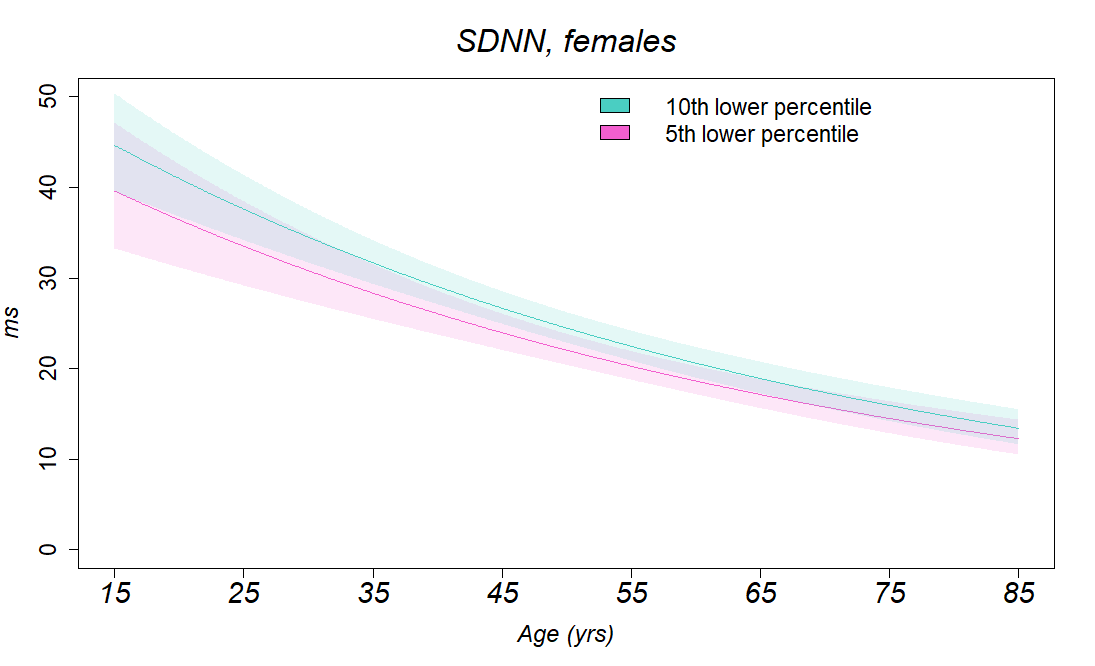


The lowest 5^th^ percentile:

exp(Est.) 2.5% 97.5% P
(Intercept) 285.487 151.818 536.845 0
AgeVisit 0.983 0.979 0.988 0
HR_BPM 0.975 0.966 0.983 0

The lowest 10^th^ percentile:
 exp(Est.) 2.5% 97.5% P
(Intercept) 315.829 194.554 512.702 0
AgeVisit 0.983 0.980 0.986 0
HR_BPM 0.975 0.969 0.981 0

### RMSSD:


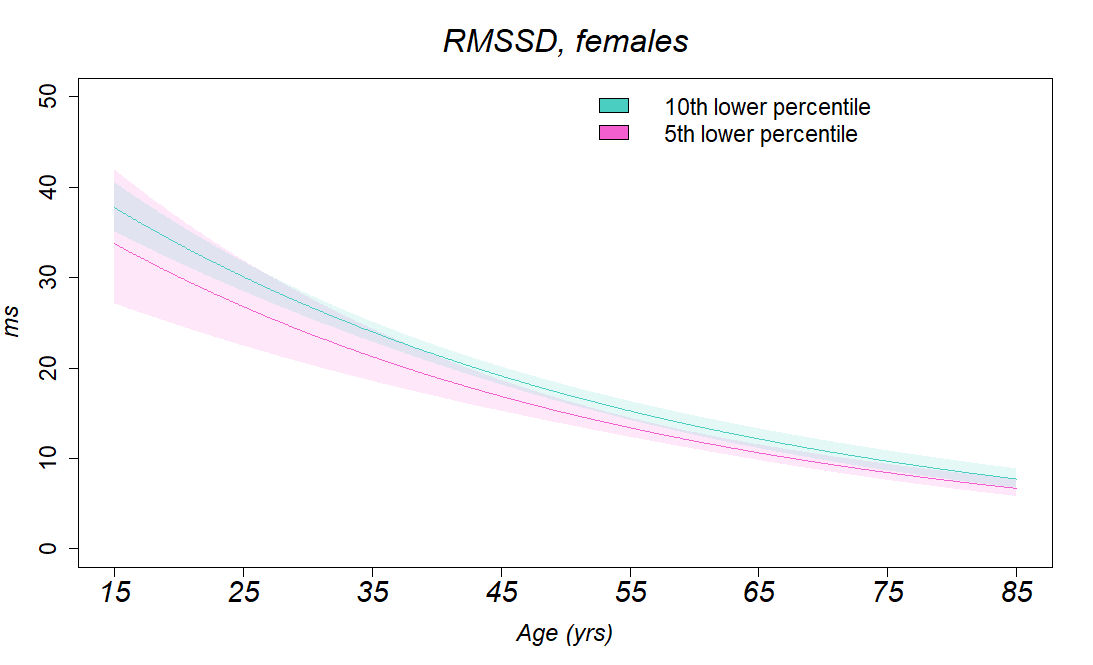


The lowest 5^th^ percentile:

exp(Est.) 2.5% 97.5% P
(Intercept) 475.063 244.637 922.529 0
AgeVisit 0.977 0.973 0.982 0
HR_BPM 0.966 0.957 0.975 0

The lowest 10^th^ percentile:
 exp(Est.) 2.5% 97.5% P
(Intercept) 535.640 367.021 781.728 0
AgeVisit 0.978 0.975 0.980 0
HR_BPM 0.966 0.961 0.971 0

### HF power:


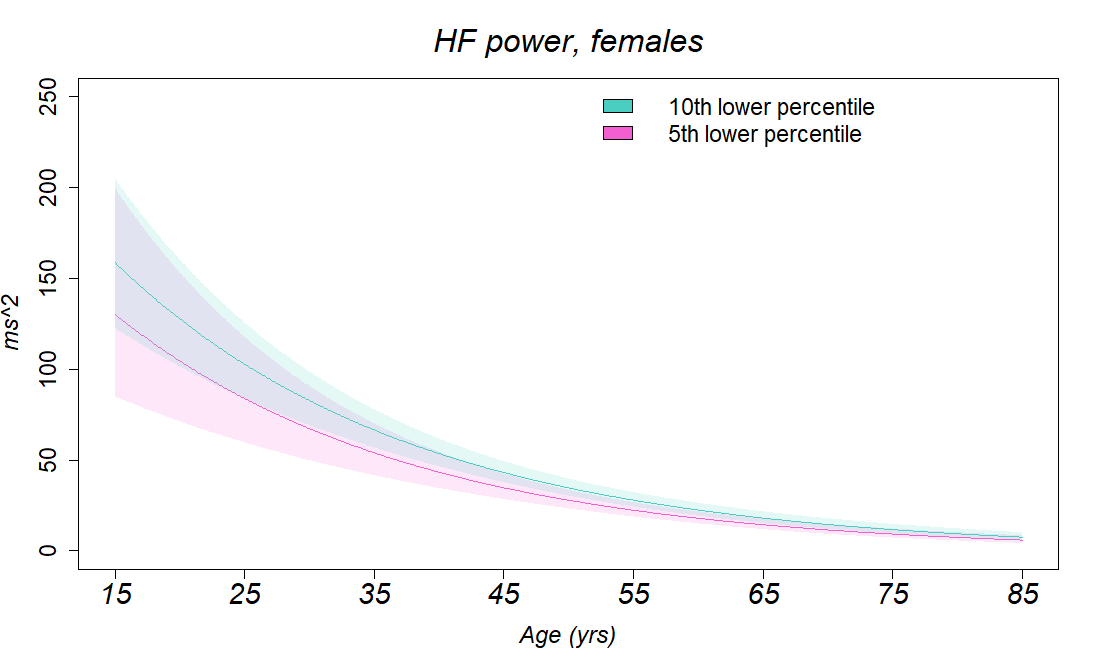


exp(Est.) 2.5% 97.5% P

The lowest 5^th^ percentile:
(Intercept) 30827.284 7142.450 133052.593 0
AgeVisit 0.957 0.948 0.966 0
HR_BPM 0.931 0.912 0.950 0

The lowest 10^th^ percentile:
 exp(Est.) 2.5% 97.5% P
(Intercept) 25609.536 9325.345 70329.662 0
AgeVisit 0.957 0.951 0.964 0
HR_BPM 0.936 0.923 0.949 0

### LF power:


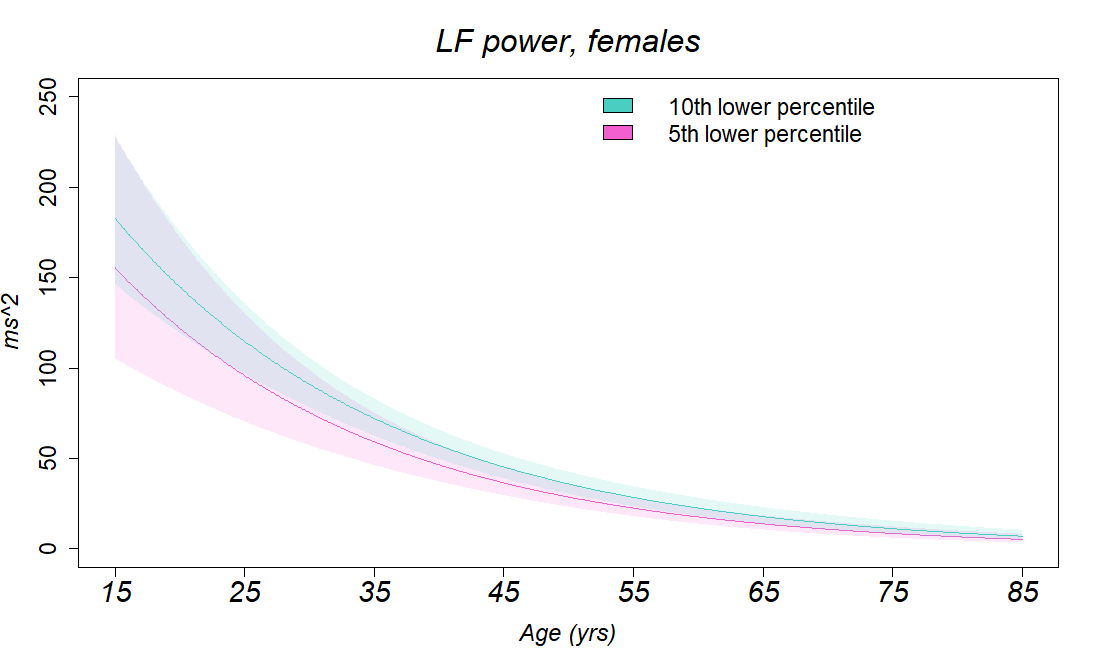


The lowest 5^th^ percentile:

exp(Est.) 2.5% 97.5% P
(Intercept) 5081.575 1114.644 23166.495 0
AgeVisit 0.953 0.943 0.963 0
HR_BPM 0.960 0.940 0.979 0

The lowest 10^th^ percentile:
 exp(Est.) 2.5% 97.5% P
(Intercept) 3694.611 1231.354 11085.480 0
AgeVisit 0.955 0.947 0.962 0
HR_BPM 0.966 0.952 0.981 0

### Total power:


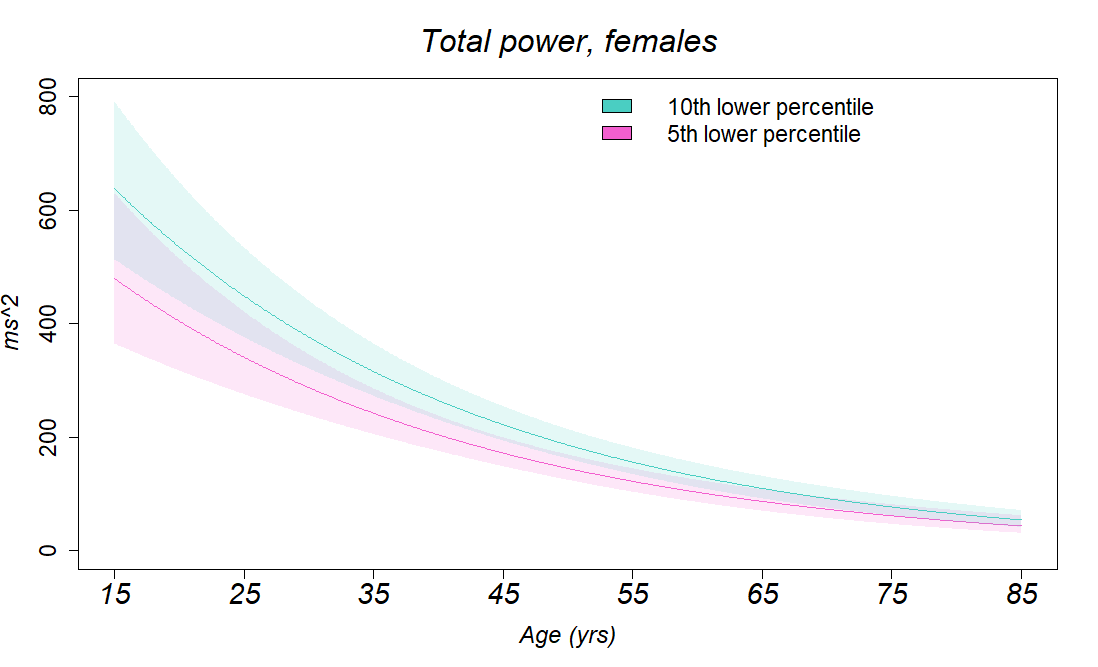


The lowest 5^th^ percentile:

exp(Est.) 2.5% 97.5% P
(Intercept) 10929.588 3152.781 37889.051 0
AgeVisit 0.966 0.959 0.974 0
HR_BPM 0.962 0.945 0.979 0

The lowest 10^th^ percentile:
 exp(Est.) 2.5% 97.5% P
(Intercept) 18334.000 8001.390 42009.643 0
AgeVisit 0.965 0.960 0.971 0
HR_BPM 0.959 0.949 0.969 0

## Frequency domain analyses during the CARTs:

### HF power during Lying-to-standing:


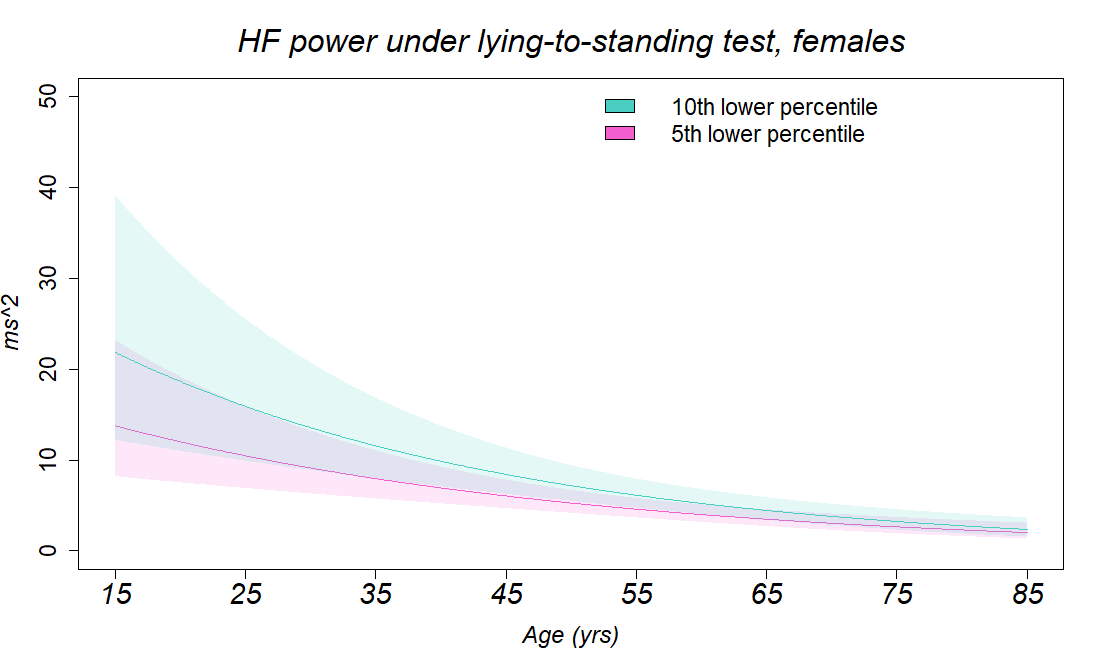


The lowest 5^th^ percentile:

exp(Est.) 2.5% 97.5% P
(Intercept) 223.949 41.291 1214.621 0.000
AgeVisit 0.973 0.962 0.984 0.000
HR_BPM 0.965 0.942 0.989 0.004

The lowest 10^th^ percentile:
 exp(Est.) 2.5% 97.5% P
(Intercept) 299.058 57.800 1547.331 0.000
AgeVisit 0.969 0.957 0.981 0.000
HR_BPM 0.969 0.948 0.989 0.003

### LF power during Lying-to-standing:


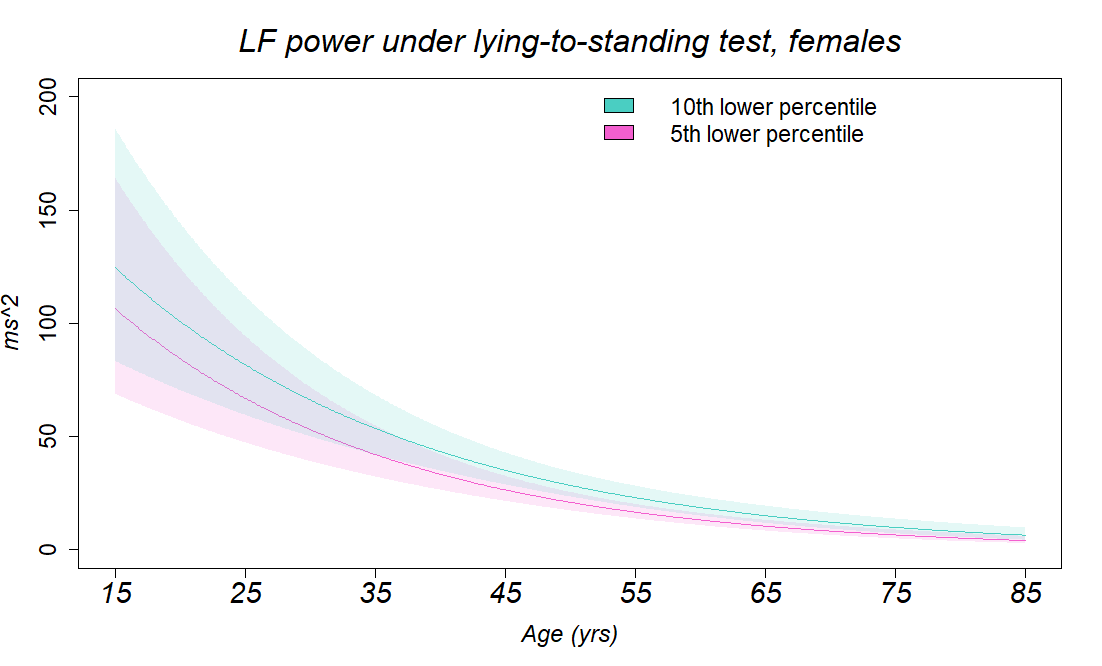


The lowest 5^th^ percentile:

exp(Est.) 2.5% 97.5% P
(Intercept) 9708.988 2051.591 45946.996 0
AgeVisit 0.955 0.945 0.965 0
HR_BPM 0.945 0.924 0.965 0

The lowest 10^th^ percentile:
 exp(Est.) 2.5% 97.5% P
(Intercept) 11047.011 2359.473 51721.904 0
AgeVisit 0.959 0.949 0.968 0
HR_BPM 0.944 0.924 0.965 0

### HF power during Deep-breathing:


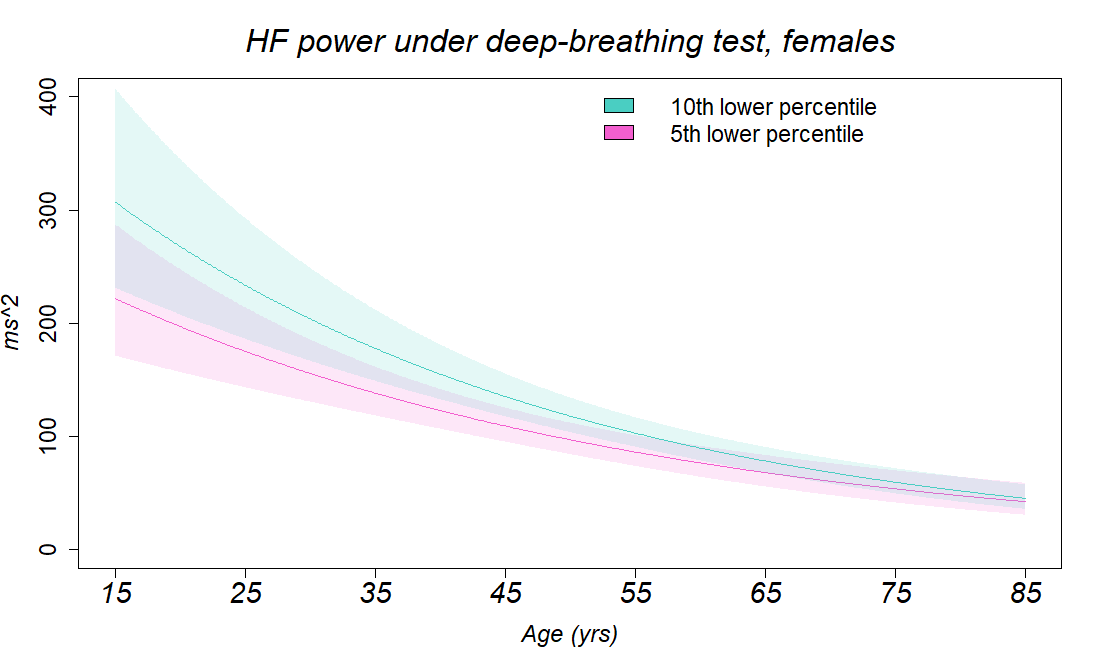


The lowest 5^th^ percentile:

exp(Est.) 2.5% 97.5% P
(Intercept) 2276.688 742.063 6985.004 0
AgeVisit 0.977 0.970 0.984 0
HR_BPM 0.971 0.956 0.986 0

The lowest 10^th^ percentile:
 exp(Est.) 2.5% 97.5% P
(Intercept) 2599.321 1008.780 6697.661 0
AgeVisit 0.973 0.967 0.979 0
HR_BPM 0.975 0.962 0.987 0

### LF power during Deep-breathing:


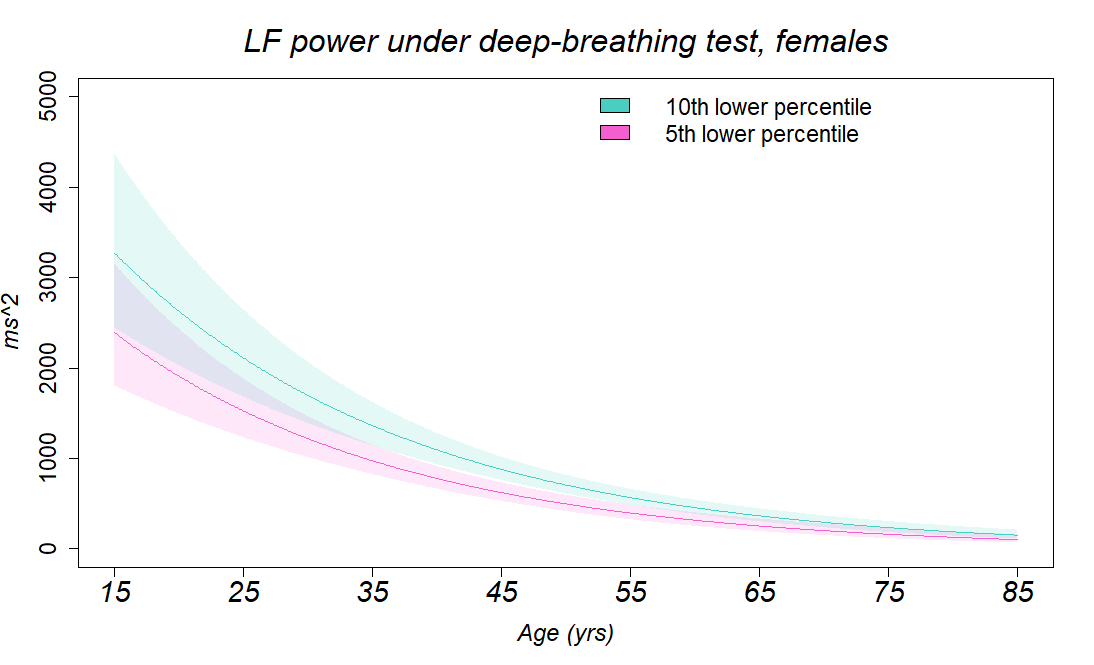


The lowest 5^th^ percentile:

exp(Est.) 2.5% 97.5% P
(Intercept) 55785.416 13454.578 231297.674 0
AgeVisit 0.956 0.948 0.965 0
HR_BPM 0.964 0.944 0.984 0

The lowest 10^th^ percentile:
 exp(Est.) 2.5% 97.5% P
(Intercept) 58644.364 18279.877 188139.203 0
AgeVisit 0.957 0.950 0.964 0
HR_BPM 0.967 0.952 0.983 0

### HF power during Valsalva manoeuvre:


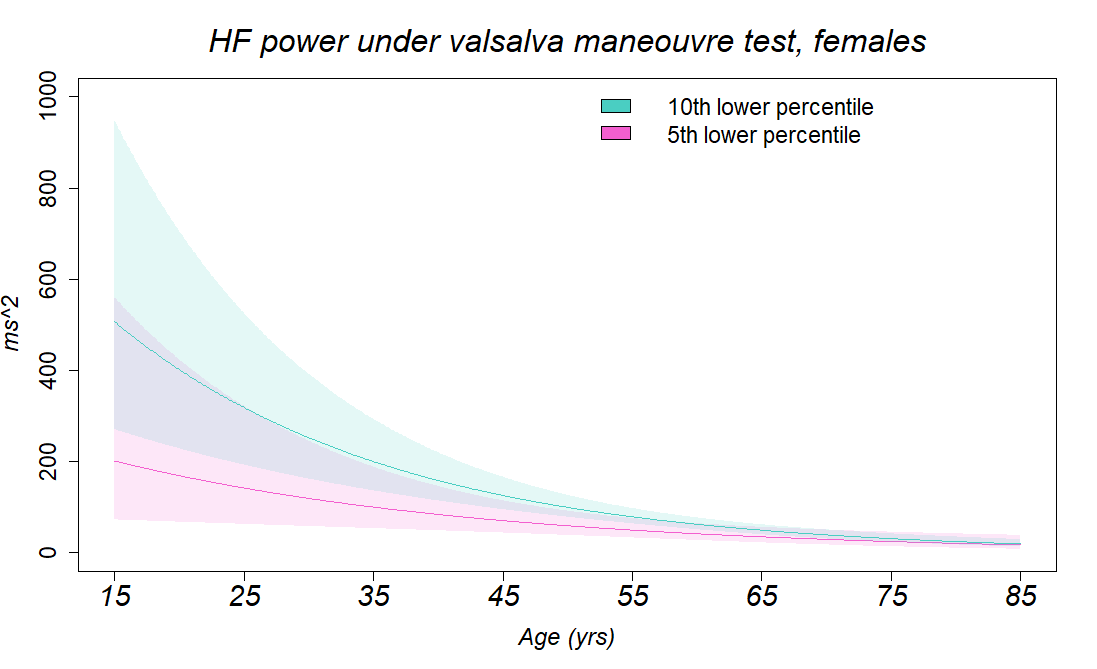


The lowest 5^th^ percentile:

exp(Est.) 2.5% 97.5% P
(Intercept) 5237.005 272.009 100828.448 0.000
AgeVisit 0.966 0.944 0.988 0.003
HR_BPM 0.960 0.925 0.996 0.032

The lowest 10^th^ percentile:
 exp(Est.) 2.5% 97.5% P
(Intercept) 8303.962 1234.279 55867.233 0.000
AgeVisit 0.955 0.942 0.967 0.000
HR_BPM 0.969 0.945 0.993 0.013

### LF power during Valsalva manoeuvre:


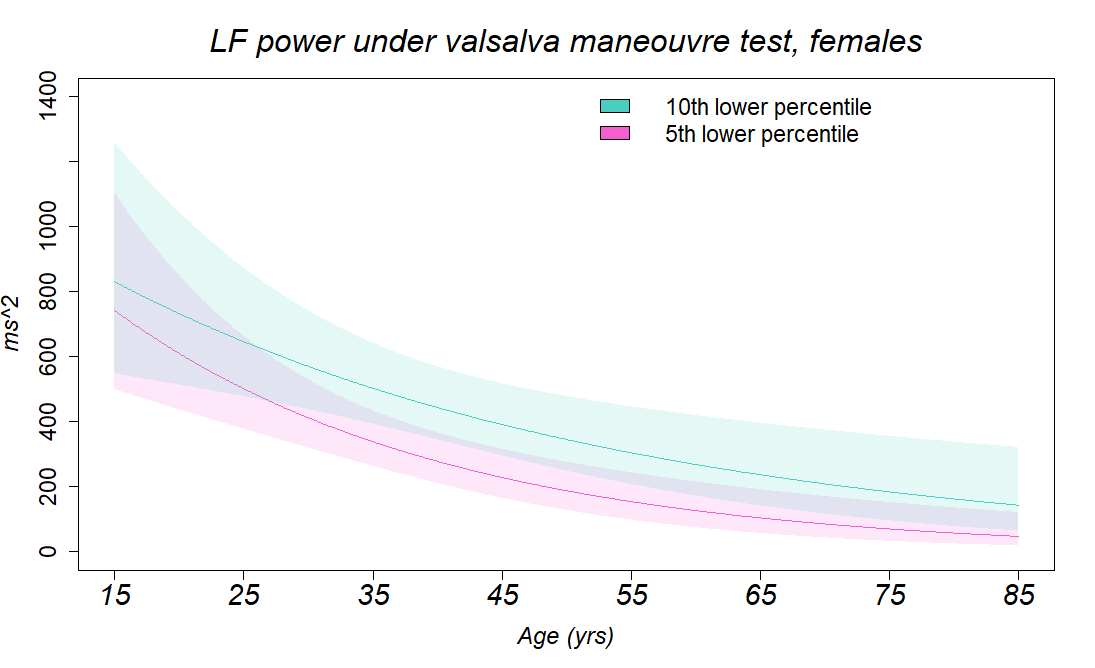


The lowest 5th percentile:

exp(Est.) 2.5% 97.5% P
(Intercept) 11406.083 977.414 133104.971 0.000
AgeVisit 0.962 0.945 0.978 0.000
HR_BPM 0.969 0.937 1.002 0.062

The lowest 10^th^ percentile:
 exp(Est.) 2.5% 97.5% P
(Intercept) 5382.146 535.285 54116.052 0.000
AgeVisit 0.975 0.960 0.991 0.002
HR_BPM 0.978 0.948 1.009 0.166

## Males

### CARTs

### Lying-to-standing:


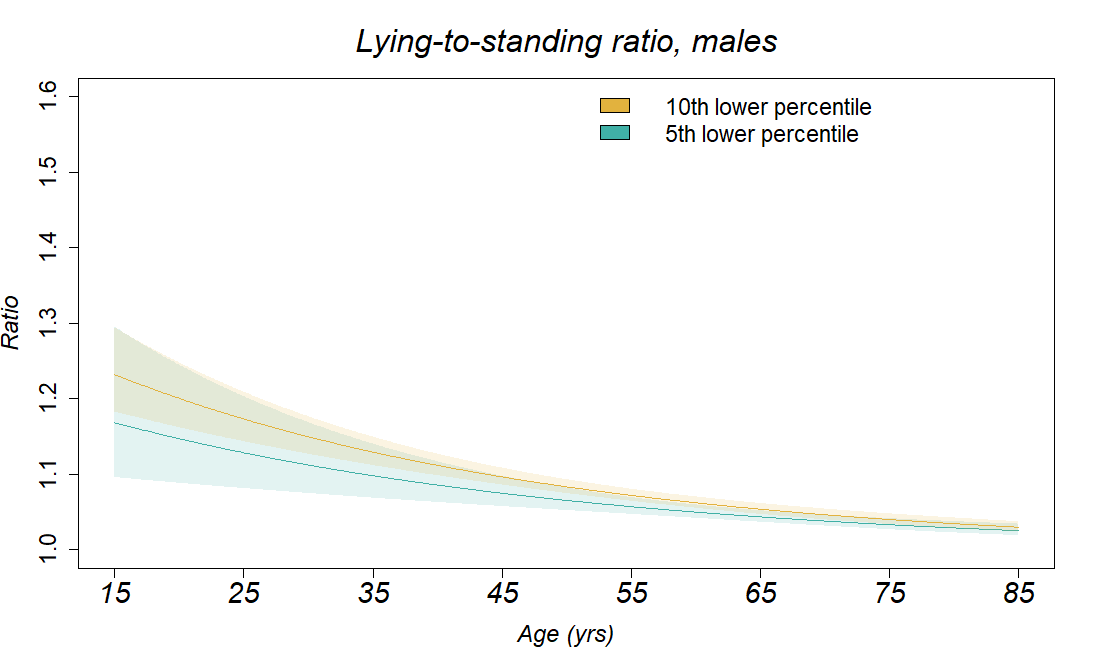


The lowest 5^th^ percentile:

exp(Est.) 2.5% 97.5% P
(Intercept) 0.252 0.123 0.519 0
AgeVisit 0.973 0.963 0.984 0

The lowest 10^th^ percentile:
 exp(Est.) 2.5% 97.5% P
(Intercept) 0.360 0.262 0.494 0
AgeVisit 0.971 0.966 0.977 0

### Deep-breathing:


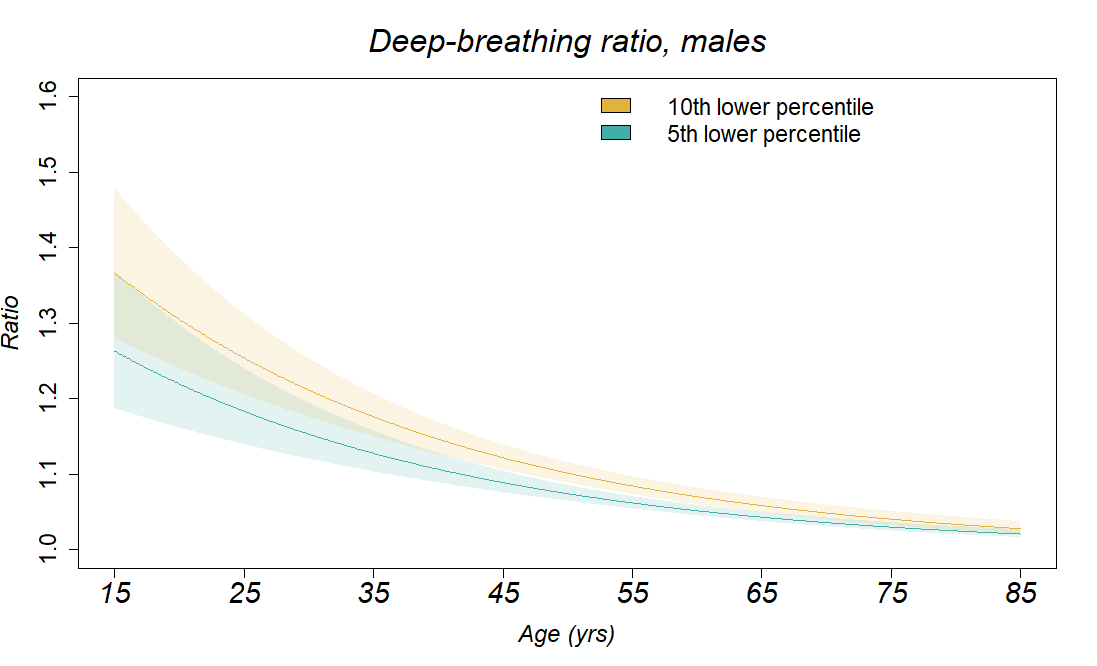


The lowest 5th percentile:

exp(Est.) 2.5% 97.5% P
(Intercept) 0.452 0.290 0.704 0
AgeVisit 0.965 0.957 0.972 0

The lowest 10th percentile:
 exp(Est.) 2.5% 97.5% P
(Intercept) 0.635 0.443 0.911 0.014
AgeVisit 0.964 0.957 0.971 0.000

### Valsalva manoeuvre:


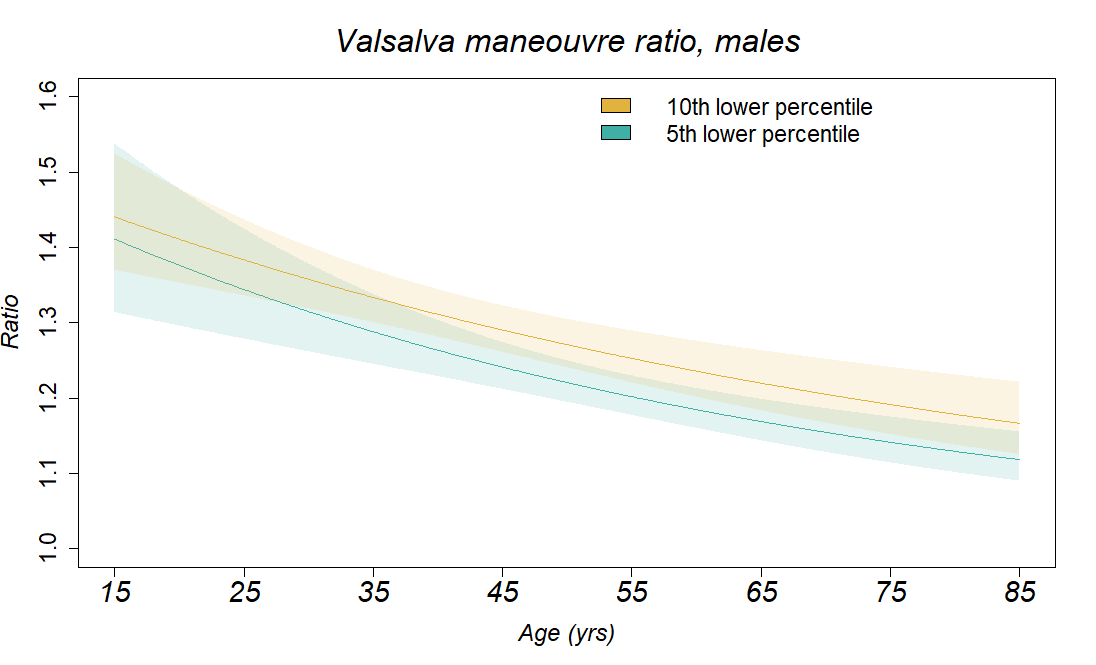


The lowest 5^th^ percentile:

exp(Est.) 2.5% 97.5% P
(Intercept) 0.537 0.373 0.771 0.001
AgeVisit 0.982 0.976 0.989 0.000

The lowest 10^th^ percentile:
 exp(Est.) 2.5% 97.5% P
(Intercept) 0.542 0.422 0.697 0
AgeVisit 0.986 0.981 0.992 0

## HRV (adjusted for resting heart rate):

### SDNN:


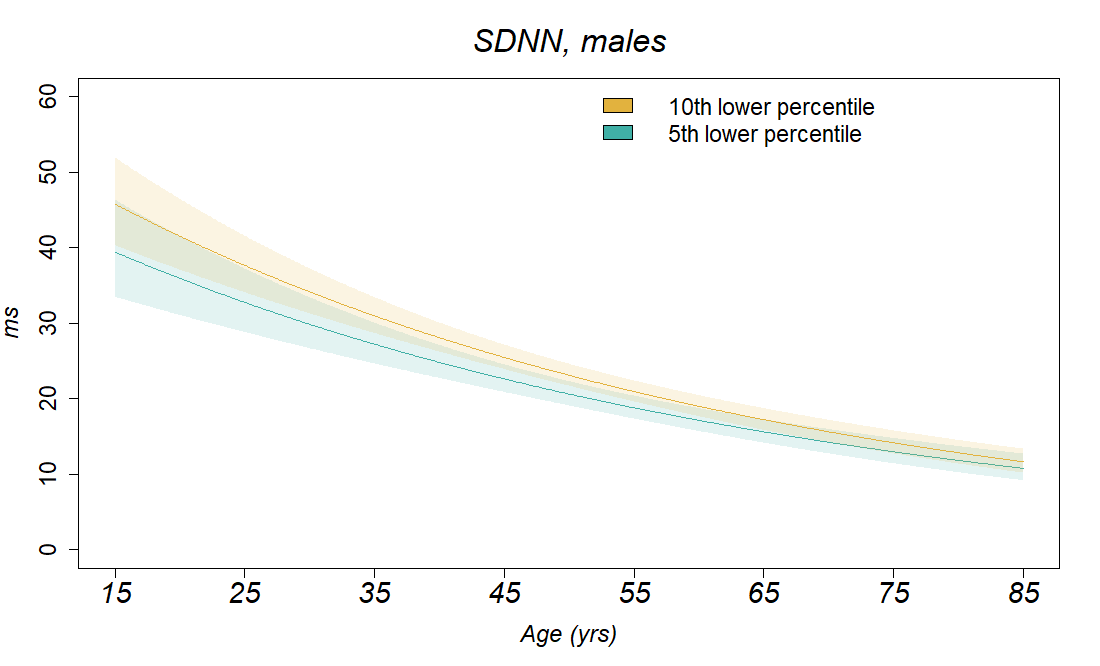


The lowest 5^th^ percentile:

exp(Est.) 2.5% 97.5% P
(Intercept) 183.206 102.217 328.364 0
AgeVisit 0.982 0.978 0.986 0
HR_BPM 0.981 0.974 0.989 0

The lowest 10^th^ percentile:

exp(Est.) 2.5% 97.5% P
(Intercept) 265.398 164.936 427.052 0
AgeVisit 0.981 0.977 0.984 0
HR_BPM 0.978 0.972 0.985 0

### RMSSD:


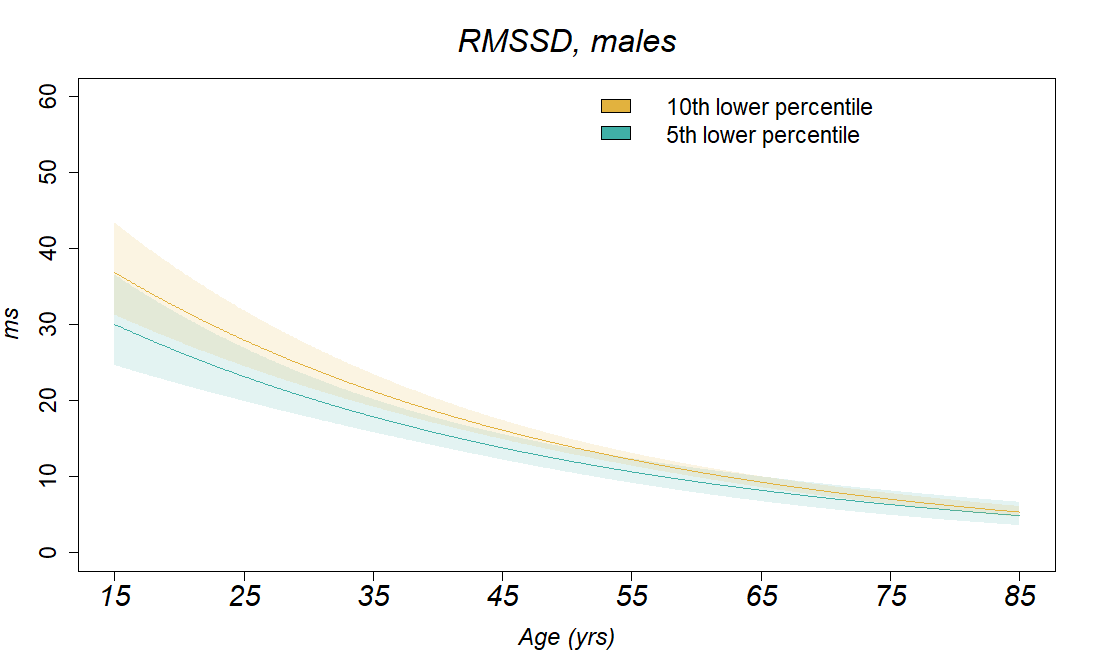


The lowest 5^th^ percentile:

exp(Est.) 2.5% 97.5% P
(Intercept) 428.184 179.990 1018.621 0
AgeVisit 0.974 0.968 0.981 0
HR_BPM 0.967 0.956 0.978 0

The lowest 10^th^ percentile:

exp(Est.) 2.5% 97.5% P
(Intercept) 745.850 442.782 1256.356 0
AgeVisit 0.973 0.969 0.976 0
HR_BPM 0.962 0.956 0.968 0

### HF power:


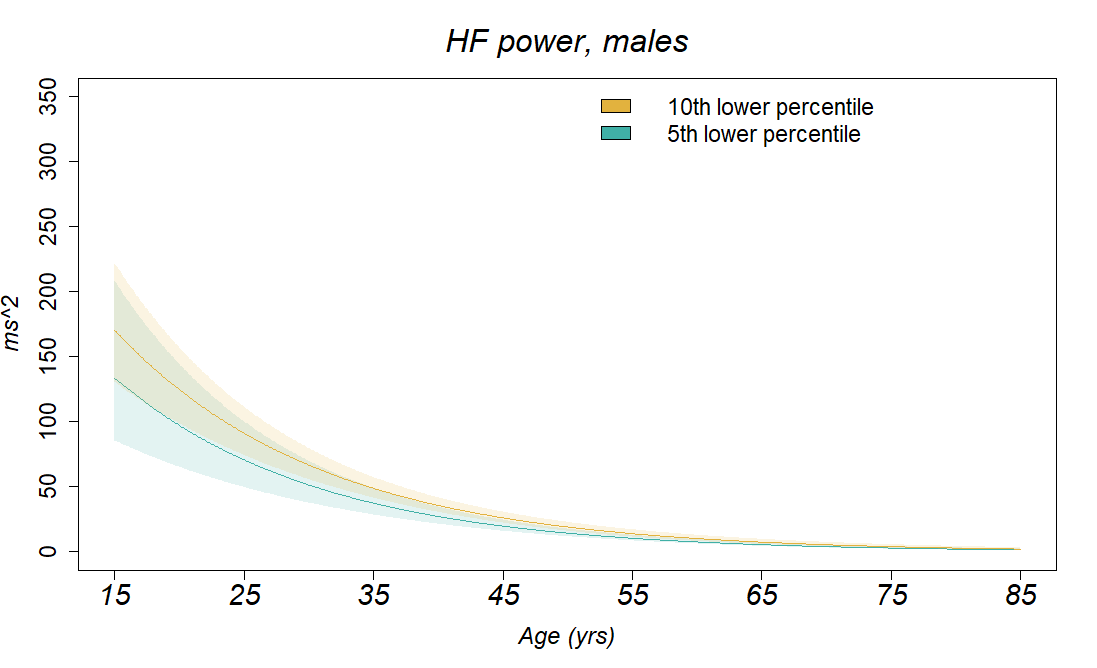


The lowest 5^th^ percentile:

exp(Est.) 2.5% 97.5% P
(Intercept) 29113.538 5986.570 141583.261 0
AgeVisit 0.938 0.928 0.948 0
HR_BPM 0.936 0.916 0.956 0

The lowest 10^th^ percentile:
 exp(Est.) 2.5% 97.5% P
(Intercept) 44701.378 13899.039 143766.278 0
AgeVisit 0.939 0.932 0.947 0
HR_BPM 0.933 0.919 0.948 0

### LF power:


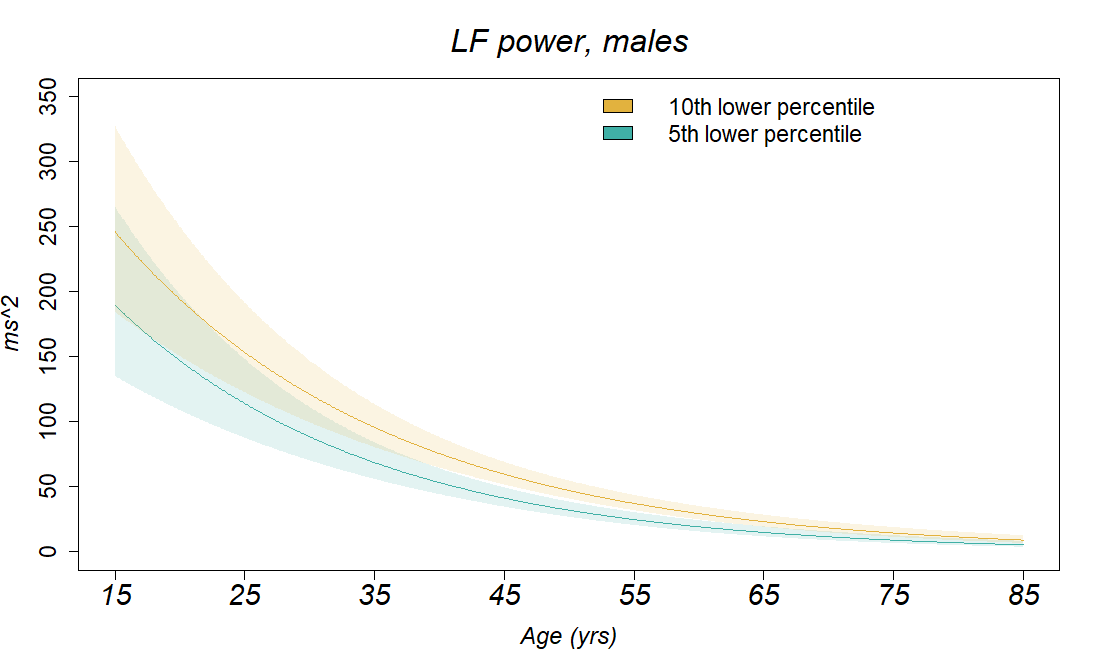


The lowest 5^th^ percentile:

exp(Est.) 2.5% 97.5% P
(Intercept) 1948.403 517.926 7329.760 0.000
AgeVisit 0.950 0.942 0.959 0.000
HR_BPM 0.977 0.960 0.994 0.009

The lowest 10^th^ percentile:
 exp(Est.) 2.5% 97.5% P
(Intercept) 3215.824 1068.782 9675.992 0
AgeVisit 0.954 0.947 0.961 0
HR_BPM 0.973 0.958 0.987 0

### Total power:


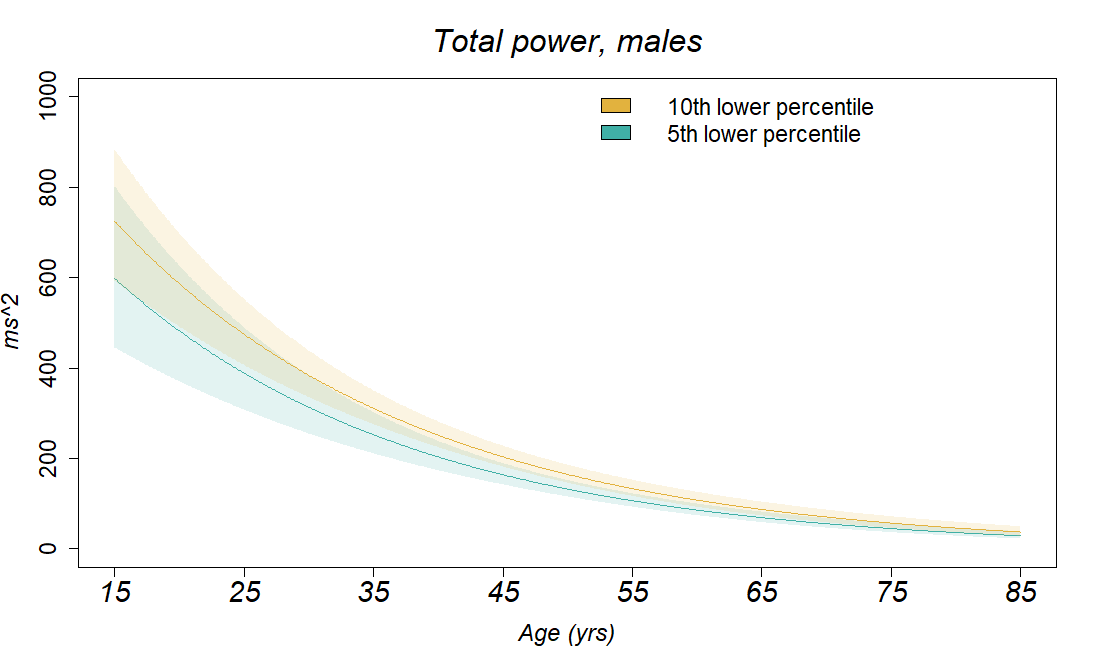


The lowest 5^th^ percentile:

exp(Est.) 2.5% 97.5% P
(Intercept) 11844.434 4125.324 34007.178 0
AgeVisit 0.958 0.951 0.965 0
HR_BPM 0.966 0.952 0.980 0

The lowest 10^th^ percentile:
 exp(Est.) 2.5% 97.5% P
(Intercept) 20612.328 8778.652 48397.875 0
AgeVisit 0.959 0.953 0.964 0
HR_BPM 0.960 0.949 0.972 0

## Frequency domain analyses during the CARTs:

### HF power during Lying-to-standing:


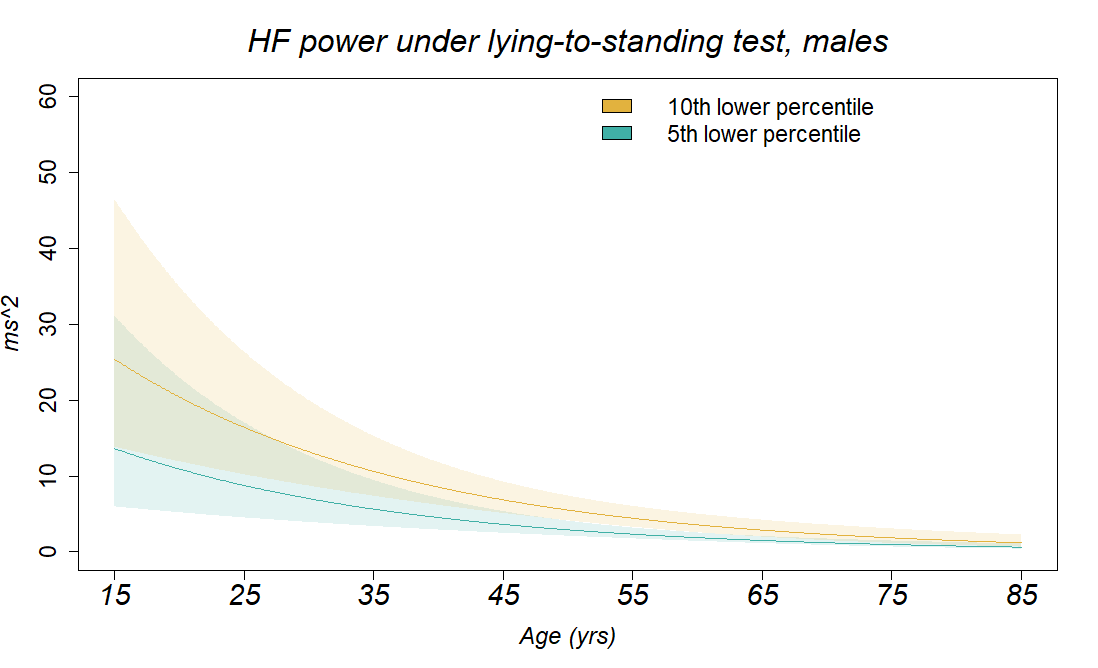


The lowest 5^th^ percentile:

exp(Est.) 2.5% 97.5% P
(Intercept) 441.680 33.019 5908.208 0.000
AgeVisit 0.957 0.940 0.974 0.000
HR_BPM 0.959 0.927 0.992 0.015

The lowest 10^th^ percentile:
 exp(Est.) 2.5% 97.5% P
(Intercept) 2390.564 252.385 22643.135 0
AgeVisit 0.957 0.943 0.972 0
HR_BPM 0.944 0.916 0.972 0

### LF power during Lying-to-standing:


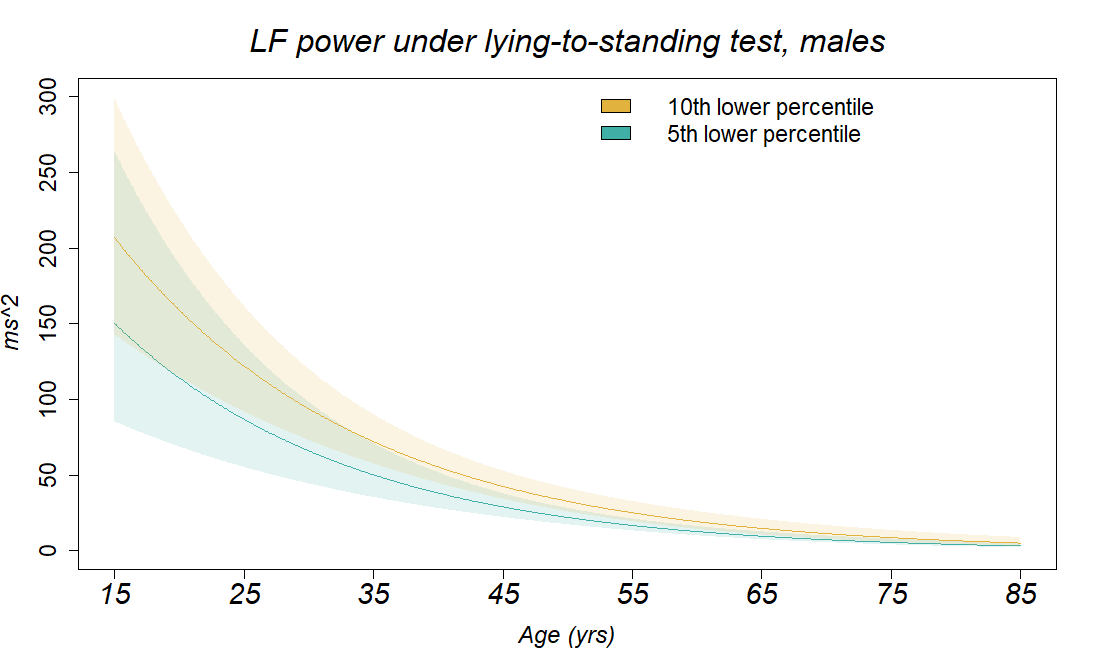


The lowest 5^th^ percentile:

exp(Est.) 2.5% 97.5% P
(Intercept) 11306.197 1689.792 75648.410 0
AgeVisit 0.947 0.935 0.959 0
HR_BPM 0.949 0.925 0.974 0

The lowest 10^th^ percentile:
 exp(Est.) 2.5% 97.5% P
(Intercept) 11527.134 2320.766 57254.714 0
AgeVisit 0.949 0.938 0.960 0
HR_BPM 0.953 0.931 0.975 0

### HF power during Deep-breathing:


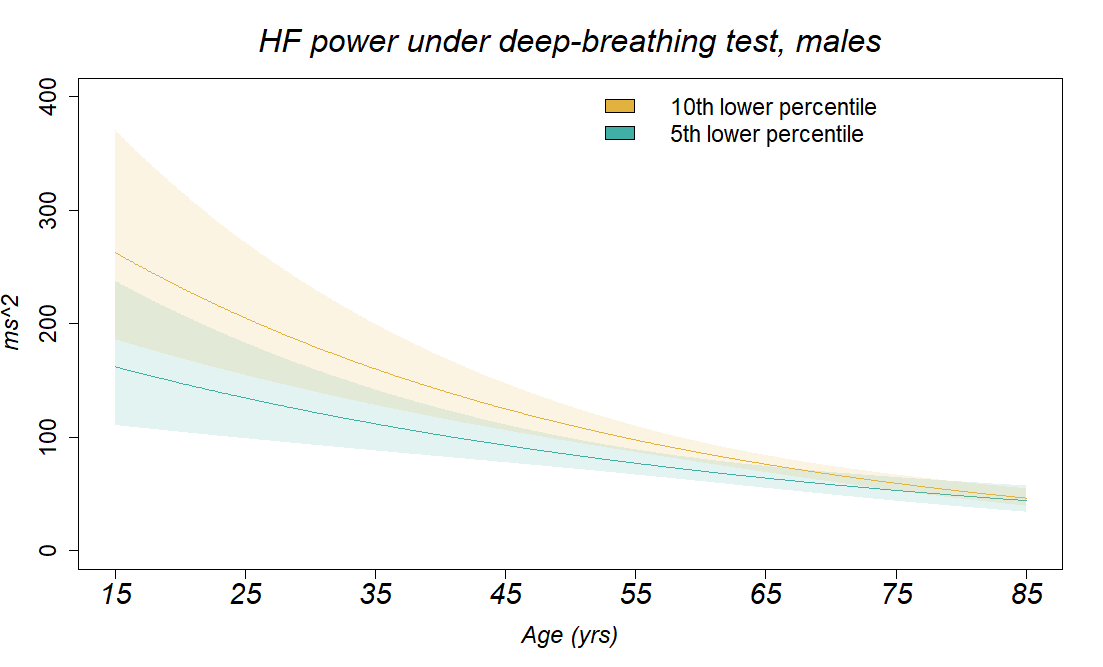


The lowest 5^th^ percentile:

exp(Est.) 2.5% 97.5% P
(Intercept) 983.604 303.437 3188.395 0.000
AgeVisit 0.982 0.974 0.990 0.000
HR_BPM 0.978 0.963 0.993 0.003

The lowest 10^th^ percentile:
 exp(Est.) 2.5% 97.5% P
(Intercept) 2518.390 1062.275 5970.480 0
AgeVisit 0.976 0.969 0.982 0
HR_BPM 0.972 0.962 0.983 0

### LF power during Deep-breathing:


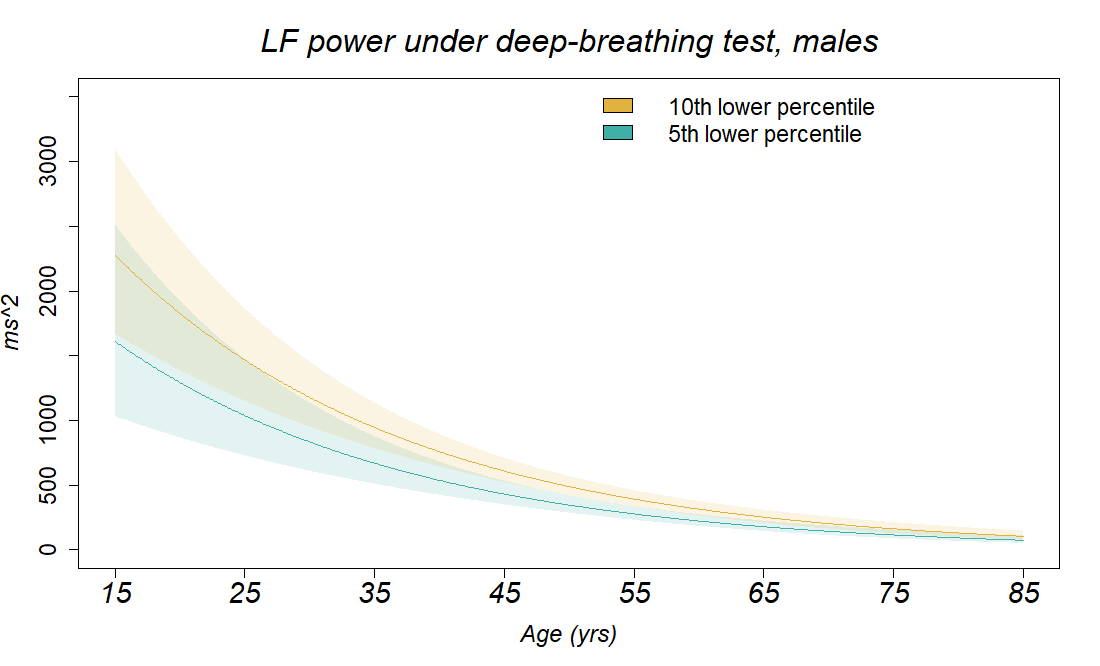


The lowest 5^th^ percentile:

exp(Est.) 2.5% 97.5% P
(Intercept) 25002.219 5503.277 113588.853 0.000
AgeVisit 0.957 0.947 0.967 0.000
HR_BPM 0.969 0.950 0.989 0.003

The lowest 10^th^ percentile:
 exp(Est.) 2.5% 97.5% P
(Intercept) 21542.174 6749.464 68755.869 0.000
AgeVisit 0.957 0.949 0.965 0.000
HR_BPM 0.977 0.962 0.992 0.003

### HF power during Valsalva manoeuvre:


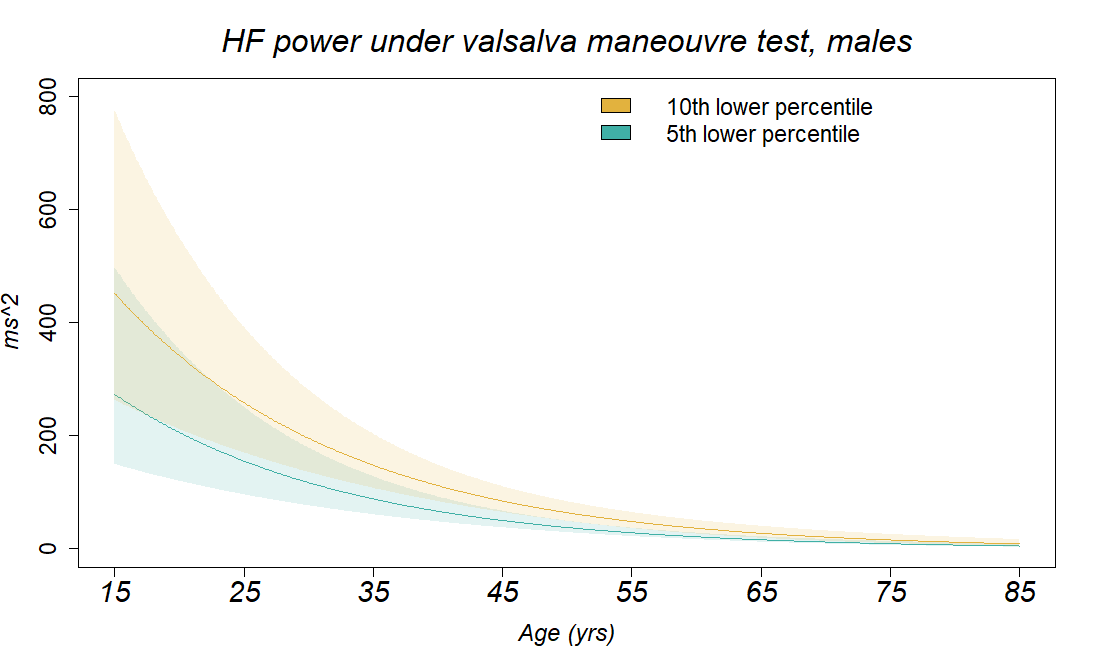


The lowest 5^th^ percentile:

exp(Est.) 2.5% 97.5% P
(Intercept) 8465.541 1464.858 48923.106 0
AgeVisit 0.945 0.932 0.958 0
HR_BPM 0.962 0.942 0.983 0

The lowest 10^th^ percentile:
 exp(Est.) 2.5% 97.5% P
(Intercept) 14113.177 1723.764 115550.50 0.000
AgeVisit 0.946 0.932 0.96 0.000
HR_BPM 0.962 0.935 0.99 0.008

### LF power during Valsalva manoeuvre:


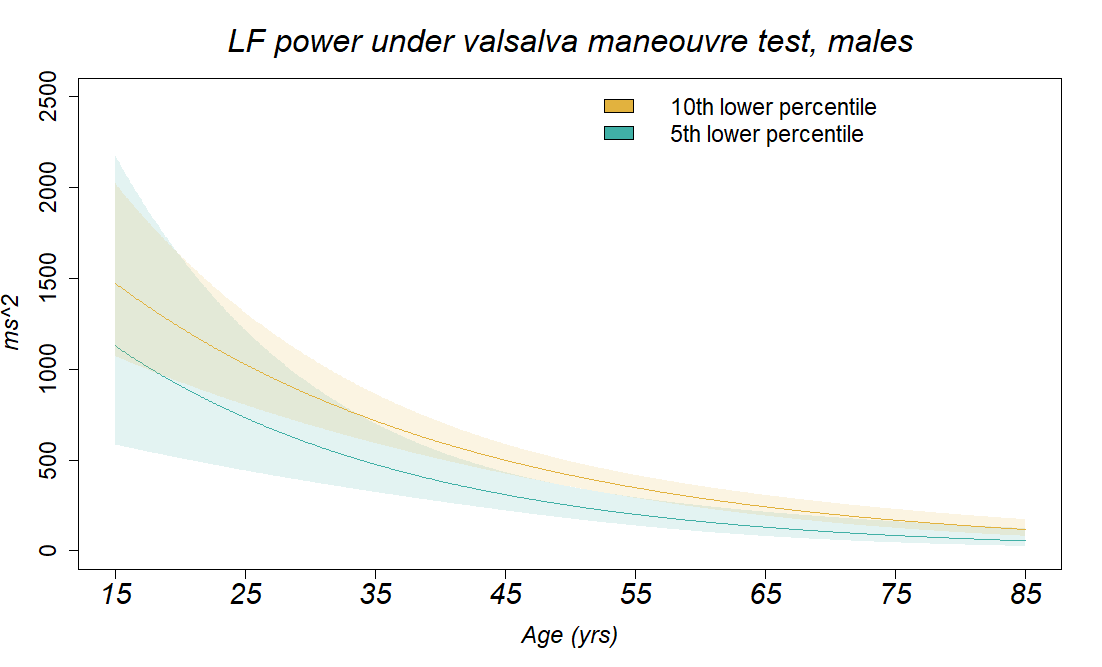


The lowest 5^th^ percentile:

exp(Est.) 2.5% 97.5% P
(Intercept) 14375.765 1022.436 202127.691 0.000
AgeVisit 0.958 0.940 0.976 0.000
HR_BPM 0.972 0.937 1.008 0.126

The lowest 10^th^ percentile:
 exp(Est.) 2.5% 97.5% P
(Intercept) 16734.605 4694.134 59658.929 0.000
AgeVisit 0.965 0.956 0.973 0.000
HR_BPM 0.972 0.955 0.990 0.002

# Formulas

## Total study population

The final formulas are derived from data based on the total study population, which includes both males and females. The formulas are presented for both the lower 5^th^ and 10^th^ percentile.

### CARTs:

$${Lying-to-standing threshold}_{5th percentile}=\exp\left( -1.31-0.029*age \right)+1$$

$${Lying-to-standing threshold}_{10th percentile}=\exp\left( -0.95-0.03*age \right)+1$$

$${Deep-breathing threshold}_{5th percentile}=exp\left( -0.72-0.034*age \right)+1$$

$${Deep-breathing threshold}_{10th percentile}=exp\left( -0.45-0.034*age \right)+1$$

$${Valsalva maneouvre threshold}_{5th percentile}=exp\left( -0.92-0.011*age \right)+1$$

$${Valsalva maneouvre threshold}_{10th percentile}=exp\left( -0.67-0.012*age \right)+1$$

### HRV:

$${SDNN threshold}_{5th percentile}=exp\left( 5.44-0.018*age-0.022*heart rate \right)$$

$${SDNN threshold}_{10th percentile}=exp\left( 5.7-0.019*age-0.024*heart rate \right)$$

$${RMSSD threshold}_{5th percentile}=exp\left( 6.46-0.025*age-0.039*heart rate \right)$$

$${RMSSD threshold}_{10th percentile}=exp\left( 6.30-0.025*age-0.034*heart rate \right)$$

$${HF power threshold}_{5th percentile}=exp\left( 10.21-0.058*age-0.066*heart rate \right)$$

$${HF power threshold}_{10th percentile}=exp\left( 10.31-0.053*age-0.065*heart rate \right)$$

$${LF power threshold}_{5th percentile}=exp\left( 7.86-0.047*age-0.031*heart rate \right)$$

$${LF power threshold}_{10th percentile}=exp\left( 8.11-0.046*age-0.032*heart rate \right)$$

$${Total power threshold}_{5th percentile}=exp\left( 9.33-0.039*age-0.037*heart rate \right)$$

$${Total power threshold}_{10th percentile}=exp\left( 9.72-0.037*age-0.040*heart rate \right)$$

### Frequency domain analyses for the CARTs:

$${HF power threshold (RS)}_{5th percentile}=exp\left( 6.53-0.036*age-0.05*heart rate \right)$$

$${HF power threshold (RS)}_{10th percentile}=exp\left( 6.57-0.037*age-0.042*heart rate \right)$$

$${LF power threshold (RS)}_{5th percentile}=exp\left( 8.96-0.051*age-0.051*heart rate \right)$$

$${LF power threshold (RS)}_{10th percentile}=exp\left( 9.21-0.049*age-0.05*heart rate \right)$$

$${HF power threshold (E:I)}_{5th percentile}=exp\left( 7.57-0.023*age-0.028*heart rate \right)$$

$${HF power threshold (E:I)}_{10th percentile}=exp\left( 7.99-0.027*age-0.028*heart rate \right)$$

$${LF power threshold (E:I)}_{5th percentile}=exp\left( 9.94-0.045*age-0.025*heart rate \right)$$

$${LF power threshold (E:I)}_{10th percentile}=exp\left( 10.38-0.042*age-0.029*heart rate \right)$$

$${HF power threshold (VM)}_{5th percentile}=exp\left( 8.37-0.05*age-0.031*heart rate \right)$$

$${HF power threshold (VM)}_{10th percentile}=exp\left( 9.53-0.055*age-0.037*heart rate \right)$$

$${LF power threshold (VM)}_{5th percentile}=exp\left( 8.8-0.034*age-0.024*heart rate \right)$$

$${LF power threshold (VM)}_{10th percentile}=exp\left( 9.5-0.03*age-0.03*heart rate \right)$$

## Sex stratified study population

The formulas presented below are derived from data obtained from a sex-stratified study population. This means that separate formulas are provided for females and males, along with the corresponding lower 5th and 10th percentiles.

### Females:

### CARTs:

$${Lying-to-standing threshold}_{Females, 5th percentile}=\exp\left( -1.24-0.031*age \right)+1$$

$${Lying-to-standing threshold}_{Females, 10th percentile}=\exp\left( -0.95-0.03*age \right)+1$$

$${Deep-breathing threshold}_{Females, 5th percentile}=exp\left( -0.73-0.029*age \right)+1$$

$${Deep-breathing threshold}_{Females, 10th percentile}=exp\left( -0.55-0.029*age \right)+1$$

$${Valsalva maneouvre threshold}_{Females, 5th percentile}=exp\left( -1.35-0.002*age \right)+1$$

$${Valsalva maneouvre threshold}_{Females, 10th percentile}=exp\left( -0.98-0.005*age \right)+1$$

### HRV:

$${SDNN threshold}_{Females,5th percentile}=exp\left( 5.65-0.017*age-0.026*heart rate \right)$$

$${SDNN threshold}_{Females, 10th percentile}=exp\left( 5.76-0.017*age-0.025*heart rate \right)$$

$${RMSSD threshold}_{Females, 5th percentile}=exp\left( 6.16-0.023*age-0.034*heart rate \right)$$

$${RMSSD threshold}_{Females, 10th percentile}=exp\left( 6.28-0.023*age-0.035*heart rate \right)$$

$${HF power threshold}_{Females, 5th percentile}=exp\left( 10.34-0.044*age-0.072*heart rate \right)$$

$${HF power threshold}_{Females,10th percentile}=exp\left( 10.15-0.043*age-0.066*heart rate \right)$$

$${LF power threshold}_{Females, 5th percentile}=exp\left( 8.53-0.048*age-0.041*heart rate \right)$$

$${LF power threshold}_{Females, 10th percentile}=exp\left( 8.22-0.046*age-0.034*heart rate \right)$$

$${Total power threshold}_{Females, 5th percentile}=exp\left( 9.3-0.034*age-0.039*heart rate \right)$$

$${Total power threshold}_{Females, 10th percentile}=exp\left( 9.82-0.035*age-0.042*heart rate \right)$$

### Frequency domain analyses for the CARTs:

$${HF power threshold (RS)}_{Females, 5th percentile}=exp\left( 5.41-0.027*age-0.035*heart rate \right)$$

$${HF power threshold (RS)}_{Females,10th percentile}=exp\left( 5.7-0.032*age-0.032*heart rate \right)$$

$${LF power threshold (RS)}_{Females, 5th percentile}=exp\left( 9.18-0.046*age-0.057*heart rate \right)$$

$${LF power threshold (RS)}_{Females,10th percentile}=exp\left( 9.31-0.042*age-0.057*heart rate \right)$$

$${HF power threshold (E:I)}_{Females, 5th percentile}=exp\left( 7.73-0.024*age-0.029*heart rate \right)$$

$${HF power threshold (E:I)}_{Females, 10th percentile}=exp\left( 7.86-0.027*age-0.026*heart rate \right)$$

$${LF power threshold (E:I)}_{Females, 5th percentile}=exp\left( 10.93-0.045*age-0.037*heart rate \right)$$

$${LF power threshold (E:I)}_{Females, 10th percentile}=exp\left( 10.98-0.044*age-0.033*heart rate \right)$$

$${HF power threshold (VM)}_{Females, 5th percentile}=exp\left( 8.56-0.035*age-0.041*heart rate \right)$$

$${HF power threshold (VM)}_{Females,10th percentile}=exp\left( 9.02-0.046*age-0.031*heart rate \right)$$

$${LF power threshold (VM)}_{Females, 5th percentile}=exp\left( 9.34-0.039*age-0.032*heart rate \right)$$

$${LF power threshold (VM)}_{Females, 10th percentile}=exp\left( 8.59-0.025*age-0.022*heart rate \right)$$

## Males

### CARTs:

$${Lying-to-standing threshold}_{Males, 5th percentile}=\exp\left( -1.38-0.027*age \right)+1$$

$${Lying-to-standing threshold}_{Males, 10th percentile}=\exp\left( -1.02-0.029*age \right)+1$$

$${Deep-breathing threshold}_{Males, 5th percentile}=exp\left( -0.79-0.036*age \right)+1$$

$${Deep-breathing threshold}_{Males, 10th percentile}=exp\left( -0.45-0.037*age \right)+1$$

$${Valsalva maneouvre threshold}_{Males, 5th percentile}=exp\left( -0.62-0.018*age \right)+1$$

$${Valsalva maneouvre threshold}_{Males, 10th percentile}=exp\left( -0.61-0.014*age \right)+1$$

### HRV:

$${SDNN threshold}_{Males,5th percentile}=exp\left( 5.21-0.018*age-0.019*heart rate \right)$$

$${SDNN threshold}_{Males, 10th percentile}=exp\left( 5.58-0.02*age-0.022*heart rate \right)$$

$${RMSSD threshold}_{Males, 5th percentile}=exp\left( 6.06-0.026*age-0.034*heart rate \right)$$

$${RMSSD threshold}_{Males, 10th percentile}=exp\left( 6.62-0.028*age-0.039*heart rate \right)$$

$${HF power threshold}_{Males, 5th percentile}=exp\left( 10.28-0.064*age-0.066*heart rate \right)$$

$${HF power threshold}_{Males,10th percentile}=exp\left( 10.71-0.062*age-0.069*heart rate \right)$$

$${LF power threshold}_{Males, 5th percentile}=exp\left( 7.58-0.051*age-0.023*heart rate \right)$$

$${LF power threshold}_{Males, 10th percentile}=exp\left( 8.08-0.047*age-0.028*heart rate \right)$$

$${Total power threshold}_{Males, 5th percentile}=exp\left( 9.38-0.043*age-0.035*heart rate \right)$$

$${Total power threshold}_{Males, 10th percentile}=exp\left( 9.93-0.042*age-0.041*heart rate \right)$$

### Frequency domain analyses for the CARTs:

$${HF power threshold (RS)}_{Males, 5th percentile}=exp\left( 6.09-0.044*age-0.042*heart rate \right)$$

$${HF power threshold (RS)}_{Males,10th percentile}=exp\left( 7.78-0.044*age-0.058*heart rate \right)$$

$${LF power threshold (RS)}_{Males, 5th percentile}=exp\left( 9.33-0.055*age-0.052*heart rate \right)$$

$${LF power threshold (RS)}_{Males,10th percentile}=exp\left( 9.35-0.053*age-0.048*heart rate \right)$$

$${HF power threshold (E:I)}_{Males, 5th percentile}=exp\left( 6.89-0.019*age-0.023*heart rate \right)$$

$${HF power threshold (E:I)}_{Males, 10th percentile}=exp\left( 7.83-0.025*age-0.028*heart rate \right)$$

$${LF power threshold (E:I)}_{Males, 5th percentile}=exp\left( 10.13-0.044*age-0.031*heart rate \right)$$

$${LF power threshold (E:I)}_{Males, 10th percentile}=exp\left( 9.98-0.044*age-0.024*heart rate \right)$$

$${HF power threshold (VM)}_{Males, 5th percentile}=exp\left( 9.04-0.056*age-0.039*heart rate \right)$$

$${HF power threshold (VM)}_{Males, 10th percentile}=exp\left( 9.56-0.056*age-0.039*heart rate \right)$$

$${LF power threshold (VM)}_{Males,5th percentile}=exp\left( 9.57-0.043*age-0.028*heart rate \right)$$

$${LF power threshold (VM)}_{Males, 10th percentile}=exp\left( 9.73-0.036*age-0.028*heart rate \right)$$

# Tables

## CARTs

Tables with normative thresholds estimated at the lowest 5^th^ percentile, divided into five-year age groups (15-19, 20-24, 25-29, etc.)

| Appendix table 2: Age-specific normative thresholds for the CARTs estimated at the lower 5^th^ percentile. | | | | | | | | |
| --- | --- | --- | --- | --- | --- | --- | --- | --- |
|  |  | | **Lying-to-standing ratio** | |  | **Deep-breathing ratio** |  | **Valsalva manoeuvre ratio** |
|  |  | | N=739 | |  | N=845 |  | N=651 |
| Age (yrs) | | N | | Estimate (95% CI) | N | Estimate (95% CI) | N | Estimate (95% CI) |
| *15-19* | | 68 | | 1.16 (1.12;1.21) | 78 | 1.27 (1.22;1.33) | 59 | 1.33 (1.26;1.41) |
| *20-24* | | 47 | | 1.14 (1.11;1.18) | 50 | 1.23 (1.19;1.27) | 36 | 1.31 (1.25;1.38) |
| *25-29* | | 51 | | 1.12 (1.10;1.15) | 51 | 1.19 (1.16;1.23) | 45 | 1.29 (1.25;1.35) |
| *30-34* | | 64 | | 1.11 (1.09;1.13) | 72 | 1.16 (1.14;1.19) | 58 | 1.28 (1.24;1.32) |
| *35-39* | | 47 | | 1.09 (1.08;1.11) | 55 | 1.14 (1.12;1.15) | 51 | 1.26 (1.23;1.30) |
| *40-44* | | 58 | | 1.08 (1.07;1.09) | 66 | 1.11 (1.10;1.13) | 56 | 1.25 (1.22;1.28) |
| *45-49* | | 48 | | 1.07 (1.06;1.08) | 53 | 1.10 (1.09;1.11) | 46 | 1.23 (1.21;1.26) |
| *50-54* | | 62 | | 1.06 (1.05;1.07) | 78 | 1.08 (1.07;1.09) | 71 | 1.22 (1.20;1.24) |
| *55-59* | | 62 | | 1.05 (1.04;1.06) | 75 | 1.07 (1.06;1.08) | 56 | 1.21 (1.19;1.23) |
| *60-64* | | 59 | | 1.04 (1.04;1.05) | 66 | 1.06 (1.05;1.07) | 50 | 1.20 (1.18;1.22) |
| *65-69* | | 60 | | 1.04 (1.03;1.05) | 69 | 1.05 (1.04;1.06) | 49 | 1.19 (1.17;1.21) |
| *70-74* | | 38 | | 1.03 (1.03;1.04) | 46 | 1.04 (1.03;1.05) | 32 | 1.18 (1.16;1.20) |
| *75-79* | | 56 | | 1.03 (1.02;1.04) | 61 | 1.03 (1.03;1.04) | 31 | 1.17 (1.15;1.19) |
| *80-85* | | 19 | | 1.02 (1.02;1.03) | 25 | 1.03 (1.02;1.04) | 11 | 1.16 (1.14;1.19) |
| *Results from log-transformed models. Estimates are back-transformed. The results are estimated from the median age of the specific age spans and are presented as ratios with 95% CI.*  *CARTs: Cardiovascular autonomic reflex tests* SDNN | | | | | | | | |

| Appendix table 3: Age- and heart-rate-specific normative thresholds estimated at the lower 5^th^ percentile. | | | | | | |
| --- | --- | --- | --- | --- | --- | --- |
|  | **SDNN (ms),** Estimate (95% CI)  N=839 | | | | | |
| Age (yrs) | Heart rate (bpm) | | | | | |
|  | *40-50* | *50-60* | *60-70* | *70-80* | *80-90* | *90-100* |
| *15-19* | 62.19 (53.23;72.65) | 49.94 (44.17;56.46) | 40.11 (36.35;44.25) | 32.21 (29.46;35.21) | 25.86 (23.41;28.58) | 20.77 (18.33;23.54) |
| *20-24* | 56.8 (48.91;65.97) | 45.62 (40.65;51.19) | 36.63 (33.52;40.03) | 29.42 (27.19;31.82) | 23.62 (21.58;25.86) | 18.97 (16.86;21.35) |
| *25-29* | 51.88 (44.89;59.96) | 41.66 (37.37;46.45) | 33.46 (30.88;36.25) | 26.87 (25.08;28.79) | 21.58 (19.88;23.43) | 17.33 (15.5;19.38) |
| *30-34* | 47.39 (41.17;54.55) | 38.06 (34.31;42.21) | 30.56 (28.41;32.87) | 24.54 (23.11;26.07) | 19.71 (18.28;21.25) | 15.83 (14.23;17.61) |
| *35-39* | 43.28 (37.71;49.69) | 34.76 (31.46;38.4) | 27.91 (26.1;29.86) | 22.42 (21.25;23.64) | 18 (16.79;19.3) | 14.46 (13.04;16.02) |
| *40-44* | 39.54 (34.5;45.3) | 31.75 (28.81;34.99) | 25.5 (23.92;27.18) | 20.48 (19.5;21.5) | 16.44 (15.39;17.57) | 13.2 (11.94;14.6) |
| *45-49* | 36.11 (31.53;41.36) | 29 (26.33;31.94) | 23.29 (21.87;24.8) | 18.7 (17.83;19.61) | 15.02 (14.07;16.03) | 12.06 (10.91;13.33) |
| *50-54* | 32.98 (28.78;37.8) | 26.49 (24.03;29.2) | 21.27 (19.95;22.68) | 17.08 (16.26;17.95) | 13.72 (12.83;14.67) | 11.02 (9.96;12.19) |
| *55-59* | 30.13 (26.24;34.59) | 24.19 (21.89;26.74) | 19.43 (18.15;20.8) | 15.6 (14.77;16.48) | 12.53 (11.67;13.45) | 10.06 (9.07;11.16) |
| *60-64* | 27.52 (23.89;31.69) | 22.1 (19.91;24.53) | 17.75 (16.48;19.11) | 14.25 (13.4;15.16) | 11.44 (10.6;12.36) | 9.19 (8.25;10.24) |
| *65-69* | 25.13 (21.73;29.07) | 20.18 (18.09;22.53) | 16.21 (14.94;17.59) | 13.02 (12.13;13.97) | 10.45 (9.61;11.37) | 8.39 (7.50;9.40) |
| *70-74* | 22.96 (19.75;26.68) | 18.44 (16.41;20.71) | 14.81 (13.53;16.21) | 11.89 (10.97;12.89) | 9.55 (8.7;10.47) | 7.67 (6.8;8.64) |
| *75-79* | 20.97 (17.93;24.52) | 16.84 (14.88;19.06) | 13.52 (12.24;14.95) | 10.86 (9.91;11.9) | 8.72 (7.88;9.66) | 7.0 (6.17;7.95) |
| *80-85* | 19.15 (16.27;22.54) | 15.38 (13.47;17.56) | 12.35 (11.06;13.79) | 9.92 (8.95;10.99) | 7.97 (7.12;8.91) | 6.4 (5.59;7.32) |
| *Results from log-transformed models. Estimates are back-transformed. The results are estimated from the median age of the specific age spans and are presented as estimates with 95% CI.*  *SDNN: Standard deviation of normal-to-normal intervals* | | | | | | |

## RMSSD:

| Appendix table 4: Age- and heart-rate-specific normative thresholds estimated at the lower 5^th^ percentile. | | | | | | |
| --- | --- | --- | --- | --- | --- | --- |
|  | **RMSSD (ms),** estimate (95% CI) | | | | | |
|  | N=839 | | | | | |
| Age (yrs) | Heart rate (bpm) |  |  |  |  |  |
|  | *40-50* | *50-60* | *60-70* | *70-80* | *80-90* | *90-100* |
| *15-19* | 72.35 (57.02;91.8) | 49.22 (41.1;58.95) | 33.49 (28.81;38.92) | 22.78 (19.33;26.85) | 15.5 (12.51;19.2) | 10.55 (7.96;13.96) |
| *20-24* | 63.7 (50.77;79.93) | 43.34 (36.72;51.16) | 29.49 (25.8;33.69) | 20.06 (17.27;23.3) | 13.65 (11.14;16.72) | 9.28 (7.07;12.2) |
| *25-29* | 56.09 (45.14;69.7) | 38.16 (32.75;44.46) | 25.96 (23.08;29.2) | 17.66 (15.41;20.24) | 12.02 (9.9;14.59) | 8.18 (6.26;10.67) |
| *30-34* | 49.39 (40.07;60.86) | 33.6 (29.17;38.71) | 22.86 (20.62;25.35) | 15.55 (13.73;17.62) | 10.58 (8.78;12.75) | 7.20 (5.54;9.35) |
| *35-39* | 43.49 (35.52;53.24) | 29.58 (25.92;33.77) | 20.13 (18.37;22.05) | 13.69 (12.19;15.38) | 9.32 (7.77;11.17) | 6.34 (4.90;8.21) |
| *40-44* | 38.29 (31.42;46.67) | 26.05 (22.97;29.55) | 17.72 (16.31;19.25) | 12.06 (10.8;13.46) | 8.20 (6.86;9.80) | 5.58 (4.32;7.21) |
| *45-49* | 33.71 (27.73;40.99) | 22.94 (20.29;25.93) | 15.6 (14.42;16.89) | 10.62 (9.53;11.83) | 7.22 (6.05;8.63) | 4.91 (3.80;6.35) |
| *50-54* | 29.68 (24.43;36.07) | 20.2 (17.86;22.83) | 13.74 (12.68;14.89) | 9.35 (8.37;10.43) | 6.36 (5.32;7.61) | 4.33 (3.34;5.60) |
| *55-59* | 26.14 (21.47;31.82) | 17.78 (15.68;20.17) | 12.1 (11.1;13.19) | 8.23 (7.33;9.24) | 5.60 (4.66;6.72) | 3.81 (2.94;4.94) |
| *60-64* | 23.01 (18.83;28.12) | 15.66 (13.71;17.88) | 10.65 (9.67;11.73) | 7.25 (6.40;8.20) | 4.93 (4.08;5.96) | 3.35 (2.57;4.37) |
| *65-69* | 20.26 (16.49;24.9) | 13.79 (11.96;15.89) | 9.38 (8.41;10.47) | 6.38 (5.58;7.30) | 4.34 (3.57;5.29) | 2.95 (2.25;3.87) |
| *70-74* | 17.84 (14.41;22.09) | 12.14 (10.41;14.15) | 8.26 (7.29;9.36) | 5.62 (4.84;6.52) | 3.82 (3.11;4.70) | 2.60 (1.97;3.44) |
| *75-79* | 15.71 (12.57;19.63) | 10.69 (9.05;12.62) | 7.27 (6.31;8.37) | 4.95 (4.20;5.82) | 3.37 (2.71;4.18) | 2.29 (1.72;3.05) |
| *80-85* | 13.83 (10.95;17.47) | 9.41 (7.85;11.28) | 6.40 (5.46;7.50) | 4.36 (3.64;5.21) | 2.96 (2.35;3.73) | 2.02 (1.50;2.71) |
| *Results from log-transformed models. Estimates are back-transformed. The results are estimated from the median age of the specific age spans and are presented as estimates with 95% CI.*  *RMSSD:*  *Root mean square of the sum of the squares of differences between consecutive R–R intervals* | | | | | | |

## HF power:

| Appendix table 5: Age- and heart-rate-specific normative thresholds estimated at the lower 5^th^ percentile. | | | | | | |
| --- | --- | --- | --- | --- | --- | --- |
|  | **HF Power (ms²),** estimate (95% CI) | | | | | |
|  | N=839 | | | | | |
| Age (yrs) | Heart rate (bpm) |  |  |  |  |  |
|  | 40-50 | 50-60 | 60-70 | 70-80 | 80-90 | 90-100 |
| 15-19 | 493.76 (302.08;807.05) | 254.29 (177.13;365.07) | 130.97 (99.62;172.18) | 67.45 (51.41;88.49) | 34.74 (24.31;49.63) | 17.89 (11.01;29.07) |
| 20-24 | 369.03 (229.98;592.14) | 190.05 (135.64;266.31) | 97.88 (76.81;124.73) | 50.41 (39.59;64.2) | 25.96 (18.55;36.33) | 13.37 (8.35;21.42) |
| 25-29 | 275.81 (174.51;435.89) | 142.04 (103.45;195.04) | 73.16 (58.99;90.72) | 37.68 (30.34;46.78) | 19.4 (14.1;26.71) | 9.99 (6.3;15.84) |
| 30-34 | 206.13 (131.94;322.04) | 106.16 (78.52;143.53) | 54.68 (45.04;66.37) | 28.16 (23.11;34.3) | 14.5 (10.65;19.74) | 7.47 (4.74;11.76) |
| 35-39 | 154.06 (99.36;238.87) | 79.34 (59.27;106.21) | 40.86 (34.12;48.94) | 21.05 (17.46;25.36) | 10.84 (8.01;14.67) | 5.58 (3.56;8.76) |
| 40-44 | 115.14 (74.52;177.9) | 59.3 (44.47;79.08) | 30.54 (25.6;36.44) | 15.73 (13.07;18.93) | 8.1 (5.98;10.97) | 4.17 (2.65;6.56) |
| 45-49 | 86.06 (55.66;133.06) | 44.32 (33.15;59.26) | 22.83 (19.01;27.41) | 11.76 (9.69;14.27) | 6.05 (4.44;8.26) | 3.12 (1.97;4.93) |
| 50-54 | 64.32 (41.39;99.94) | 33.12 (24.56;44.68) | 17.06 (13.98;20.81) | 8.79 (7.12;10.85) | 4.52 (3.28;6.25) | 2.33 (1.46;3.72) |
| 55-59 | 48.07 (30.66;75.37) | 24.76 (18.09;33.88) | 12.75 (10.21;15.92) | 6.57 (5.19;8.30) | 3.38 (2.41;4.75) | 1.74 (1.08;2.81) |
| 60-64 | 35.93 (22.62;57.06) | 18.5 (13.26;25.82) | 9.53 (7.42;12.24) | 4.91 (3.77;6.38) | 2.53 (1.76;3.63) | 1.3 (0.79;2.14) |
| 65-69 | 26.85 (16.63;43.35) | 13.83 (9.68;19.76) | 7.12 (5.37;9.45) | 3.67 (2.73;4.93) | 1.89 (1.28;2.78) | 0.97 (0.58;1.63) |
| 70-74 | 20.07 (12.19;33.04) | 10.34 (7.04;15.17) | 5.32 (3.88;7.31) | 2.74 (1.97;3.81) | 1.41 (0.93;2.14) | 0.73 (0.42;1.25) |
| 75-79 | 15.0 (8.91;25.25) | 7.72 (5.11;11.68) | 3.98 (2.79;5.67) | 2.05 (1.42;2.96) | 1.06 (0.68;1.65) | 0.54 (0.31;0.95) |
| 80-85 | 11.21 (6.50;19.35) | 5.77 (3.70;9.01) | 2.97 (2.01;4.40) | 1.53 (1.02;2.29) | 0.79 (0.49;1.27) | 0.41 (0.23;0.73) |
| *Results from log-transformed models. Estimates are back-transformed. The results are estimated from the median age of the specific age spans and are presented as estimates with 95% CI.*  *HF power: High-frequency power* | | | | | | |

## LF power:

| Appendix table 6: Age- and heart-rate-specific normative thresholds estimated at the lower 5^th^ percentile. | | | | | | |
| --- | --- | --- | --- | --- | --- | --- |
|  | **LF Power (ms²),** Estimate (95% CI) | | | | | |
|  | N= 839 | | | | | |
| Age (yrs) | Heart rate (bpm) |  |  |  |  |  |
|  | *40-50* | *50-60* | *60-70* | *70-80* | *80-90* | *90-100* |
| *15-19* | 281,82 (194,21;408,96) | 206,96 (157,17;272,53) | 151,99 (122,4;188,72) | 111,61 (88,86;140,2) | 81,97 (60,61;110,85) | 60,19 (40,14;90,27) |
| *20-24* | 222,63 (155,77;318,18) | 163,49 (126,68;211) | 120,06 (99,15;145,39) | 88,17 (71,8;108,28) | 64,75 (48,65;86,18) | 47,55 (32,06;70,52) |
| *25-29* | 175,87 (124,64;248,15) | 129,15 (101,83;163,81) | 94,85 (80,11;112,29) | 69,65 (57,84;83,87) | 51,15 (38,93;67,2) | 37,56 (25,55;55,23) |
| *30-34* | 138,93 (99,47;194,05) | 102,02 (81,57;127,61) | 74,92 (64,49;87,05) | 55,02 (46,42;65,22) | 40,41 (31,05;52,58) | 29,67 (20,31;43,36) |
| *35-39* | 109,75 (79,14;152,19) | 80,6 (65,08;99,81) | 59,19 (51,64;67,83) | 43,47 (37,05;50,99) | 31,92 (24,67;41,3) | 23,44 (16,1;34,13) |
| *40-44* | 86,7 (62,78;119,73) | 63,67 (51,68;78,43) | 46,76 (41,07;53,23) | 34,34 (29,4;40,11) | 25,22 (19,52;32,57) | 18,52 (12,72;26,95) |
| *45-49* | 68,49 (49,63;94,5) | 50,29 (40,84;61,94) | 36,94 (32,4;42,1) | 27,12 (23,17;31,76) | 19,92 (15,39;25,79) | 14,63 (10,03;21,33) |
| *50-54* | 54,1 (39,11;74,83) | 39,73 (32,11;49,17) | 29,18 (25,37;33,56) | 21,43 (18,14;25,31) | 15,74 (12,08;20,5) | 11,56 (7,88;16,94) |
| *55-59* | 42,74 (30,73;59,45) | 31,39 (25,12;39,21) | 23,05 (19,73;26,92) | 16,93 (14,12;20,28) | 12,43 (9,45;16,36) | 9,13 (6,18;13,48) |
| *60-64* | 33,76 (24,06;47,37) | 24,79 (19,58;31,4) | 18,21 (15,28;21,7) | 13,37 (10,95;16,33) | 9,82 (7,36;13,1) | 7,21 (4,83;10,76) |
| *65-69* | 26,67 (18,79;37,85) | 19,59 (15,21;25,23) | 14,38 (11,79;17,55) | 10,56 (8,46;13,18) | 7,76 (5,72;10,52) | 5,70 (3,77;8,60) |
| *70-74* | 21,07 (14,64;30,32) | 15,47 (11,78;20,33) | 11,36 (9,08;14,22) | 8,34 (6,53;10,67) | 6,13 (4,44;8,46) | 4,50 (2,94;6,90) |
| *75-79* | 16,64 (11,38;24,35) | 12,22 (9,1;16,42) | 8,98 (6,98;11,55) | 6,59 (5,02;8,65) | 4,84 (3,43;6,83) | 3,55 (2,28;5,54) |
| *80-85* | 13,15 (8,82;19,59) | 9,66 (7,02;13,28) | 7,09 (5,36;9,38) | 5,21 (3,86;7,02) | 3,82 (2,65;5,52) | 2,81 (1,77;4,46) |
| *Results from log-transformed models. Estimates are back-transformed. The results are estimated from the median age of the specific age spans and are presented as estimates with 95% CI.*  *LF power: Low-frequency power* | | | | | | |

## Total power:

| Appendix table 7: Age- and heart-rate-specific normative thresholds estimated at the lower 5^th^ percentile. | | | | | | |
| --- | --- | --- | --- | --- | --- | --- |
|  | **Total power (ms²),** estimate (95% CI) | | | | | |
|  | N=839 | | | | | |
| Age (yrs) | Heart rate (bpm) | | | | | |
|  | 40-50 | 50-60 | 60-70 | 70-80 | 80-90 | 90-100 |
| 15-19 | 1083.57 (775.65;1513.73) | 750.37 (583.34;965.22) | 519.62 (424.6;635.92) | 359.84 (291.79;443.75) | 249.19 (190.25;326.38) | 172.56 (120.74;246.61) |
| 20-24 | 890.02 (646.16;1225.91) | 616.33 (488.05;778.34) | 426.81 (356.74;510.64) | 295.56 (244.73;356.95) | 204.68 (158.67;264.02) | 141.74 (100.27;200.35) |
| 25-29 | 731.04 (537.23;994.78) | 506.24 (407.44;629.01) | 350.57 (299.17;410.81) | 242.77 (204.81;287.76) | 168.12 (132.02;214.08) | 116.42 (83.1;163.1) |
| 30-34 | 600.46 (445.67;809.03) | 415.82 (339.23;509.69) | 287.95 (250.2;331.4) | 199.41 (170.89;232.68) | 138.09 (109.54;174.07) | 95.62 (68.71;133.07) |
| 35-39 | 493.21 (368.81;659.58) | 341.54 (281.53;414.36) | 236.52 (208.45;268.37) | 163.79 (142.02;188.89) | 113.42 (90.6;141.99) | 78.54 (56.69;108.83) |
| 40-44 | 405.11 (304.39;539.16) | 280.54 (232.75;338.14) | 194.27 (172.74;218.48) | 134.53 (117.43;154.12) | 93.16 (74.68;116.22) | 64.51 (46.65;89.22) |
| 45-49 | 332.75 (250.53;441.95) | 230.43 (191.62;277.1) | 159.57 (142.22;179.03) | 110.5 (96.54;126.48) | 76.52 (61.33;95.48) | 52.99 (38.3;73.32) |
| 50-54 | 273.31 (205.62;363.29) | 189.27 (157.07;228.07) | 131.07 (116.29;147.72) | 90.76 (78.9;104.41) | 62.85 (50.19;78.72) | 43.53 (31.36;60.41) |
| 55-59 | 224.49 (168.28;299.48) | 155.46 (128.21;188.51) | 107.66 (94.5;122.65) | 74.55 (64.15;86.64) | 51.63 (40.93;65.13) | 35.75 (25.62;49.89) |
| 60-64 | 184.4 (137.35;247.55) | 127.69 (104.25;156.41) | 88.43 (76.42;102.32) | 61.24 (51.92;72.21) | 42.4 (33.27;54.05) | 29.37 (20.88;41.3) |
| 65-69 | 151.46 (111.82;205.14) | 104.88 (84.49;130.2) | 72.63 (61.59;85.66) | 50.3 (41.89;60.39) | 34.83 (26.97;44.98) | 24.12 (16.98;34.26) |
| 70-74 | 124.4 (90.83;170.39) | 86.15 (68.29;108.68) | 59.66 (49.51;71.88) | 41.31 (33.72;50.62) | 28.61 (21.81;37.52) | 19.81 (13.78;28.47) |
| 75-79 | 102.18 (73.62;141.83) | 70.76 (55.08;90.91) | 49 (39.74;60.42) | 33.93 (27.09;42.5) | 23.5 (17.61;31.36) | 16.27 (11.17;23.71) |
| 80-85 | 83.93 (59.56;118.27) | 58.12 (44.34;76.19) | 40.25 (31.86;50.85) | 27.87 (21.74;35.74) | 19.3 (14.19;26.26) | 13.37 (9.04;19.77) |
| *Results from log-transformed models. Estimates are back-transformed. The results are estimated from the median age of the specific age spans and are presented as estimates with 95% CI.* | | | | | | |
